# Pollen Grain Classification Based on Ensemble Transfer Learning on the Cretan Pollen Dataset

**DOI:** 10.3390/plants11070919

**Published:** 2022-03-29

**Authors:** Nikos Tsiknakis, Elisavet Savvidaki, Georgios C. Manikis, Panagiota Gotsiou, Ilektra Remoundou, Kostas Marias, Eleftherios Alissandrakis, Nikolas Vidakis

**Affiliations:** 1 Computational Biomedicine Laboratory, Institute of Computer Science, Foundation for Research and Technology Hellas–FORTH, 70013 Heraklion, Greece; gmanikis@ics.forth.gr (G.C.M.); kmarias@ics.forth.gr (K.M.); 2 Department of Agriculture, Hellenic Mediterranean University, 71004 Heraklion, Greece; elisavvidaki@hmu.gr (E.S.); ealiss@hmu.gr (E.A.); 3 Department of Food Quality and Chemistry of Natural Products, Mediterranean Agronomic Institute of Chania (M.A.I.Ch./CIHEAM), 73100 Chania, Greece; yiota@maich.gr (P.G.); hlektra@maich.gr (I.R.); 4 Department of Electrical and Computer Engineering, Hellenic Mediterranean University, 71004 Heraklion, Greece; nv@hmu.gr

**Keywords:** pollen grain, classification, honey certification, melissopalynology, deep learning, transfer learning, ensemble

## Abstract

Pollen identification is an important task for the botanical certification of honey. It is performed via thorough microscopic examination of the pollen present in honey; a process called melissopalynology. However, manual examination of the images is hard, time-consuming and subject to inter- and intra-observer variability. In this study, we investigated the applicability of deep learning models for the classification of pollen-grain images into 20 pollen types, based on the Cretan Pollen Dataset. In particular, we applied transfer and ensemble learning methods to achieve an accuracy of 97.5%, a sensitivity of 96.9%, a precision of 97%, an F1 score of 96.89% and an AUC of 0.9995. However, in a preliminary case study, when we applied the best-performing model on honey-based pollen-grain images, we found that it performed poorly; only 0.02 better than random guessing (i.e., an AUC of 0.52). This indicates that the model should be further fine-tuned on honey-based pollen-grain images to increase its effectiveness on such data.

## 1. Introduction

Honey is a natural, sweet food produced by bees from the nectar of plants and/or from the secretions of plants and insects. It is a complex mixture, with excellent healing properties and nutrients, and plays an increasing part in the human diet [1,2]. Due to the great variety of melliferous plants, there is high variability among honeys regarding their composition. A honey can be considered to be unifloral or monofloral when honeybees collect mainly from one floral source. To certify the botanical origin of honey, physicochemical analyses are combined with the investigation of the pollen contained in the honey. Pollen analysis (also known as melissopalynology) is a procedure for identifying the pollen grains present in the honey sediment after dilution and centrifugation. The sediment is examined under a microscope by a specialist scientist, who identifies the granules according to their morphological characteristics. This process has been performed manually to date, although it is very time-consuming. For this reason, there is a need to find a method that will automate and speed up the process, while reducing the potential human error. Pollen analysis is a necessary procedure for characterizing the origin of honey. Among other things, geographical identification is possible, according to the flora of each place. Specifically, the region of Crete presents a unique floral diversity with a significant number of endemic plants from which honey is produced, with special organoleptic and physicochemical characteristics. Such products need protection to prevent fraud and the misleading of consumers.

Recent advances in artificial intelligence, especially deep learning, have enabled complex analyses to be performed in a variety of tasks, with impressive performances [3,4]. The successful development of robust and powerful deep learning models is dependent on the availability of large and well-annotated datasets, particularly for tasks based on supervised methods, such as pollen identification. However, the collection, curation and processing of pollen samples is difficult and time-consuming; thus, in comparison with other computer vision applications where large datasets exist, e.g., ImageNet [5], pollen-grain datasets are much smaller [6,7,8,9]. However, it is possible to leverage the obtained knowledge of models trained on larger datasets for other tasks, via transfer learning [10]. In the case where knowledge is transferred from models trained using ImageNet, which is a collection of more than 14 million real-life images with a thousand classes, when applied on datasets from another domain, the models are able to detect low-level imaging features more easily but are unable to properly perform the task at hand (i.e., pollen classification). It is necessary to perform fine-tuning by either retraining the existing classification part of the pretrained models or by training a new classifier on new annotated data, based on the deep features produced by the pretrained convolutional network. In this study, we utilized transfer learning to investigate the applicability of four well-established convolutional neural network (CNN) architectures, i.e., Inception v3 [11], Xception [12], ResNet [13] and Inception–ResNet [14] for pollen classification on the Cretan Pollen Dataset v1 (CPD-1) [6,15] which comprises more than 4000 pollen-grain images of 20 pollen types, gathered from the region of Crete. In addition, we examined two ensemble approaches to combine the predictive power of all or some of the base models. Finally, we obtained preliminary results from applying the best-performing model, trained on plant-based images, on pollen-grain images extracted from honey samples, to evaluate the applicability of such models in a real-life case such as pollen identification within the context of honey botanical certification.

## 2. Materials and Methods

### 2.1. Data

**Figure 1:**
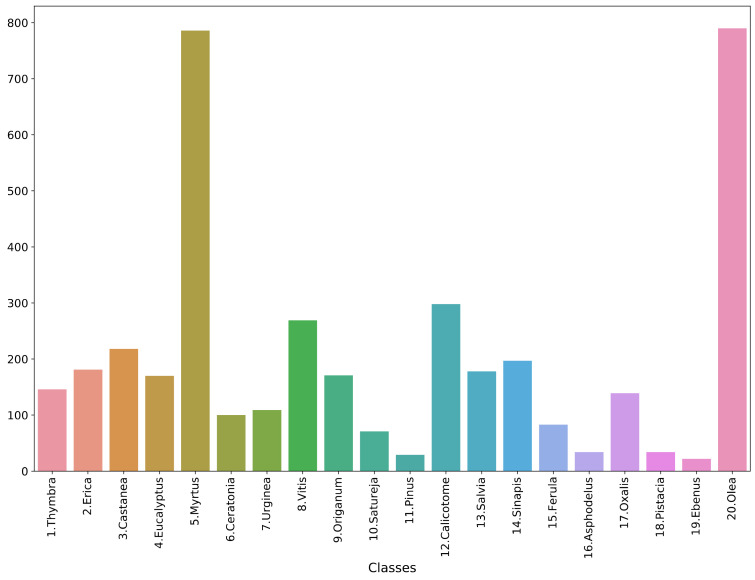
Data histogram across classes.

**Figure 2:**
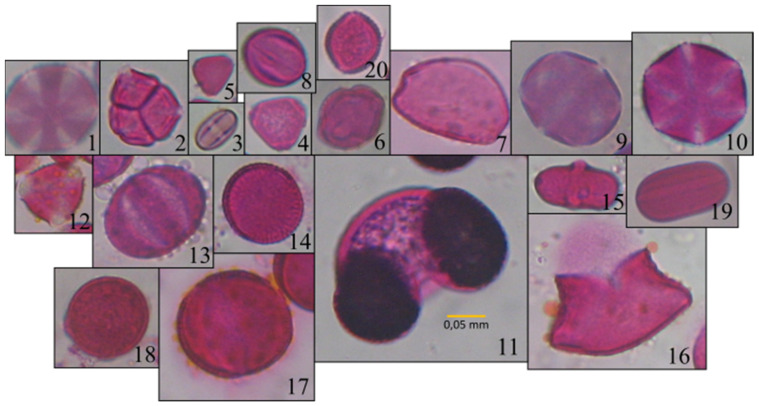
Mosaic of images of all pollen types: 1 Thymbra; 2 Erica; 3 Castanea; 4 Eucalyptus; 5 Myrtus; 6 Ceratonia; 7 Urginea; 8 Vitis; 9 Origanum; 10 Satureja; 11 Pinus; 12 Calicotome; 13 Salvia; 14 Sinapis; 15 Ferula; 16 Asphodelus; 17 Oxalis; 18 Pistacia; 19 Ebenus; 20 Olea.

**Figure 3:**
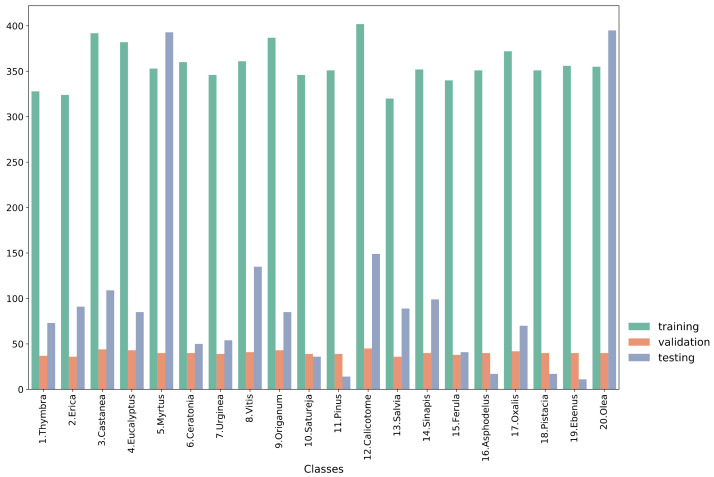
Histogram of each subset of the data.

The study used the Cretan Pollen Dataset v1 (CPD-1) [15] for developing the classification models. CPD-1 is a publicly available dataset comprising images of 4034 pollen grains of 20 plant species (Figure 1). The pollen samples were collected from various places in the region of Crete, Greece, during the period between April 2019 and April 2021. Figure 2 illustrates a mosaic of all pollen grains, numbered in accordance with Figure 1. This dataset is very rich in terms of the variety of pollen-grain types, comprising a wide spectrum of pollen types that are commonly found in Crete’s characteristic honey “Pefkothymaromelo Kritis PDO”. However, it also has some limitations such as a class imbalance, as seen in Figure 1, as well as some poorly segmented pollen grains. The proportion of poorly segmented pollen grains is very small, and we believe that it does not affect the training process of the model. In addition, some of the pollen types with a low representation in the dataset have a very distinct and unique morphology (e.g., *Pinus*). Thus, we believe that the model is able to classify these correctly, even if the number of images corresponding to these types is low. The dataset was split into three subsets, i.e., training, validation and hold-out testing sets (Figure 3). First, we split the dataset in half, to generate the training and testing sets. Then, the training set was augmented and split into final training and validation sets. The augmentation techniques applied to increase the size of the training set included randomly adding Gaussian noise, linearly adjusting contrast and brightness and rotating and translating the image in the ‘x’ and ‘y’ plane, as well as vertically and horizontally flipping the image (see Table 1 for a detailed presentation of the augmentation techniques). Finally, zero padding was applied to each image to standardize the image sizes to (512, 512) pixels. This padding was necessary because the largest pollen grain image has a size of (435, 419) pixels. Finally, each image was normalized by sample-wise centering of the mean value to 0 and dividing by the standard deviation of each sample.

### 2.2. Base Models

The current study utilized transfer learning to compare several well-established architectures that were pretrained on ImageNet [5]. In particular, the pretrained convolutional parts of the following architectures were used: Inception v3 [11], Xception [12], ResNet [13] and Inception–ResNet [14]. The ResNet architecture introduced residual connections to mitigate the vanishing/exploding gradients problem of deep neural networks. With its increased depth but considerably lower complexity than VGGNets, due to the use of global average pooling layers, ResNet was able to outperform the state-of-the-art approach at the time, overcoming multiple classification, detection and segmentation challenges. Google’s Inception architecture, on the other hand, is wider rather than deeper. It is built upon Inception modules, which consist of multiple parallel convolutional and pooling operations with different filter sizes, each of which computes a different transformation over the same input feature map. In addition, due to the increased computational complexity, 1 × 1 convolutions are used to reduce the dimensionality of the output feature maps. Inception v4, or the so-called Inception–ResNet architecture, combines the principles of Inception modules and residual connections to produce a wide, yet very deep CNN. Finally, Xception, which stands for extreme inception, uses depth-wise separable convolutions in place of the Inception modules, which effectively maps cross-channel and spatial correlations completely separately, while maintaining the complexity at the same level as Inception v3. Xception also uses linear residual connections, like ResNet.

A custom classification part was developed as described in Table 2, which followed the feature extraction part of the pretrained models. The GAP layer computes the mean value of each feature map, effectively downscaling and flattening the output of the convolutional network. For the dense layers, the activation function is the rectified linear unit (ReLU), while the dropout rate is chosen to be 50% for each neuron. The output dense layer utilizes the softmax activation function, which calculates the probability for each of the examined classes. Each model was trained for a maximum of 50 epochs, with early stopping based on the validation loss value. The categorical cross-entropy loss function (Equation (1)) and the Adam optimizer [16] with a batch size of 8 samples were used to train the model, using an exponentially decaying learning rate with an initial value of 0.001 and a decay rate of 0.96.

(1) $$CE=-\overset{C}{\underset{i=1}{\sum }}\;y_{o,c}log\left(o_{o,i}\right)\;$$

Here, *C* is the number of classes, *y* is a binary indicator of whether class label *c* is the correct classification for the observation *o* and *p* is the predicted probability that observation o belongs to class *i*. The model was trained on a server with an AMD EPYC 7251 8-core 2.9 GHz CPU, RTX 2080 Ti 11 GB GPU and 64 GB RAM and implemented using TensorFlow 2.5.

### 2.3. Ensemble Techniques

In addition, we utilized ensemble techniques to combine the predictive power of the models. The hypothesis was that each classification model identifies unique imaging features with respect to the other models, and thus the combination of their predictions boosts the overall classification performance. Two ensemble learning strategies were utilized: (a) a soft voting ensemble strategy, where the mean of the prediction probabilities of all models was calculated and used for the final prediction, and (b) a hard voting ensemble strategy, where each model calculated a single prediction and then the one with the maximum occurrences was used as the final prediction. An example of each strategy for a binary classification task is shown in Table 3.

## 3. Results

### 3.1. Performance Metrics

To evaluate the performance of the model on the hold-out testing set, we computed the following metrics based on the model predictions and the ground truth: accuracy (ACC), sensitivity (SEN), precision (PRE), F1 score and AUC score (one versus the rest). Because these metrics are mainly used in binary classification tasks, we computed them in two different settings: (a) an average calculation across all classes (macro and weighted averages) and (b) using a per-class calculation. In addition, we obtained the receiver operating characteristic (ROC) curve and the confusion matrix for each model. It should be noted that we have not provided the AUC and ROC curves for the hard voting ensemble models, because they provide class predictions rather than prediction probabilities; thus, only metrics derived from the confusion matrix are applicable.

### 3.2. Performance Analysis of the Models

Each model was trained to classify the pollen-grain images into one of the 20 plant species. The training, validation and testing sets comprised 7129, 802 and 2013 pollen-grain images, respectively. The specific distribution of each class is presented in Figure 3. The validation set was used for assessing the performance during training and for early stopping of the training procedure. Table 4 presents the overall performance results averaged across all classes for each of the models, while Table 5 presents the performance of each model regarding the Thymbra class. For the sake of brevity, the model performances on the rest of the classes are presented in their respective tables in the Supplementary Materials. We chose to include the performance regarding the Thymbra class in the main part of the paper because it is the main pollen type present in Crete’s trademark honey “Pefkothymaromelo Kritis PDO”.

Since our main objective was to correctly identify as many pollen grains belonging to the Thymbra class as possible, the metric that should be used to compare the model performances is the sensitivity (recall). At the same time, false positive cases for the Thymbra class should be minimized, which essentially means maximizing the specificity for that class. However, the perfect classifier, which would have a sensitivity of 1 and a specificity of 1, is rarely if ever observed in any real-life application in AI. To balance these two metrics, ROC curves and AUC scores are used. Thus, the metric that was used to compare the models and select the most appropriate model for our task was the AUC score of each model. However, because hard voting ensemble classifiers do not produce an ROC curve, based on which the AUC score is calculated, the comparison was based on the sensitivity.

**Figure 4:**
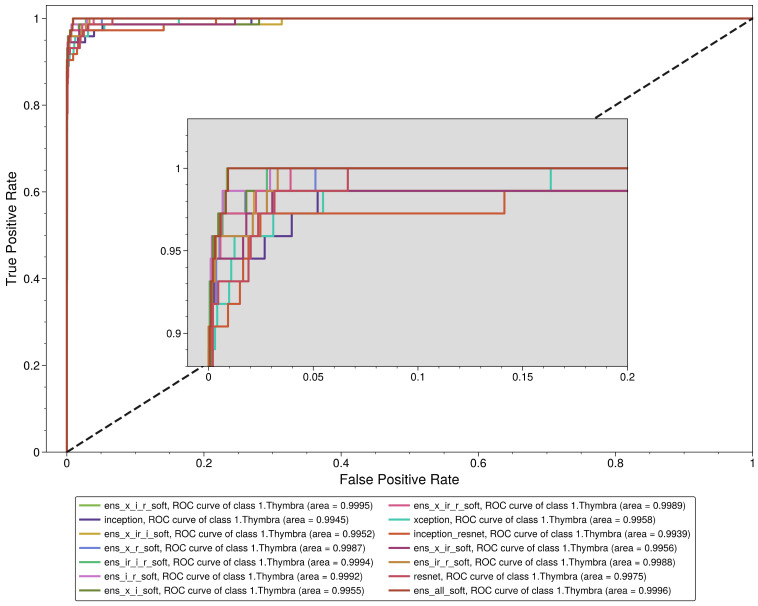
Receiver operating characteristic curve for *Thymbra* class.

We observe that all the models performed exceptionally well in the aggregated results (Table 4), with a weighted mean AUC value of 0.99895 ± 0.00054, a macro AUC value of 0.99898 ± 0.00047, a weighted mean sensitivity value of 0.96839 ± 0.00663 and a macro mean sensitivity value of 0.96237 ± 0.00739. However, the same does not apply for the *Thymbra* class evaluation. Although the mean AUC value of all models was 0.997295 ± 0.002001, the mean sensitivity was 0.895548 ± 0.04364. Interestingly, the base models (Inception v3, Inception–ResNet, Xception and ResNet) had a mean sensitivity value of 0.8869865 ± 0.0326692, the hard voting ensemble classifiers had a mean sensitivity value of 0.8712329 ± 0.050592 and the soft voting ensemble classifiers had a mean sensitivity value of 0.9232878 ± 0.0139697. In fact, the Xception–Inception soft voting ensemble model (“ens_x_i_soft”) had the highest sensitivity value of 0.945205 of all the models, while the highest sensitivity for a hard voting ensemble was 0.931507. Thus, soft voting ensemble classifiers should be preferred to hard voting classifiers in the context of this study. The receiver operating characteristic (ROC) analysis regarding the Thymbra class is presented in Figure 4.

**Figure 5:**
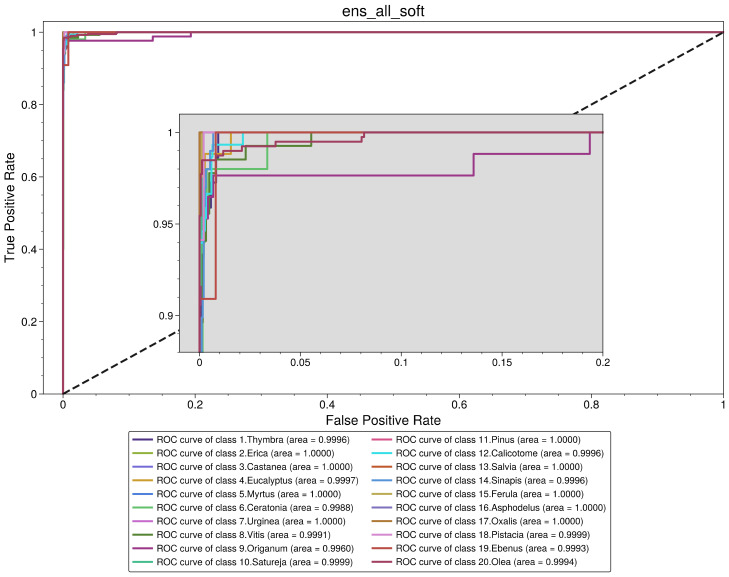
Receiver operating characteristic curve for soft voting ensemble of all base models.

**Figure 6:**
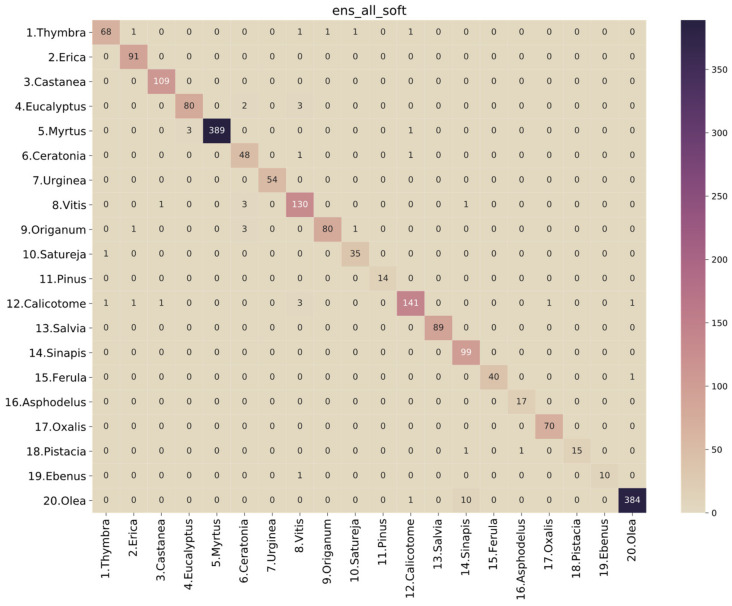
Confusion matrix for soft voting ensemble of all base models.

Thus, based on the previous assumptions and observations, the best-performing model is a soft voting ensemble of all the base models (“ens_all_soft”). The ROC analysis is presented in Figure 5, and the performance metrics are presented in Table 6. Looking at the confusion matrix of this classifier in Figure 6, we observe that there are only a few misclassified cases for all classes. Specifically, for *Thymbra*, there were 5 pollen-grain images that were classified as *Erica, Vitis, Origanum, Satureja* and *Calicotome*, while 68 were correctly classified as *Thymbra*. On the other hand, there were only 2 pollen-grain images that were falsely classified as *Thymbra*, when in fact they belonged to *Satureja* and *Calicotome* pollen types, respectively. Such misclassifications may be attributed to the fact that several pollen species such as *Thymbra*, *Origanum* and *Salvia* have shared morphological characteristics.

According to Greek legislation, at least 18% of the pollen found in the sediment of a sample must be thyme pollen for the honey to be characterized as thyme honey [17]. On this basis, if there are enough *Thymbra* pollen grains in the sample, the chosen model with a sensitivity of 0.931507 and a specificity of 0.998969 will identify more than enough pollen for the honey to be certified. However, extensive evaluation and possibly fine-tuning of the model on real-world honey-based samples should be carried out before any application of the model in a production environment.

The performance metrics and graphs for all the other models across all studied classes are included in the Supplementary Material.

## 4. Discussion

### 4.1. Comparison to Other Studies

Pollen analysis is an important task in melissopalynology, since it has a large financial impact on agricultural applications such as certification of botanical origin for honey. However, manual inspection of pollen microscopy images is a cumbersome and time-consuming task, subject to large inter- and intra-observer variability while achieving low identification accuracy. Thus, automatic pollen grain classification has attracted a great deal of attention from the research community during recent years.

In a previous study in our group, Manikis et al. [18] proposed a machine learning pipeline to extract geometric, textural and wavelet features from a private dataset of 564 pollen-grain images and to train a random forest classifier to classify them into six pollen types. They achieved a satisfactory performance, with a reported accuracy of 88.24%, precision of 88.60%, recall of 88.16% and an F1 score of 87.79%. Battiato et al. [19] presented a machine-learning-based analysis for classifying images into four classes (i.e., Corylus Avellana (well-developed pollen grains, anomalous pollen grains), Alnus and Debris). They investigated the performance of five classifiers based on hand-crafted features (i.e., HOG and LBP features), and they also developed an end-to-end model based on two convolutional neural network architectures, i.e., AlexNet and VGGNet. The best-performing model was AlexNet, achieving an accuracy of 0.8963 and a F1 score of 0.8897. A key difference between this study and our study is the increased number of unique pollen species that our dataset includes. Although the total number of pollen-grain images in our dataset was lower, our approach achieved a much better accuracy (0.975161) and F1 score (0.968880), with many more classes.

Although hand-crafted approaches report satisfactory performance results, we believe that models trained on selected hand-crafted features are inferior to deep learning approaches for large-scale tasks such as the one presented in this study. For reference, Battiato et al. [19] reported an increased performance from 3% up to 13% in terms of accuracy for a deep learning approach, in comparison to the hand-crafted approach. Sevillano et al. [20] presented a model combining CNN-based deep features with a linear discriminant classifier for classifying 23 types of pollen images on the Pollen23E dataset. Although this dataset comprises only 805 images (approximately 35 images per pollen type), it is very similar to ours regarding the high number of classes. Their approach achieved an accuracy of 0.932273, a precision of 0.9477, a recall (sensitivity) of 0.9964 and an F1 score of 0.9669. It is interesting to note that our approach slightly outperformed theirs, with an accuracy of 0.97561, precision of 0.970042, sensitivity of 0.969219 and an F1 score of 0.968880, indicating the robustness and wide applicability of advanced AI models on a wide spectrum of pollen samples.

Astolfi et al. [7] presented a very similar study to ours, as they trained and evaluated several well-established CNN architectures within the context of a large pollen-grain dataset. Specifically, their database comprised 2523 images of 73 pollen types collected from the Brazilian savanna (Cerrado). They reported that DenseNet-201 outperformed all the other models, achieving a precision score of 95.7%, an F1 score of 96.4%, an accuracy of 95.8% and a recall score of 95.7%. However, their models’ performances varied considerably across several pollen types. This may be attributed to the significantly lower number of images per class, i.e., there are many more classes but a much lower total number of images in the Pollen73S dataset, compared with the CPD v1 dataset. Finally, they conclude by stating the importance of investigating the potential gains of an ensemble approach, which, as our study indicates, can play an important role in achieving a performance boost compared to single-model approaches.

Table 7 presents the comparison information for the performance results of our method and those of other researchers.

### 4.2. Performance on Honey Data

**Figure 7:**
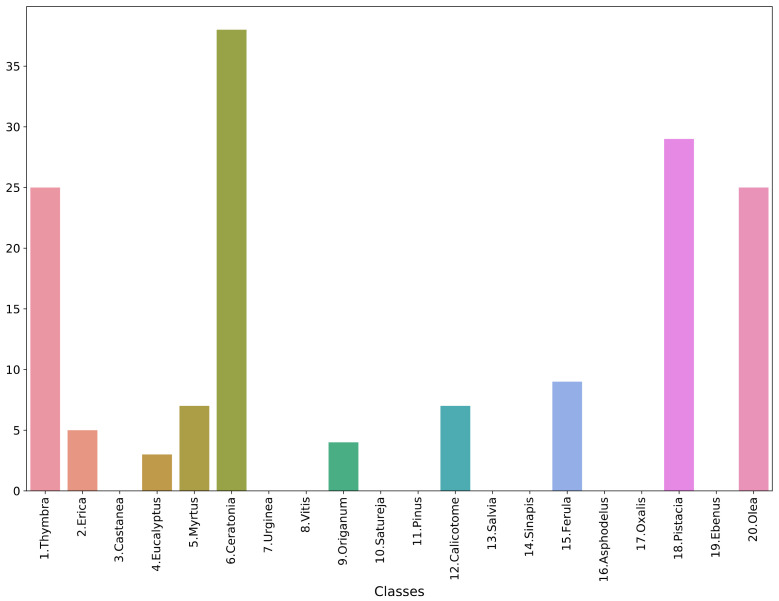
Histogram of honey-based dataset.

To evaluate the model in a real-life setting, 17 samples were collected from two different honey samples. Pollen grains were isolated according to [21]. In brief, 10 g of honey was dissolved in deionized water and centrifuged to remove the sugars. The sediment was used to make microscopic preparations with the addition of 0.05% pararosaniline chloride (Acros Organics, Mumbai, India). The slides were placed in a heating hearth at 40 °C to evaporate the moisture, followed by closing the sample using coverslips and mounting medium (Eukitt, Sigma-Aldrich, Tafkirchen, Germany). Permanent pollen preparations were allowed to dry before storage. The images were acquired using a Kern Optics microscope with a built-in ODC 832 camera, with 5.1 MP at 400× magnification. After the images were taken, the granules were identified by a specialist scientist. The images were processed to segment each pollen grain and then standardized according to the preprocessing pipeline of the analysis presented in Section 2. The final data comprised a total of 152 pollen-grain images, with a distribution across the classes as shown in Figure 7. This is a very imbalanced collection, with many classes not having a single data point. The best-performing model from the test set, i.e., the “ens_all_soft” classifier, was used to predict the classes of the pollen-grain images derived from the honey samples. It should be stressed that this is a preliminary case study, since the collected samples were not sufficient to produce reliable and trustworthy results. Hence, we chose to include it in the Discussion section of this manuscript as an indication of future research directions.

**Figure 8:**
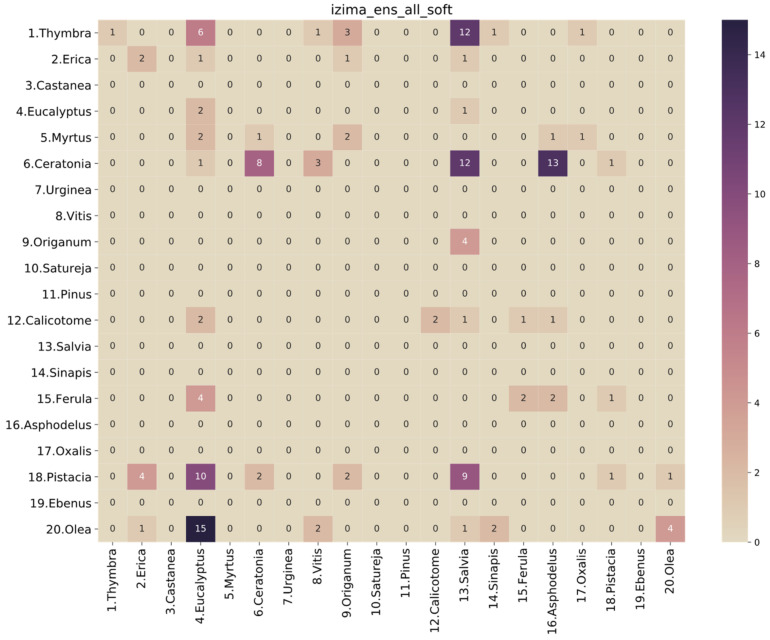
Confusion matrix of soft voting ensemble of all models on the honey-based dataset.

**Figure 9:**
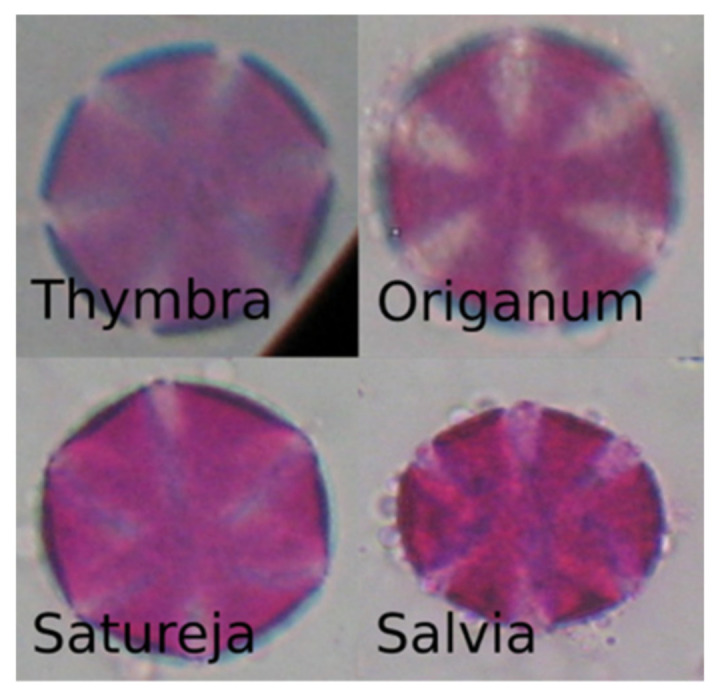
Pollen-grain images of pollen types with similar morphological characteristics.

Unfortunately, the results of the selected model on the honey-sampled pollen-grain images were very poor, with a weighted average AUC score of 0.52, which is only 2% better than a random prediction classifier. The confusion matrix in Figure 8 shows that there were many misclassifications, especially for the target species of this study, i.e., *Thymbra*. However, we can observe that most of the *Thymbra* samples were misclassified as *Salvia* and some as *Origanum*. These two, in addition to *Satureja*, have a very similar morphology to *Thymbra* (Figure 9 presents indicative pollen-grain images from these species sharing common morphological characteristics). We can also observe that all *Origanum* images were misclassified as *Salvia*. Taking into consideration the nature of this honey-based image collection and the morphological similarities between these species, these specific misclassifications could be expected.

However, there were still many unexpected misclassifications, which can be attributed to several factors. The images from the honey sediment showed some differences compared to those from the plant. It is possible that the granules in the sediment were more swollen, as they contained more water. At the same time, there is a possibility that many grains were joined together. Finally, honey contains other elements such as sugars and proteins, which can blur the images, affecting both the quality of the image and the integrity of the imaging features. In addition, the segmented pollen-grain images from the honey-based samples were unfortunately very few compared to the testing set of the initial plant-based data. Thus, any preliminary results presented in this discussion are not representative of the actual predictive power of the model. 

However, this analysis paves the way for future work on real-life data based on honey samples, while showing some necessary and important steps that must be followed in future studies. First, a larger number of samples should be collected and preprocessed to create large pollen-grain datasets from honey samples, which will be representative of all the pollen classes. Second, a robust and standardized preprocessing pipeline should be established to eliminate the variability between the pollen-grain images collected from plant samples and those collected from honey samples. Models that are trained on the plant-based dataset should be fine-tuned on a small subset of the honey-based dataset, to gain insight into this variability and learn the unique imaging features that may be present in the honey-based dataset. Following this, the fine-tuned models will be more useful to the melissopalynology community.

## 5. Conclusions

In this paper, we presented a comparative study for deep-learning-based classification of pollen-grain images from the 20 most common pollen species in Crete’s unique Pefkothymaromelo Kritis PDO honey. We compared four well-established CNN models, which were pretrained on the ImageNet database and fine-tuned on the publicly available dataset CPD v1 [15]. The best-performing model was based on the soft voting ensemble of all the base models, achieving an accuracy of 97.5%, a precision of 97%, a sensitivity of 96.9%, an F1 score of 96.89% and an AUC of 0.9995. When tested on a small collection of pollen grains extracted from honey samples, it performed poorly, as it only exceeded random guessing by 2% (i.e., an AUC of 0.52). Further development and fine-tuning on a larger honey-based collection should be performed to increase the model’s performance and robustness.

## Figures and Tables

**Table 1:** Augmentations used in the study.

Augmentation Method	Hyperparameters	Probability
Gaussian Blurring	Sigma [0, 0.3]	30%
Linear Contrast Adjustment	Alpha [0.75, 1.25]	30%
Brightness Multiplication	Multiplication factor [0.7, 1.3]	30%
Rotation	Degrees [−180, 180]	100%
Translation in x Plane	Translation percentage [−0.2, 0.2]	100%
Translation in y Plane	Translation percentage [−0.2, 0.2]	100%
Vertical Flipping	-	50%
Horizontal Flipping	-	50%

**Table 2:** Classification part of the network. The input and output sizes of the GAP layer, as well as the input size of the first dense layer, depend on the backbone convolutional network used.

	Input Size	Output Size	Activation Function
Global Average Pooling 2D	-	-	-
Dense Layer	-	1024	ReLU
Droput of 50%	1024	1024	-
Dense Layer	1024	512	ReLU
Droput of 50%	512	512	-
Dense Layer	512	256	ReLU
Droput of 50%	256	256	-
Dense Layer	256	128	ReLU
Droput of 50%	1024	1024	-
Dense Layer	128	20	Softmax

**Table 3:** Example of the ensemble strategies for a binary classification task. The same procedure applies for a 20-class task.

Models	Prediction Probability	Prediction
Model 1	[0.8, 0.2]	Class 0
Model 2	[0.55, 0.45]	Class 0
Model 3	[0.1, 0.9]	Class 1
Soft Voting Ensemble	[0.8 + 0.55 + 0.1, 0.2 + 0.45 + 0.9]/3 = [0.483, 0.517]	Class 1
Hard Voting Ensemble	Maximum occurrence [0,0,1]	Class 0

**Table 4:** Performance metrics for each model on the test set, averaged across all classes.

		Macro				Weighted			
	ACC	Pre	Sen	F1	AUC	Pre	Sen	F1	AUC
**ens_all_hard**	0.975161	0.970762	0.966647	0.967991	NA	0.976031	0.975161	0.975231	NA
**ens_all_soft**	0.975161	0.970042	0.969219	0.968880	**0.999542**	0.976306	0.975161	0.975334	**0.999533**
**ens_ir_i_r_hard**	0.972678	0.969362	0.966809	0.967251	NA	0.973837	0.972678	0.972838	NA
**ens_ir_i_r_soft**	0.973671	0.969781	0.966628	0.967443	0.999437	0.974688	0.973671	0.973803	0.999338
**ens_ir_r_hard**	0.957278	0.952362	0.947819	0.947840	NA	0.959952	0.957278	0.957202	NA
**ens_ir_r_soft**	0.966716	0.965132	0.963203	0.962795	0.999358	0.969911	0.966716	0.967410	0.999204
**ens_i_r_hard**	0.964729	0.959518	0.956076	0.956596	NA	0.966231	0.964729	0.964742	NA
**ens_i_r_soft**	0.974168	0.967764	0.970974	0.968863	0.999177	0.975065	0.974168	0.974326	0.999204
**ens_x_ir_hard**	0.959265	0.958602	0.949879	0.951970	NA	0.962451	0.959265	0.959344	NA
**ens_x_ir_i_hard**	0.972181	0.971409	0.969076	0.969541	NA	0.973656	0.972181	0.972402	NA
**ens_x_ir_i_soft**	0.974168	**0.971959**	0.968699	0.969395	0.999222	0.975805	0.974168	0.974393	0.999170
**ens_x_ir_r_hard**	0.969697	0.967619	0.964934	0.965034	NA	0.972198	0.969697	0.970201	NA
**ens_x_ir_r_soft**	0.971187	0.968954	0.966567	0.966683	0.999464	0.973259	0.971187	0.971571	0.999475
**ens_x_ir_soft**	0.966716	0.963960	0.961913	0.961237	0.998892	0.970550	0.966716	0.967457	0.998980
**ens_x_i_hard**	0.965723	0.960852	0.954880	0.956783	NA	0.966856	0.965723	0.965620	NA
**ens_x_i_r_hard**	0.976155	0.969923	0.970366	0.969455	NA	0.977386	0.976155	0.976399	NA
**ens_x_i_r_soft**	**0.976652**	0.969540	0.971659	**0.969963**	0.999387	**0.977790**	**0.976652**	**0.976889**	0.999454
**ens_x_i_soft**	0.974168	0.967077	**0.971752**	0.968744	0.998931	0.975470	0.974168	0.974411	0.999008
**ens_x_r_hard**	0.967213	0.960367	0.952631	0.954955	NA	0.968760	0.967213	0.967131	NA
**ens_x_r_soft**	0.971684	0.964913	0.963993	0.963454	0.999097	0.973144	0.971684	0.971921	0.999184
**inception**	0.964729	0.960787	0.961660	0.960547	0.998633	0.966119	0.964729	0.964959	0.998587
**inception_resnet**	0.952310	0.953253	0.952506	0.950253	0.998199	0.958644	0.952310	0.953534	0.997607
**resnet**	0.958271	0.950490	0.955365	0.951001	0.998360	0.962226	0.958271	0.959258	0.998233
**xception**	0.961749	0.950759	0.953611	0.950483	0.998096	0.964355	0.961749	0.962129	0.998363

Individual models (short name for ensemble naming): Inception (i); ResNet (r); Inception–ResNet (ir); Xception (x). Ensemble strategies: soft or hard voting. The naming of the ensemble models is based on the schema “ens_MODELS_VOTING” (e.g., ens_i_r_soft means Inception and ResNet ensembles based on soft voting). NA refers to Not Applicable.

**Table 5:** Performance metrics for each model on the test set regarding the *Thymbra* class.

	Sensitivity	Specificity	Precision	Accuracy	F1	AUC
**ens_all_hard**	0.917808	0.998969	0.971014	0.996026	0.943662	NA
**ens_all_soft**	0.931507	0.998969	0.971429	0.996523	0.951049	**0.999569**
**ens_ir_i_r_hard**	0.917808	0.998969	0.971014	0.996026	0.943662	NA
**ens_ir_i_r_soft**	0.931507	0.998969	0.971429	0.996523	0.951049	0.999364
**ens_ir_r_hard**	0.780822	**1.000000**	**1.000000**	0.992052	0.876923	NA
**ens_ir_r_soft**	0.917808	**1.000000**	**1.000000**	**0.997019**	**0.957143**	0.998757
**ens_i_r_hard**	0.835616	0.998969	0.968254	0.993045	0.897059	NA
**ens_i_r_soft**	0.931507	0.997938	0.944444	0.995529	0.937931	0.999244
**ens_x_ir_hard**	0.821918	**1.000000**	**1.000000**	0.993542	0.902256	NA
**ens_x_ir_i_hard**	0.904110	0.998454	0.956522	0.995032	0.929577	NA
**ens_x_ir_i_soft**	0.931507	0.998969	0.971429	0.996523	0.951049	0.995220
**ens_x_ir_r_hard**	0.917808	0.998969	0.971014	0.996026	0.943662	NA
**ens_x_ir_r_soft**	0.904110	0.999485	0.985075	0.996026	0.942857	0.998913
**ens_x_ir_soft**	0.904110	**1.000000**	**1.000000**	0.996523	0.949640	0.995629
**ens_x_i_hard**	0.863014	0.998454	0.954545	0.993542	0.906475	NA
**ens_x_i_r_hard**	0.931507	0.998454	0.957746	0.996026	0.944444	NA
**ens_x_i_r_soft**	0.931507	0.998969	0.971429	0.996523	0.951049	0.999477
**ens_x_i_soft**	**0.945205**	0.996907	0.920000	0.995032	0.932432	0.995523
**ens_x_r_hard**	0.821918	0.998969	0.967742	0.992548	0.888889	NA
**ens_x_r_soft**	0.904110	0.998969	0.970588	0.995529	0.936170	0.998687
**inception**	0.931507	0.995361	0.883117	0.993045	0.906667	0.994549
**inception_resnet**	0.849315	**1.000000**	**1.000000**	0.994536	0.918519	0.993913
**resnet**	0.863014	0.997938	0.940299	0.993045	0.900000	0.997522
**xception**	0.904110	0.996907	0.916667	0.993542	0.910345	0.995763

**Table 6:** Performance results of soft voting ensemble of all base models across all classes.

	Sensitivity	Specificity	Precision	Accuracy	F1	AUC
**1. Thymbra**	0.931507	0.998969	0.971429	0.996523	0.951049	0.999569
**2. Erica**	1.000000	0.998439	0.968085	0.998510	0.983784	1.000000
**3. Castanea**	1.000000	0.998950	0.981982	0.999006	0.990909	1.000000
**4. Eucalyptus**	0.941176	0.998444	0.963855	0.996026	0.952381	0.999713
**5. Myrtus**	0.989822	1.000000	1.000000	0.998013	0.994885	0.999991
**6. Ceratonia**	0.960000	0.995925	0.857143	0.995032	0.905660	0.998839
**7. Urginea**	1.000000	1.000000	1.000000	1.000000	1.000000	1.000000
**8. Vitis**	0.962963	0.995208	0.935252	0.993045	0.948905	0.999101
**9. Origanum**	0.941176	0.999481	0.987654	0.997019	0.963855	0.995973
**10. Satureja**	0.972222	0.998988	0.945946	0.998510	0.958904	0.999930
**11. Pinus**	1.000000	1.000000	1.000000	1.000000	1.000000	1.000000
**12. Calicotome**	0.946309	0.997854	0.972414	0.994039	0.959184	0.999622
**13. Salvia**	1.000000	1.000000	1.000000	1.000000	1.000000	1.000000
**14. Sinapis**	1.000000	0.993730	0.891892	0.994039	0.942857	0.999609
**15. Ferula**	0.975610	1.000000	1.000000	0.999503	0.987654	0.999975
**16. Asphodelus**	1.000000	0.999499	0.944444	0.999503	0.971429	1.000000
**17. Oxalis**	1.000000	0.999485	0.985915	0.999503	0.992908	1.000000
**18. Pistacia**	0.882353	1.000000	1.000000	0.999006	0.937500	0.999882
**19. Ebenus**	0.909091	1.000000	1.000000	0.999503	0.952381	0.999273
**20. Olea**	0.972152	0.998764	0.994819	0.993542	0.983355	0.999368

**Table 7:** Comparison table between other studies and ours.

Ref.	Method	Dataset	AUC	Sensitivity	Precision	Accuracy	F1 Score
Manikis et al. [18]	Hand-crafted Features + ML	546 images	-	88.16%	88.60%	88.24%	87.79%
Battiato et al. [19]	CNN	Pollen23E 805 images	-	-	-	89.63%	88.97%
Sevillano et al. [20]	CNN + LD	Pollen23E 805 images	-	99.64%	94.77%	93.22%	96.69%
Astolfi et al. [7]	CNN	Pollen73S 2523 images	-	95.7%	95.7%	95.8%	96.4%
Our study	CNN	CPD 4034	0.9995	96.9%	97%	97.5%	96.89%

## Data Availability

The data used in this study are available on Zenodo at https://doi.org/10.5281/zenodo.4756360 (accessed on 28 March 2022).

## Supplementary Materials

The following supporting information can be downloaded at: https://www.mdpi.com/article/10.3390/plants11070919/s1.

## References

[1] Ilia G., Simulescu V., Merghes P., Varan N. (2021). The health benefits of honey as an energy source with antioxidant, antibacterial and antiseptic effects. Sci. Sports.

[2] Majtan J., Bucekova M., Kafantaris I., Szweda P., Hammer K., Mossialos D. (2021). Honey antibacterial activity: A neglected aspect of honey quality assurance as functional food. Trends Food Sci. Technol..

[3] Esteva A., Chou K., Yeung S., Naik N., Madani A., Mottaghi A., Liu Y., Topol E., Dean J., Socher R. (2021). Deep learning-enabled medical computer vision. NPJ Digit. Med..

[4] Santos L., Santos F.N., Oliveira P.M., Shinde P. (2019). Deep learning applications in agriculture: A short review. Iberian Robotics Conference.

[5] Deng J., Dong W., Socher R., Li L.-J., Li K., Fei-Fei L. ImageNet: A large-scale hierarchical image database. Proceedings of the 2009 IEEE Conference on Computer Vision and Pattern Recognition.

[6] Tsiknakis N., Savvidaki E., Kafetzopoulos S., Manikis G., Vidakis N., Marias K., Alissandrakis E. (2021). Cretan Pollen Dataset v1 (CPD-1). E. Cretan Pollen Dataset.

[7] Astolfi G., Gonçalves A.B., Menezes G.V., Borges F.S.B., Astolfi A.C.M.N., Matsubara E.T., Alvarez M., Pistori H. (2020). POLLEN73S: An image dataset for pollen grains classification. Ecol. Inform..

[8] Gonçalves A.B., Souza J.S., Da Silva G.G., Cereda M.P., Pott A., Naka M.H., Pistori H. (2016). Feature Extraction and Machine Learning for the Classification of Brazilian Savannah Pollen Grains. PLoS ONE.

[9] Battiato S., Ortis A., Trenta F., Ascari L., Politi M., Siniscalco C. (2020). POLLEN13K: A Large Scale Microscope Pollen Grain Image Dataset. Proceedings of the 2020 IEEE International Conference on Image Processing (ICIP).

[10] Goodfellow I., Bengio Y., Courville A. (2016). Deep Learning.

[11] Szegedy C., Vanhoucke V., Ioffe S., Shlens J., Wojna Z. Rethinking the Inception Architecture for Computer Vision. Proceedings of the IEEE Conference on Computer Vision and Pattern Recognition (CVPR).

[12] Chollet F. Xception: Deep learning with depthwise separable convolutions. Proceedings of the 2017 IEEE Conference on Computer Vision and Pattern Recognition (CVPR).

[13] He K., Zhang X., Ren S., Sun J. Deep residual learning for image recognition. Proceedings of the 2016 IEEE Conference on Computer Vision and Pattern Recognition (CVPR).

[14] Szegedy C., Ioffe S., Vanhoucke V., Alemi A.A. (2017). Inception-v4, Inception-ResNet and the Impact of Residual Connections on Learning.

[15] Tsiknakis N., Savvidaki E., Kafetzopoulos S., Manikis G., Vidakis N., Marias K., Alissandrakis E. (2021). Segmenting 20 Types of Pollen Grains for the Cretan Pollen Dataset v1 (CPD-1). Appl. Sci..

[16] Kingma D.P., Ba J. Adam: A method for stochastic optimization. Proceedings of the International Conference Learn, Represent, (ICLR).

[17] Official Government Gazette B-239/23-2-2005 Annex II Article 67 of Greek Food Code 2005, Greek Ministry of Agriculture. http://www.et.gr/index.php/anazitisi-fek.

[18] Manikis G.C., Marias K., Alissandrakis E., Perrotto L., Savvidaki E., Vidakis N. (2019). Pollen Grain Classification using Geometrical and Textural Features. Proceedings of the 2019 IEEE International Conference on Imaging Systems and Techniques (IST).

[19] Battiato S., Ortis A., Trenta F., Ascari L., Politi M., Siniscalco C. Detection and Classification of Pollen Grain Microscope Images. Proceedings of the 2020 IEEE/CVF Conference on Computer Vision and Pattern Recognition Workshops (CVPRW).

[20] Sevillano V., Aznarte J.L. (2018). Improving classification of pollen grain images of the POLEN23E dataset through three different applications of deep learning convolutional neural networks. PLoS ONE.

[21] Louveaux J., Maurizio A., Vorwohl G. (1978). Methods of Melissopalynology. BEE World.

