# Supplementary material for: Pollen Grain Classification Based on Ensemble Transfer Learning on the Cretan Pollen Dataset
Source: Plants (Basel). 2022 Mar 29;11(7):919. doi: 10.3390/plants11070919 (PMC9002917; doi:10.3390/plants11070919)
Supplement: Supplementary file 1 [file plants-11-00919-s001.zip › Supplementary-Images/tables-results-of-all-models/ens_x_i_r_hard_metrics.html]

|  | sensitivity | specificity | precision | accuracy | f1 | auc |
| --- | --- | --- | --- | --- | --- | --- |
| 1.Thymbra | 0.931507 | 0.998454 | 0.957746 | 0.996026 | 0.944444 | nan |
| 2.Erica | 1.000000 | 0.999480 | 0.989130 | 0.999503 | 0.994536 | nan |
| 3.Castanea | 1.000000 | 0.998950 | 0.981982 | 0.999006 | 0.990909 | nan |
| 4.Eucalyptus | 0.929412 | 0.999481 | 0.987500 | 0.996523 | 0.957576 | nan |
| 5.Myrtus | 0.997455 | 1.000000 | 1.000000 | 0.999503 | 0.998726 | nan |
| 6.Ceratonia | 0.940000 | 0.993887 | 0.796610 | 0.992548 | 0.862385 | nan |
| 7.Urginea | 1.000000 | 1.000000 | 1.000000 | 1.000000 | 1.000000 | nan |
| 8.Vitis | 0.962963 | 0.995740 | 0.942029 | 0.993542 | 0.952381 | nan |
| 9.Origanum | 0.941176 | 0.998963 | 0.975610 | 0.996523 | 0.958084 | nan |
| 10.Satureja | 0.972222 | 0.998988 | 0.945946 | 0.998510 | 0.958904 | nan |
| 11.Pinus | 1.000000 | 1.000000 | 1.000000 | 1.000000 | 1.000000 | nan |
| 12.Calicotome | 0.939597 | 0.998927 | 0.985915 | 0.994536 | 0.962199 | nan |
| 13.Salvia | 1.000000 | 1.000000 | 1.000000 | 1.000000 | 1.000000 | nan |
| 14.Sinapis | 0.989899 | 0.995298 | 0.915888 | 0.995032 | 0.951456 | nan |
| 15.Ferula | 0.975610 | 1.000000 | 1.000000 | 0.999503 | 0.987654 | nan |
| 16.Asphodelus | 1.000000 | 0.999499 | 0.944444 | 0.999503 | 0.971429 | nan |
| 17.Oxalis | 1.000000 | 0.999485 | 0.985915 | 0.999503 | 0.992908 | nan |
| 18.Pistacia | 0.941176 | 1.000000 | 1.000000 | 0.999503 | 0.969697 | nan |
| 19.Ebenus | 0.909091 | 1.000000 | 1.000000 | 0.999503 | 0.952381 | nan |
| 20.Olea | 0.977215 | 0.997528 | 0.989744 | 0.993542 | 0.983439 | nan |
