# Supplementary material for: Pollen Grain Classification Based on Ensemble Transfer Learning on the Cretan Pollen Dataset
Source: Plants (Basel). 2022 Mar 29;11(7):919. doi: 10.3390/plants11070919 (PMC9002917; doi:10.3390/plants11070919)
Supplement: Supplementary file 1 [file plants-11-00919-s001.zip › Supplementary-Images/tables-results-of-all-models/xception_metrics.html]

|  | sensitivity | specificity | precision | accuracy | f1 | auc |
| --- | --- | --- | --- | --- | --- | --- |
| 1.Thymbra | 0.904110 | 0.996907 | 0.916667 | 0.993542 | 0.910345 | 0.995763 |
| 2.Erica | 1.000000 | 0.996358 | 0.928571 | 0.996523 | 0.962963 | 0.999989 |
| 3.Castanea | 1.000000 | 0.998950 | 0.981982 | 0.999006 | 0.990909 | 0.999735 |
| 4.Eucalyptus | 0.823529 | 0.997407 | 0.933333 | 0.990065 | 0.875000 | 0.994685 |
| 5.Myrtus | 0.989822 | 1.000000 | 1.000000 | 0.998013 | 0.994885 | 0.999797 |
| 6.Ceratonia | 0.960000 | 0.992359 | 0.761905 | 0.991555 | 0.849558 | 0.996877 |
| 7.Urginea | 1.000000 | 1.000000 | 1.000000 | 1.000000 | 1.000000 | 1.000000 |
| 8.Vitis | 0.962963 | 0.995208 | 0.935252 | 0.993045 | 0.948905 | 0.996551 |
| 9.Origanum | 0.941176 | 0.998963 | 0.975610 | 0.996523 | 0.958084 | 0.993343 |
| 10.Satureja | 0.972222 | 0.997471 | 0.875000 | 0.997019 | 0.921053 | 0.997513 |
| 11.Pinus | 0.928571 | 1.000000 | 1.000000 | 0.999503 | 0.962963 | 0.997642 |
| 12.Calicotome | 0.939597 | 0.998391 | 0.979021 | 0.994039 | 0.958904 | 0.997141 |
| 13.Salvia | 0.977528 | 0.998960 | 0.977528 | 0.998013 | 0.977528 | 0.999825 |
| 14.Sinapis | 0.979798 | 0.992163 | 0.866071 | 0.991555 | 0.919431 | 0.998987 |
| 15.Ferula | 0.975610 | 1.000000 | 1.000000 | 0.999503 | 0.987654 | 0.999604 |
| 16.Asphodelus | 1.000000 | 0.998998 | 0.894737 | 0.999006 | 0.944444 | 1.000000 |
| 17.Oxalis | 0.971429 | 1.000000 | 1.000000 | 0.999006 | 0.985507 | 1.000000 |
| 18.Pistacia | 0.882353 | 1.000000 | 1.000000 | 0.999006 | 0.937500 | 0.996758 |
| 19.Ebenus | 0.909091 | 1.000000 | 1.000000 | 0.999503 | 0.952381 | 0.998956 |
| 20.Olea | 0.954430 | 0.997528 | 0.989501 | 0.989071 | 0.971649 | 0.998759 |
