# Supplementary material for: Pollen Grain Classification Based on Ensemble Transfer Learning on the Cretan Pollen Dataset
Source: Plants (Basel). 2022 Mar 29;11(7):919. doi: 10.3390/plants11070919 (PMC9002917; doi:10.3390/plants11070919)
Supplement: Supplementary file 1 [file plants-11-00919-s001.zip › Supplementary-Images/tables-results-of-all-models/ens_ir_i_r_hard_metrics.html]

|  | sensitivity | specificity | precision | accuracy | f1 | auc |
| --- | --- | --- | --- | --- | --- | --- |
| 1.Thymbra | 0.917808 | 0.998969 | 0.971014 | 0.996026 | 0.943662 | nan |
| 2.Erica | 1.000000 | 0.998959 | 0.978495 | 0.999006 | 0.989130 | nan |
| 3.Castanea | 1.000000 | 0.998950 | 0.981982 | 0.999006 | 0.990909 | nan |
| 4.Eucalyptus | 0.929412 | 0.998963 | 0.975309 | 0.996026 | 0.951807 | nan |
| 5.Myrtus | 0.989822 | 0.999383 | 0.997436 | 0.997516 | 0.993614 | nan |
| 6.Ceratonia | 0.960000 | 0.994906 | 0.827586 | 0.994039 | 0.888889 | nan |
| 7.Urginea | 1.000000 | 1.000000 | 1.000000 | 1.000000 | 1.000000 | nan |
| 8.Vitis | 0.925926 | 0.995208 | 0.932836 | 0.990561 | 0.929368 | nan |
| 9.Origanum | 0.952941 | 0.998444 | 0.964286 | 0.996523 | 0.958580 | nan |
| 10.Satureja | 0.972222 | 0.999494 | 0.972222 | 0.999006 | 0.972222 | nan |
| 11.Pinus | 1.000000 | 1.000000 | 1.000000 | 1.000000 | 1.000000 | nan |
| 12.Calicotome | 0.946309 | 0.998391 | 0.979167 | 0.994536 | 0.962457 | nan |
| 13.Salvia | 1.000000 | 1.000000 | 1.000000 | 1.000000 | 1.000000 | nan |
| 14.Sinapis | 1.000000 | 0.993730 | 0.891892 | 0.994039 | 0.942857 | nan |
| 15.Ferula | 0.975610 | 1.000000 | 1.000000 | 0.999503 | 0.987654 | nan |
| 16.Asphodelus | 1.000000 | 0.999499 | 0.944444 | 0.999503 | 0.971429 | nan |
| 17.Oxalis | 1.000000 | 0.999485 | 0.985915 | 0.999503 | 0.992908 | nan |
| 18.Pistacia | 0.882353 | 1.000000 | 1.000000 | 0.999006 | 0.937500 | nan |
| 19.Ebenus | 0.909091 | 1.000000 | 1.000000 | 0.999503 | 0.952381 | nan |
| 20.Olea | 0.974684 | 0.996292 | 0.984655 | 0.992052 | 0.979644 | nan |
