# Supplementary material for: Pollen Grain Classification Based on Ensemble Transfer Learning on the Cretan Pollen Dataset
Source: Plants (Basel). 2022 Mar 29;11(7):919. doi: 10.3390/plants11070919 (PMC9002917; doi:10.3390/plants11070919)
Supplement: Supplementary file 1 [file plants-11-00919-s001.zip › Supplementary-Images/tables-results-of-all-models/ens_ir_i_r_soft_metrics.html]

|  | sensitivity | specificity | precision | accuracy | f1 | auc |
| --- | --- | --- | --- | --- | --- | --- |
| 1.Thymbra | 0.931507 | 0.998969 | 0.971429 | 0.996523 | 0.951049 | 0.999364 |
| 2.Erica | 1.000000 | 0.998959 | 0.978495 | 0.999006 | 0.989130 | 0.999983 |
| 3.Castanea | 1.000000 | 0.997899 | 0.964602 | 0.998013 | 0.981982 | 1.000000 |
| 4.Eucalyptus | 0.917647 | 0.998963 | 0.975000 | 0.995529 | 0.945455 | 0.999805 |
| 5.Myrtus | 0.989822 | 0.999383 | 0.997436 | 0.997516 | 0.993614 | 0.999983 |
| 6.Ceratonia | 0.960000 | 0.995415 | 0.842105 | 0.994536 | 0.897196 | 0.998828 |
| 7.Urginea | 1.000000 | 1.000000 | 1.000000 | 1.000000 | 1.000000 | 1.000000 |
| 8.Vitis | 0.948148 | 0.994675 | 0.927536 | 0.991555 | 0.937729 | 0.999034 |
| 9.Origanum | 0.952941 | 0.998444 | 0.964286 | 0.996523 | 0.958580 | 0.996266 |
| 10.Satureja | 0.944444 | 0.999494 | 0.971429 | 0.998510 | 0.957746 | 0.999930 |
| 11.Pinus | 1.000000 | 1.000000 | 1.000000 | 1.000000 | 1.000000 | 1.000000 |
| 12.Calicotome | 0.946309 | 0.997854 | 0.972414 | 0.994039 | 0.959184 | 0.998092 |
| 13.Salvia | 1.000000 | 1.000000 | 1.000000 | 1.000000 | 1.000000 | 1.000000 |
| 14.Sinapis | 1.000000 | 0.994775 | 0.908257 | 0.995032 | 0.951923 | 0.999578 |
| 15.Ferula | 0.975610 | 1.000000 | 1.000000 | 0.999503 | 0.987654 | 0.999975 |
| 16.Asphodelus | 1.000000 | 0.999499 | 0.944444 | 0.999503 | 0.971429 | 1.000000 |
| 17.Oxalis | 1.000000 | 0.999485 | 0.985915 | 0.999503 | 0.992908 | 0.999926 |
| 18.Pistacia | 0.882353 | 1.000000 | 1.000000 | 0.999006 | 0.937500 | 0.999882 |
| 19.Ebenus | 0.909091 | 1.000000 | 1.000000 | 0.999503 | 0.952381 | 0.999137 |
| 20.Olea | 0.974684 | 0.998146 | 0.992268 | 0.993542 | 0.983397 | 0.998964 |
