# Supplementary material for: Pollen Grain Classification Based on Ensemble Transfer Learning on the Cretan Pollen Dataset
Source: Plants (Basel). 2022 Mar 29;11(7):919. doi: 10.3390/plants11070919 (PMC9002917; doi:10.3390/plants11070919)
Supplement: Supplementary file 1 [file plants-11-00919-s001.zip › Supplementary-Images/tables-results-of-all-models/ens_ir_r_hard_metrics.html]

|  | sensitivity | specificity | precision | accuracy | f1 | auc |
| --- | --- | --- | --- | --- | --- | --- |
| 1.Thymbra | 0.780822 | 1.000000 | 1.000000 | 0.992052 | 0.876923 | nan |
| 2.Erica | 0.978022 | 0.999480 | 0.988889 | 0.998510 | 0.983425 | nan |
| 3.Castanea | 1.000000 | 0.999475 | 0.990909 | 0.999503 | 0.995434 | nan |
| 4.Eucalyptus | 0.788235 | 0.998444 | 0.957143 | 0.989568 | 0.864516 | nan |
| 5.Myrtus | 0.984733 | 0.999383 | 0.997423 | 0.996523 | 0.991037 | nan |
| 6.Ceratonia | 0.920000 | 0.991340 | 0.730159 | 0.989568 | 0.814159 | nan |
| 7.Urginea | 1.000000 | 0.999490 | 0.981818 | 0.999503 | 0.990826 | nan |
| 8.Vitis | 0.918519 | 0.993078 | 0.905109 | 0.988077 | 0.911765 | nan |
| 9.Origanum | 0.952941 | 0.996369 | 0.920455 | 0.994536 | 0.936416 | nan |
| 10.Satureja | 1.000000 | 0.998483 | 0.923077 | 0.998510 | 0.960000 | nan |
| 11.Pinus | 1.000000 | 1.000000 | 1.000000 | 1.000000 | 1.000000 | nan |
| 12.Calicotome | 0.932886 | 0.998391 | 0.978873 | 0.993542 | 0.955326 | nan |
| 13.Salvia | 0.988764 | 0.999480 | 0.988764 | 0.999006 | 0.988764 | nan |
| 14.Sinapis | 0.959596 | 0.992163 | 0.863636 | 0.990561 | 0.909091 | nan |
| 15.Ferula | 0.975610 | 0.998986 | 0.952381 | 0.998510 | 0.963855 | nan |
| 16.Asphodelus | 1.000000 | 0.999499 | 0.944444 | 0.999503 | 0.971429 | nan |
| 17.Oxalis | 1.000000 | 0.998456 | 0.958904 | 0.998510 | 0.979021 | nan |
| 18.Pistacia | 0.882353 | 1.000000 | 1.000000 | 0.999006 | 0.937500 | nan |
| 19.Ebenus | 0.909091 | 1.000000 | 1.000000 | 0.999503 | 0.952381 | nan |
| 20.Olea | 0.984810 | 0.991347 | 0.965261 | 0.990065 | 0.974937 | nan |
