# Supplementary material for: Pollen Grain Classification Based on Ensemble Transfer Learning on the Cretan Pollen Dataset
Source: Plants (Basel). 2022 Mar 29;11(7):919. doi: 10.3390/plants11070919 (PMC9002917; doi:10.3390/plants11070919)
Supplement: Supplementary file 1 [file plants-11-00919-s001.zip › Supplementary-Images/tables-results-of-all-models/ens_x_r_soft_metrics.html]

|  | sensitivity | specificity | precision | accuracy | f1 | auc |
| --- | --- | --- | --- | --- | --- | --- |
| 1.Thymbra | 0.904110 | 0.998969 | 0.970588 | 0.995529 | 0.936170 | 0.998687 |
| 2.Erica | 1.000000 | 0.997919 | 0.957895 | 0.998013 | 0.978495 | 0.999989 |
| 3.Castanea | 1.000000 | 0.998424 | 0.973214 | 0.998510 | 0.986425 | 1.000000 |
| 4.Eucalyptus | 0.894118 | 0.998444 | 0.962025 | 0.994039 | 0.926829 | 0.996217 |
| 5.Myrtus | 0.994911 | 0.999383 | 0.997449 | 0.998510 | 0.996178 | 0.999992 |
| 6.Ceratonia | 0.940000 | 0.992868 | 0.770492 | 0.991555 | 0.846847 | 0.997738 |
| 7.Urginea | 1.000000 | 1.000000 | 1.000000 | 1.000000 | 1.000000 | 1.000000 |
| 8.Vitis | 0.962963 | 0.995740 | 0.942029 | 0.993542 | 0.952381 | 0.997330 |
| 9.Origanum | 0.952941 | 0.998444 | 0.964286 | 0.996523 | 0.958580 | 0.997022 |
| 10.Satureja | 0.972222 | 0.998988 | 0.945946 | 0.998510 | 0.958904 | 0.999382 |
| 11.Pinus | 1.000000 | 1.000000 | 1.000000 | 1.000000 | 1.000000 | 1.000000 |
| 12.Calicotome | 0.939597 | 0.998391 | 0.979021 | 0.994039 | 0.958904 | 0.998848 |
| 13.Salvia | 1.000000 | 0.999480 | 0.988889 | 0.999503 | 0.994413 | 0.999988 |
| 14.Sinapis | 0.979798 | 0.995820 | 0.923810 | 0.995032 | 0.950980 | 0.999530 |
| 15.Ferula | 0.975610 | 1.000000 | 1.000000 | 0.999503 | 0.987654 | 0.999938 |
| 16.Asphodelus | 1.000000 | 0.999499 | 0.944444 | 0.999503 | 0.971429 | 1.000000 |
| 17.Oxalis | 1.000000 | 0.999485 | 0.985915 | 0.999503 | 0.992908 | 1.000000 |
| 18.Pistacia | 0.882353 | 1.000000 | 1.000000 | 0.999006 | 0.937500 | 0.999705 |
| 19.Ebenus | 0.909091 | 1.000000 | 1.000000 | 0.999503 | 0.952381 | 0.998138 |
| 20.Olea | 0.972152 | 0.998146 | 0.992248 | 0.993045 | 0.982097 | 0.999432 |
