# Supplementary material for: Pollen Grain Classification Based on Ensemble Transfer Learning on the Cretan Pollen Dataset
Source: Plants (Basel). 2022 Mar 29;11(7):919. doi: 10.3390/plants11070919 (PMC9002917; doi:10.3390/plants11070919)
Supplement: Supplementary file 1 [file plants-11-00919-s001.zip › Supplementary-Images/tables-results-of-all-models/ens_x_r_hard_metrics.html]

|  | sensitivity | specificity | precision | accuracy | f1 | auc |
| --- | --- | --- | --- | --- | --- | --- |
| 1.Thymbra | 0.821918 | 0.998969 | 0.967742 | 0.992548 | 0.888889 | nan |
| 2.Erica | 0.989011 | 0.999480 | 0.989011 | 0.999006 | 0.989011 | nan |
| 3.Castanea | 1.000000 | 0.998950 | 0.981982 | 0.999006 | 0.990909 | nan |
| 4.Eucalyptus | 0.835294 | 0.998963 | 0.972603 | 0.992052 | 0.898734 | nan |
| 5.Myrtus | 0.992366 | 1.000000 | 1.000000 | 0.998510 | 0.996169 | nan |
| 6.Ceratonia | 0.940000 | 0.992868 | 0.770492 | 0.991555 | 0.846847 | nan |
| 7.Urginea | 1.000000 | 1.000000 | 1.000000 | 1.000000 | 1.000000 | nan |
| 8.Vitis | 0.985185 | 0.994675 | 0.930070 | 0.994039 | 0.956835 | nan |
| 9.Origanum | 0.941176 | 0.996369 | 0.919540 | 0.994039 | 0.930233 | nan |
| 10.Satureja | 0.972222 | 0.997977 | 0.897436 | 0.997516 | 0.933333 | nan |
| 11.Pinus | 0.928571 | 1.000000 | 1.000000 | 0.999503 | 0.962963 | nan |
| 12.Calicotome | 0.932886 | 0.998391 | 0.978873 | 0.993542 | 0.955326 | nan |
| 13.Salvia | 1.000000 | 0.998960 | 0.978022 | 0.999006 | 0.988889 | nan |
| 14.Sinapis | 0.959596 | 0.996865 | 0.940594 | 0.995032 | 0.950000 | nan |
| 15.Ferula | 0.975610 | 0.999493 | 0.975610 | 0.999006 | 0.975610 | nan |
| 16.Asphodelus | 1.000000 | 0.999499 | 0.944444 | 0.999503 | 0.971429 | nan |
| 17.Oxalis | 1.000000 | 0.999485 | 0.985915 | 0.999503 | 0.992908 | nan |
| 18.Pistacia | 0.882353 | 1.000000 | 1.000000 | 0.999006 | 0.937500 | nan |
| 19.Ebenus | 0.909091 | 1.000000 | 1.000000 | 0.999503 | 0.952381 | nan |
| 20.Olea | 0.987342 | 0.993820 | 0.975000 | 0.992548 | 0.981132 | nan |
