# Supplementary material for: Pollen Grain Classification Based on Ensemble Transfer Learning on the Cretan Pollen Dataset
Source: Plants (Basel). 2022 Mar 29;11(7):919. doi: 10.3390/plants11070919 (PMC9002917; doi:10.3390/plants11070919)
Supplement: Supplementary file 1 [file plants-11-00919-s001.zip › Supplementary-Images/tables-results-of-all-models/ens_x_ir_i_hard_metrics.html]

|  | sensitivity | specificity | precision | accuracy | f1 | auc |
| --- | --- | --- | --- | --- | --- | --- |
| 1.Thymbra | 0.904110 | 0.998454 | 0.956522 | 0.995032 | 0.929577 | nan |
| 2.Erica | 1.000000 | 0.998959 | 0.978495 | 0.999006 | 0.989130 | nan |
| 3.Castanea | 1.000000 | 0.999475 | 0.990909 | 0.999503 | 0.995434 | nan |
| 4.Eucalyptus | 0.905882 | 0.998963 | 0.974684 | 0.995032 | 0.939024 | nan |
| 5.Myrtus | 0.989822 | 0.999383 | 0.997436 | 0.997516 | 0.993614 | nan |
| 6.Ceratonia | 0.980000 | 0.996943 | 0.890909 | 0.996523 | 0.933333 | nan |
| 7.Urginea | 1.000000 | 1.000000 | 1.000000 | 1.000000 | 1.000000 | nan |
| 8.Vitis | 0.970370 | 0.994143 | 0.922535 | 0.992548 | 0.945848 | nan |
| 9.Origanum | 0.941176 | 0.998444 | 0.963855 | 0.996026 | 0.952381 | nan |
| 10.Satureja | 0.972222 | 0.998988 | 0.945946 | 0.998510 | 0.958904 | nan |
| 11.Pinus | 1.000000 | 1.000000 | 1.000000 | 1.000000 | 1.000000 | nan |
| 12.Calicotome | 0.946309 | 0.998391 | 0.979167 | 0.994536 | 0.962457 | nan |
| 13.Salvia | 0.988764 | 1.000000 | 1.000000 | 0.999503 | 0.994350 | nan |
| 14.Sinapis | 0.989899 | 0.991118 | 0.852174 | 0.991058 | 0.915888 | nan |
| 15.Ferula | 0.975610 | 1.000000 | 1.000000 | 0.999503 | 0.987654 | nan |
| 16.Asphodelus | 1.000000 | 1.000000 | 1.000000 | 1.000000 | 1.000000 | nan |
| 17.Oxalis | 1.000000 | 0.999485 | 0.985915 | 0.999503 | 0.992908 | nan |
| 18.Pistacia | 0.941176 | 1.000000 | 1.000000 | 0.999503 | 0.969697 | nan |
| 19.Ebenus | 0.909091 | 1.000000 | 1.000000 | 0.999503 | 0.952381 | nan |
| 20.Olea | 0.967089 | 0.997528 | 0.989637 | 0.991555 | 0.978233 | nan |
