# Supplementary material for: Pollen Grain Classification Based on Ensemble Transfer Learning on the Cretan Pollen Dataset
Source: Plants (Basel). 2022 Mar 29;11(7):919. doi: 10.3390/plants11070919 (PMC9002917; doi:10.3390/plants11070919)
Supplement: Supplementary file 1 [file plants-11-00919-s001.zip › Supplementary-Images/tables-results-of-all-models/ens_x_ir_r_soft_metrics.html]

|  | sensitivity | specificity | precision | accuracy | f1 | auc |
| --- | --- | --- | --- | --- | --- | --- |
| 1.Thymbra | 0.904110 | 0.999485 | 0.985075 | 0.996026 | 0.942857 | 0.998913 |
| 2.Erica | 1.000000 | 0.998439 | 0.968085 | 0.998510 | 0.983784 | 1.000000 |
| 3.Castanea | 1.000000 | 0.998424 | 0.973214 | 0.998510 | 0.986425 | 1.000000 |
| 4.Eucalyptus | 0.929412 | 0.998444 | 0.963415 | 0.995529 | 0.946108 | 0.999378 |
| 5.Myrtus | 0.987277 | 1.000000 | 1.000000 | 0.997516 | 0.993598 | 0.999989 |
| 6.Ceratonia | 0.960000 | 0.993887 | 0.800000 | 0.993045 | 0.872727 | 0.998553 |
| 7.Urginea | 1.000000 | 1.000000 | 1.000000 | 1.000000 | 1.000000 | 1.000000 |
| 8.Vitis | 0.940741 | 0.994675 | 0.927007 | 0.991058 | 0.933824 | 0.998525 |
| 9.Origanum | 0.952941 | 0.998963 | 0.975904 | 0.997019 | 0.964286 | 0.996497 |
| 10.Satureja | 0.972222 | 0.999494 | 0.972222 | 0.999006 | 0.972222 | 0.999888 |
| 11.Pinus | 1.000000 | 1.000000 | 1.000000 | 1.000000 | 1.000000 | 1.000000 |
| 12.Calicotome | 0.953020 | 0.997854 | 0.972603 | 0.994536 | 0.962712 | 0.999600 |
| 13.Salvia | 1.000000 | 1.000000 | 1.000000 | 1.000000 | 1.000000 | 1.000000 |
| 14.Sinapis | 1.000000 | 0.991641 | 0.860870 | 0.992052 | 0.925234 | 0.999631 |
| 15.Ferula | 0.975610 | 1.000000 | 1.000000 | 0.999503 | 0.987654 | 0.999963 |
| 16.Asphodelus | 1.000000 | 1.000000 | 1.000000 | 1.000000 | 1.000000 | 1.000000 |
| 17.Oxalis | 1.000000 | 0.999485 | 0.985915 | 0.999503 | 0.992908 | 1.000000 |
| 18.Pistacia | 0.882353 | 1.000000 | 1.000000 | 0.999006 | 0.937500 | 0.999617 |
| 19.Ebenus | 0.909091 | 1.000000 | 1.000000 | 0.999503 | 0.952381 | 0.999319 |
| 20.Olea | 0.964557 | 0.998764 | 0.994778 | 0.992052 | 0.979434 | 0.999404 |
