# Supplementary material for: Pollen Grain Classification Based on Ensemble Transfer Learning on the Cretan Pollen Dataset
Source: Plants (Basel). 2022 Mar 29;11(7):919. doi: 10.3390/plants11070919 (PMC9002917; doi:10.3390/plants11070919)
Supplement: Supplementary file 1 [file plants-11-00919-s001.zip › Supplementary-Images/tables-results-of-all-models/ens_x_ir_hard_metrics.html]

|  | sensitivity | specificity | precision | accuracy | f1 | auc |
| --- | --- | --- | --- | --- | --- | --- |
| 1.Thymbra | 0.821918 | 1.000000 | 1.000000 | 0.993542 | 0.902256 | nan |
| 2.Erica | 0.989011 | 1.000000 | 1.000000 | 0.999503 | 0.994475 | nan |
| 3.Castanea | 1.000000 | 1.000000 | 1.000000 | 1.000000 | 1.000000 | nan |
| 4.Eucalyptus | 0.788235 | 0.998963 | 0.971014 | 0.990065 | 0.870130 | nan |
| 5.Myrtus | 0.982188 | 0.999383 | 0.997416 | 0.996026 | 0.989744 | nan |
| 6.Ceratonia | 0.940000 | 0.993377 | 0.783333 | 0.992052 | 0.854545 | nan |
| 7.Urginea | 1.000000 | 0.999490 | 0.981818 | 0.999503 | 0.990826 | nan |
| 8.Vitis | 0.948148 | 0.992545 | 0.901408 | 0.989568 | 0.924188 | nan |
| 9.Origanum | 0.941176 | 0.998963 | 0.975610 | 0.996523 | 0.958084 | nan |
| 10.Satureja | 1.000000 | 0.997977 | 0.900000 | 0.998013 | 0.947368 | nan |
| 11.Pinus | 0.928571 | 1.000000 | 1.000000 | 0.999503 | 0.962963 | nan |
| 12.Calicotome | 0.932886 | 0.997318 | 0.965278 | 0.992548 | 0.948805 | nan |
| 13.Salvia | 0.988764 | 0.998441 | 0.967033 | 0.998013 | 0.977778 | nan |
| 14.Sinapis | 1.000000 | 0.989028 | 0.825000 | 0.989568 | 0.904110 | nan |
| 15.Ferula | 0.975610 | 0.999493 | 0.975610 | 0.999006 | 0.975610 | nan |
| 16.Asphodelus | 1.000000 | 1.000000 | 1.000000 | 1.000000 | 1.000000 | nan |
| 17.Oxalis | 1.000000 | 0.998456 | 0.958904 | 0.998510 | 0.979021 | nan |
| 18.Pistacia | 0.882353 | 1.000000 | 1.000000 | 0.999006 | 0.937500 | nan |
| 19.Ebenus | 0.909091 | 1.000000 | 1.000000 | 0.999503 | 0.952381 | nan |
| 20.Olea | 0.969620 | 0.992583 | 0.969620 | 0.988077 | 0.969620 | nan |
