# Supplementary material for: Pollen Grain Classification Based on Ensemble Transfer Learning on the Cretan Pollen Dataset
Source: Plants (Basel). 2022 Mar 29;11(7):919. doi: 10.3390/plants11070919 (PMC9002917; doi:10.3390/plants11070919)
Supplement: Supplementary file 1 [file plants-11-00919-s001.zip › Supplementary-Images/tables-results-of-all-models/ens_ir_r_soft_metrics.html]

|  | sensitivity | specificity | precision | accuracy | f1 | auc |
| --- | --- | --- | --- | --- | --- | --- |
| 1.Thymbra | 0.917808 | 1.000000 | 1.000000 | 0.997019 | 0.957143 | 0.998757 |
| 2.Erica | 1.000000 | 0.998439 | 0.968085 | 0.998510 | 0.983784 | 0.999977 |
| 3.Castanea | 1.000000 | 0.998950 | 0.981982 | 0.999006 | 0.990909 | 1.000000 |
| 4.Eucalyptus | 0.941176 | 0.998444 | 0.963855 | 0.996026 | 0.952381 | 0.999469 |
| 5.Myrtus | 0.989822 | 0.999383 | 0.997436 | 0.997516 | 0.993614 | 0.999909 |
| 6.Ceratonia | 0.940000 | 0.992359 | 0.758065 | 0.991058 | 0.839286 | 0.998563 |
| 7.Urginea | 1.000000 | 1.000000 | 1.000000 | 1.000000 | 1.000000 | 1.000000 |
| 8.Vitis | 0.918519 | 0.995208 | 0.932331 | 0.990065 | 0.925373 | 0.998229 |
| 9.Origanum | 0.952941 | 0.998963 | 0.975904 | 0.997019 | 0.964286 | 0.997016 |
| 10.Satureja | 0.944444 | 0.999494 | 0.971429 | 0.998510 | 0.957746 | 0.999902 |
| 11.Pinus | 1.000000 | 1.000000 | 1.000000 | 1.000000 | 1.000000 | 1.000000 |
| 12.Calicotome | 0.953020 | 0.997854 | 0.972603 | 0.994536 | 0.962712 | 0.998362 |
| 13.Salvia | 1.000000 | 1.000000 | 1.000000 | 1.000000 | 1.000000 | 1.000000 |
| 14.Sinapis | 0.989899 | 0.988506 | 0.816667 | 0.988574 | 0.894977 | 0.999340 |
| 15.Ferula | 0.975610 | 1.000000 | 1.000000 | 0.999503 | 0.987654 | 0.999963 |
| 16.Asphodelus | 1.000000 | 1.000000 | 1.000000 | 1.000000 | 1.000000 | 1.000000 |
| 17.Oxalis | 1.000000 | 0.998971 | 0.972222 | 0.999006 | 0.985915 | 0.999934 |
| 18.Pistacia | 0.882353 | 1.000000 | 1.000000 | 0.999006 | 0.937500 | 0.999764 |
| 19.Ebenus | 0.909091 | 1.000000 | 1.000000 | 0.999503 | 0.952381 | 0.999319 |
| 20.Olea | 0.949367 | 0.998146 | 0.992063 | 0.988574 | 0.970246 | 0.998651 |
