# Supplementary material for: Pollen Grain Classification Based on Ensemble Transfer Learning on the Cretan Pollen Dataset
Source: Plants (Basel). 2022 Mar 29;11(7):919. doi: 10.3390/plants11070919 (PMC9002917; doi:10.3390/plants11070919)
Supplement: Supplementary file 1 [file plants-11-00919-s001.zip › Supplementary-Images/tables-results-of-all-models/inception_resnet_metrics.html]

|  | sensitivity | specificity | precision | accuracy | f1 | auc |
| --- | --- | --- | --- | --- | --- | --- |
| 1.Thymbra | 0.849315 | 1.000000 | 1.000000 | 0.994536 | 0.918519 | 0.993913 |
| 2.Erica | 0.989011 | 0.998959 | 0.978261 | 0.998510 | 0.983607 | 0.999943 |
| 3.Castanea | 1.000000 | 0.999475 | 0.990909 | 0.999503 | 0.995434 | 1.000000 |
| 4.Eucalyptus | 0.858824 | 0.997925 | 0.948052 | 0.992052 | 0.901235 | 0.997486 |
| 5.Myrtus | 0.979644 | 0.999383 | 0.997409 | 0.995529 | 0.988447 | 0.999717 |
| 6.Ceratonia | 0.960000 | 0.990830 | 0.727273 | 0.990065 | 0.827586 | 0.995466 |
| 7.Urginea | 1.000000 | 0.999490 | 0.981818 | 0.999503 | 0.990826 | 1.000000 |
| 8.Vitis | 0.888889 | 0.994143 | 0.916031 | 0.987084 | 0.902256 | 0.995803 |
| 9.Origanum | 0.952941 | 0.998963 | 0.975904 | 0.997019 | 0.964286 | 0.996534 |
| 10.Satureja | 0.944444 | 0.998988 | 0.944444 | 0.998013 | 0.944444 | 0.998623 |
| 11.Pinus | 1.000000 | 1.000000 | 1.000000 | 1.000000 | 1.000000 | 1.000000 |
| 12.Calicotome | 0.939597 | 0.997854 | 0.972222 | 0.993542 | 0.955631 | 0.998092 |
| 13.Salvia | 0.988764 | 0.999480 | 0.988764 | 0.999006 | 0.988764 | 0.998902 |
| 14.Sinapis | 1.000000 | 0.981191 | 0.733333 | 0.982116 | 0.846154 | 0.998839 |
| 15.Ferula | 0.975610 | 0.999493 | 0.975610 | 0.999006 | 0.975610 | 0.999951 |
| 16.Asphodelus | 1.000000 | 1.000000 | 1.000000 | 1.000000 | 1.000000 | 1.000000 |
| 17.Oxalis | 1.000000 | 0.998456 | 0.958904 | 0.998510 | 0.979021 | 0.999978 |
| 18.Pistacia | 0.882353 | 1.000000 | 1.000000 | 0.999006 | 0.937500 | 0.997053 |
| 19.Ebenus | 0.909091 | 1.000000 | 1.000000 | 0.999503 | 0.952381 | 0.999637 |
| 20.Olea | 0.931646 | 0.994438 | 0.976127 | 0.982116 | 0.953368 | 0.994046 |
