# Supplementary material for: Pollen Grain Classification Based on Ensemble Transfer Learning on the Cretan Pollen Dataset
Source: Plants (Basel). 2022 Mar 29;11(7):919. doi: 10.3390/plants11070919 (PMC9002917; doi:10.3390/plants11070919)
Supplement: Supplementary file 1 [file plants-11-00919-s001.zip › Supplementary-Images/tables-results-of-all-models/resnet_metrics.html]

|  | sensitivity | specificity | precision | accuracy | f1 | auc |
| --- | --- | --- | --- | --- | --- | --- |
| 1.Thymbra | 0.863014 | 0.997938 | 0.940299 | 0.993045 | 0.900000 | 0.997522 |
| 2.Erica | 0.989011 | 0.998959 | 0.978261 | 0.998510 | 0.983607 | 0.999451 |
| 3.Castanea | 1.000000 | 0.998424 | 0.973214 | 0.998510 | 0.986425 | 1.000000 |
| 4.Eucalyptus | 0.905882 | 0.995332 | 0.895349 | 0.991555 | 0.900585 | 0.996540 |
| 5.Myrtus | 0.992366 | 0.999383 | 0.997442 | 0.998013 | 0.994898 | 0.999640 |
| 6.Ceratonia | 0.940000 | 0.986755 | 0.643836 | 0.985594 | 0.764228 | 0.995405 |
| 7.Urginea | 1.000000 | 1.000000 | 1.000000 | 1.000000 | 1.000000 | 1.000000 |
| 8.Vitis | 0.888889 | 0.996273 | 0.944882 | 0.989071 | 0.916031 | 0.994174 |
| 9.Origanum | 0.952941 | 0.996888 | 0.931034 | 0.995032 | 0.941860 | 0.997529 |
| 10.Satureja | 0.972222 | 0.998988 | 0.945946 | 0.998510 | 0.958904 | 0.999649 |
| 11.Pinus | 1.000000 | 1.000000 | 1.000000 | 1.000000 | 1.000000 | 1.000000 |
| 12.Calicotome | 0.939597 | 0.997318 | 0.965517 | 0.993045 | 0.952381 | 0.996497 |
| 13.Salvia | 1.000000 | 1.000000 | 1.000000 | 1.000000 | 1.000000 | 1.000000 |
| 14.Sinapis | 0.949495 | 0.994775 | 0.903846 | 0.992548 | 0.926108 | 0.997593 |
| 15.Ferula | 0.975610 | 0.999493 | 0.975610 | 0.999006 | 0.975610 | 0.999938 |
| 16.Asphodelus | 1.000000 | 0.999499 | 0.944444 | 0.999503 | 0.971429 | 1.000000 |
| 17.Oxalis | 1.000000 | 0.999485 | 0.985915 | 0.999503 | 0.992908 | 0.999912 |
| 18.Pistacia | 0.882353 | 1.000000 | 1.000000 | 0.999006 | 0.937500 | 0.999764 |
| 19.Ebenus | 0.909091 | 1.000000 | 1.000000 | 0.999503 | 0.952381 | 0.995686 |
| 20.Olea | 0.946835 | 0.996292 | 0.984211 | 0.986587 | 0.965161 | 0.997894 |
