# Supplementary material for: Pollen Grain Classification Based on Ensemble Transfer Learning on the Cretan Pollen Dataset
Source: Plants (Basel). 2022 Mar 29;11(7):919. doi: 10.3390/plants11070919 (PMC9002917; doi:10.3390/plants11070919)
Supplement: Supplementary file 1 [file plants-11-00919-s001.zip › Supplementary-Images/tables-results-of-all-models/inception_metrics.html]

|  | sensitivity | specificity | precision | accuracy | f1 | auc |
| --- | --- | --- | --- | --- | --- | --- |
| 1.Thymbra | 0.931507 | 0.995361 | 0.883117 | 0.993045 | 0.906667 | 0.994549 |
| 2.Erica | 1.000000 | 0.998439 | 0.968085 | 0.998510 | 0.983784 | 0.999994 |
| 3.Castanea | 1.000000 | 0.995798 | 0.931624 | 0.996026 | 0.964602 | 0.999986 |
| 4.Eucalyptus | 0.894118 | 0.998963 | 0.974359 | 0.994536 | 0.932515 | 0.998835 |
| 5.Myrtus | 0.979644 | 0.999383 | 0.997409 | 0.995529 | 0.988447 | 0.999827 |
| 6.Ceratonia | 0.940000 | 0.995925 | 0.854545 | 0.994536 | 0.895238 | 0.998304 |
| 7.Urginea | 1.000000 | 1.000000 | 1.000000 | 1.000000 | 1.000000 | 1.000000 |
| 8.Vitis | 0.955556 | 0.994675 | 0.928058 | 0.992052 | 0.941606 | 0.998848 |
| 9.Origanum | 0.929412 | 0.997925 | 0.951807 | 0.995032 | 0.940476 | 0.993825 |
| 10.Satureja | 0.944444 | 0.998988 | 0.944444 | 0.998013 | 0.944444 | 0.997794 |
| 11.Pinus | 1.000000 | 1.000000 | 1.000000 | 1.000000 | 1.000000 | 1.000000 |
| 12.Calicotome | 0.932886 | 0.999464 | 0.992857 | 0.994536 | 0.961938 | 0.995831 |
| 13.Salvia | 1.000000 | 1.000000 | 1.000000 | 1.000000 | 1.000000 | 1.000000 |
| 14.Sinapis | 0.959596 | 0.993208 | 0.879630 | 0.991555 | 0.917874 | 0.998274 |
| 15.Ferula | 0.951220 | 1.000000 | 1.000000 | 0.999006 | 0.975000 | 1.000000 |
| 16.Asphodelus | 1.000000 | 0.999499 | 0.944444 | 0.999503 | 0.971429 | 1.000000 |
| 17.Oxalis | 1.000000 | 0.999485 | 0.985915 | 0.999503 | 0.992908 | 0.999949 |
| 18.Pistacia | 0.941176 | 1.000000 | 1.000000 | 0.999503 | 0.969697 | 0.999971 |
| 19.Ebenus | 0.909091 | 1.000000 | 1.000000 | 0.999503 | 0.952381 | 0.998229 |
| 20.Olea | 0.964557 | 0.995056 | 0.979434 | 0.989071 | 0.971939 | 0.998438 |
