# Supplementary material for: Pollen Grain Classification Based on Ensemble Transfer Learning on the Cretan Pollen Dataset
Source: Plants (Basel). 2022 Mar 29;11(7):919. doi: 10.3390/plants11070919 (PMC9002917; doi:10.3390/plants11070919)
Supplement: Supplementary file 1 [file plants-11-00919-s001.zip › Supplementary-Images/tables-results-of-all-models/ens_x_i_hard_metrics.html]

|  | sensitivity | specificity | precision | accuracy | f1 | auc |
| --- | --- | --- | --- | --- | --- | --- |
| 1.Thymbra | 0.863014 | 0.998454 | 0.954545 | 0.993542 | 0.906475 | nan |
| 2.Erica | 1.000000 | 0.999480 | 0.989130 | 0.999503 | 0.994536 | nan |
| 3.Castanea | 1.000000 | 0.999475 | 0.990909 | 0.999503 | 0.995434 | nan |
| 4.Eucalyptus | 0.811765 | 0.998444 | 0.958333 | 0.990561 | 0.878981 | nan |
| 5.Myrtus | 0.992366 | 0.999383 | 0.997442 | 0.998013 | 0.994898 | nan |
| 6.Ceratonia | 0.920000 | 0.994396 | 0.807018 | 0.992548 | 0.859813 | nan |
| 7.Urginea | 1.000000 | 1.000000 | 1.000000 | 1.000000 | 1.000000 | nan |
| 8.Vitis | 0.970370 | 0.993078 | 0.909722 | 0.991555 | 0.939068 | nan |
| 9.Origanum | 0.941176 | 0.997407 | 0.941176 | 0.995032 | 0.941176 | nan |
| 10.Satureja | 0.972222 | 0.997977 | 0.897436 | 0.997516 | 0.933333 | nan |
| 11.Pinus | 0.928571 | 1.000000 | 1.000000 | 0.999503 | 0.962963 | nan |
| 12.Calicotome | 0.932886 | 0.998927 | 0.985816 | 0.994039 | 0.958621 | nan |
| 13.Salvia | 1.000000 | 0.998960 | 0.978022 | 0.999006 | 0.988889 | nan |
| 14.Sinapis | 0.959596 | 0.994775 | 0.904762 | 0.993045 | 0.931373 | nan |
| 15.Ferula | 0.975610 | 1.000000 | 1.000000 | 0.999503 | 0.987654 | nan |
| 16.Asphodelus | 1.000000 | 0.999499 | 0.944444 | 0.999503 | 0.971429 | nan |
| 17.Oxalis | 1.000000 | 0.999485 | 0.985915 | 0.999503 | 0.992908 | nan |
| 18.Pistacia | 0.941176 | 1.000000 | 1.000000 | 0.999503 | 0.969697 | nan |
| 19.Ebenus | 0.909091 | 1.000000 | 1.000000 | 0.999503 | 0.952381 | nan |
| 20.Olea | 0.979747 | 0.993201 | 0.972362 | 0.990561 | 0.976040 | nan |
