# Supplementary material for: Pollen Grain Classification Based on Ensemble Transfer Learning on the Cretan Pollen Dataset
Source: Plants (Basel). 2022 Mar 29;11(7):919. doi: 10.3390/plants11070919 (PMC9002917; doi:10.3390/plants11070919)
Supplement: Supplementary file 1 [file plants-11-00919-s001.zip › Supplementary-Images/tables-results-of-all-models/ens_x_ir_soft_metrics.html]

|  | sensitivity | specificity | precision | accuracy | f1 | auc |
| --- | --- | --- | --- | --- | --- | --- |
| 1.Thymbra | 0.904110 | 1.000000 | 1.000000 | 0.996523 | 0.949640 | 0.995629 |
| 2.Erica | 1.000000 | 0.997919 | 0.957895 | 0.998013 | 0.978495 | 1.000000 |
| 3.Castanea | 1.000000 | 0.998950 | 0.981982 | 0.999006 | 0.990909 | 1.000000 |
| 4.Eucalyptus | 0.905882 | 0.997925 | 0.950617 | 0.994039 | 0.927711 | 0.999011 |
| 5.Myrtus | 0.989822 | 1.000000 | 1.000000 | 0.998013 | 0.994885 | 0.999876 |
| 6.Ceratonia | 0.960000 | 0.992868 | 0.774194 | 0.992052 | 0.857143 | 0.997850 |
| 7.Urginea | 1.000000 | 1.000000 | 1.000000 | 1.000000 | 1.000000 | 1.000000 |
| 8.Vitis | 0.955556 | 0.996273 | 0.948529 | 0.993542 | 0.952030 | 0.998698 |
| 9.Origanum | 0.941176 | 0.998963 | 0.975610 | 0.996523 | 0.958084 | 0.995619 |
| 10.Satureja | 1.000000 | 0.998988 | 0.947368 | 0.999006 | 0.972973 | 0.999859 |
| 11.Pinus | 0.928571 | 1.000000 | 1.000000 | 0.999503 | 0.962963 | 1.000000 |
| 12.Calicotome | 0.953020 | 0.997854 | 0.972603 | 0.994536 | 0.962712 | 0.998923 |
| 13.Salvia | 0.988764 | 0.999480 | 0.988764 | 0.999006 | 0.988764 | 0.999807 |
| 14.Sinapis | 1.000000 | 0.986938 | 0.798387 | 0.987581 | 0.887892 | 0.999388 |
| 15.Ferula | 0.975610 | 1.000000 | 1.000000 | 0.999503 | 0.987654 | 0.999951 |
| 16.Asphodelus | 1.000000 | 1.000000 | 1.000000 | 1.000000 | 1.000000 | 1.000000 |
| 17.Oxalis | 1.000000 | 0.999485 | 0.985915 | 0.999503 | 0.992908 | 1.000000 |
| 18.Pistacia | 0.882353 | 1.000000 | 1.000000 | 0.999006 | 0.937500 | 0.995373 |
| 19.Ebenus | 0.909091 | 1.000000 | 1.000000 | 0.999503 | 0.952381 | 0.999410 |
| 20.Olea | 0.944304 | 0.999382 | 0.997326 | 0.988574 | 0.970091 | 0.998442 |
