# Supplementary material for: Pollen Grain Classification Based on Ensemble Transfer Learning on the Cretan Pollen Dataset
Source: Plants (Basel). 2022 Mar 29;11(7):919. doi: 10.3390/plants11070919 (PMC9002917; doi:10.3390/plants11070919)
Supplement: Supplementary file 1 [file plants-11-00919-s001.zip › Supplementary-Images/roc-curves-of-all-models/thymbra_specific_roc.pdf]

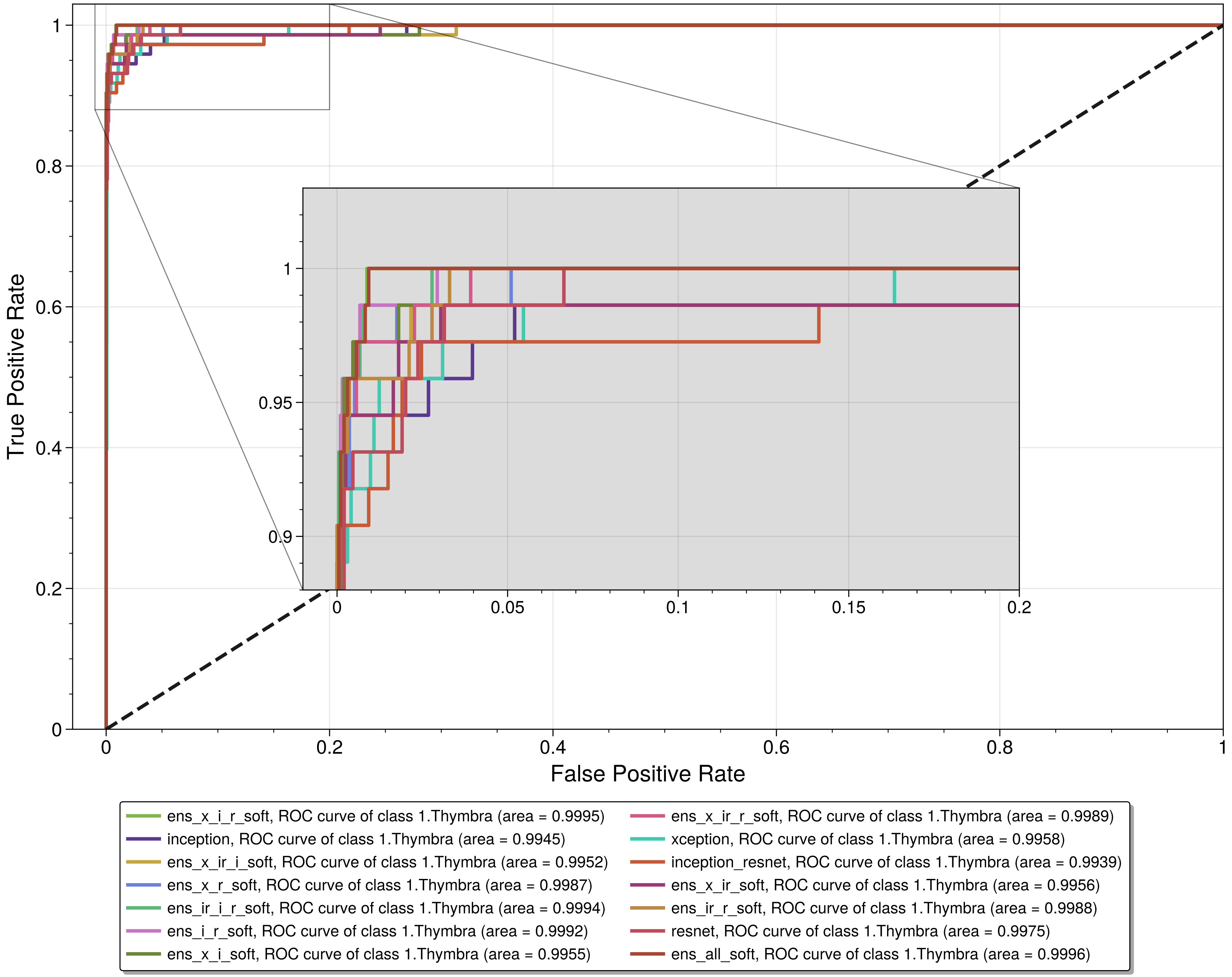

True Positive Rate
False Positive Rate
0
0.2
0.4
0.6
0.8
1
0.05
0.1
0.15
0.9
0.95
ens_x_i_r_soft, ROC curve of class 1.Thymbra (area = 0.9995)
inception, ROC curve of class 1.Thymbra (area = 0.9945)
ens_x_ir_i_soft, ROC curve of class 1.Thymbra (area = 0.9952)
ens_x_r_soft, ROC curve of class 1.Thymbra (area = 0.9987)
ens_ir_i_r_soft, ROC curve of class 1.Thymbra (area = 0.9994)
ens_i_r_soft, ROC curve of class 1.Thymbra (area = 0.9992)
ens_x_i_soft, ROC curve of class 1.Thymbra (area = 0.9955)
ens_x_ir_r_soft, ROC curve of class 1.Thymbra (area = 0.9989)
xception, ROC curve of class 1.Thymbra (area = 0.9958)
inception_resnet, ROC curve of class 1.Thymbra (area = 0.9939)
ens_x_ir_soft, ROC curve of class 1.Thymbra (area = 0.9956)
ens_ir_r_soft, ROC curve of class 1.Thymbra (area = 0.9988)
resnet, ROC curve of class 1.Thymbra (area = 0.9975)
ens_all_soft, ROC curve of class 1.Thymbra (area = 0.9996)
