# Supplementary figures and images for: Pollen Grain Classification Based on Ensemble Transfer Learning on the Cretan Pollen Dataset
Source: Plants (Basel). 2022 Mar 29;11(7):919. doi: 10.3390/plants11070919 (PMC9002917; doi:10.3390/plants11070919)

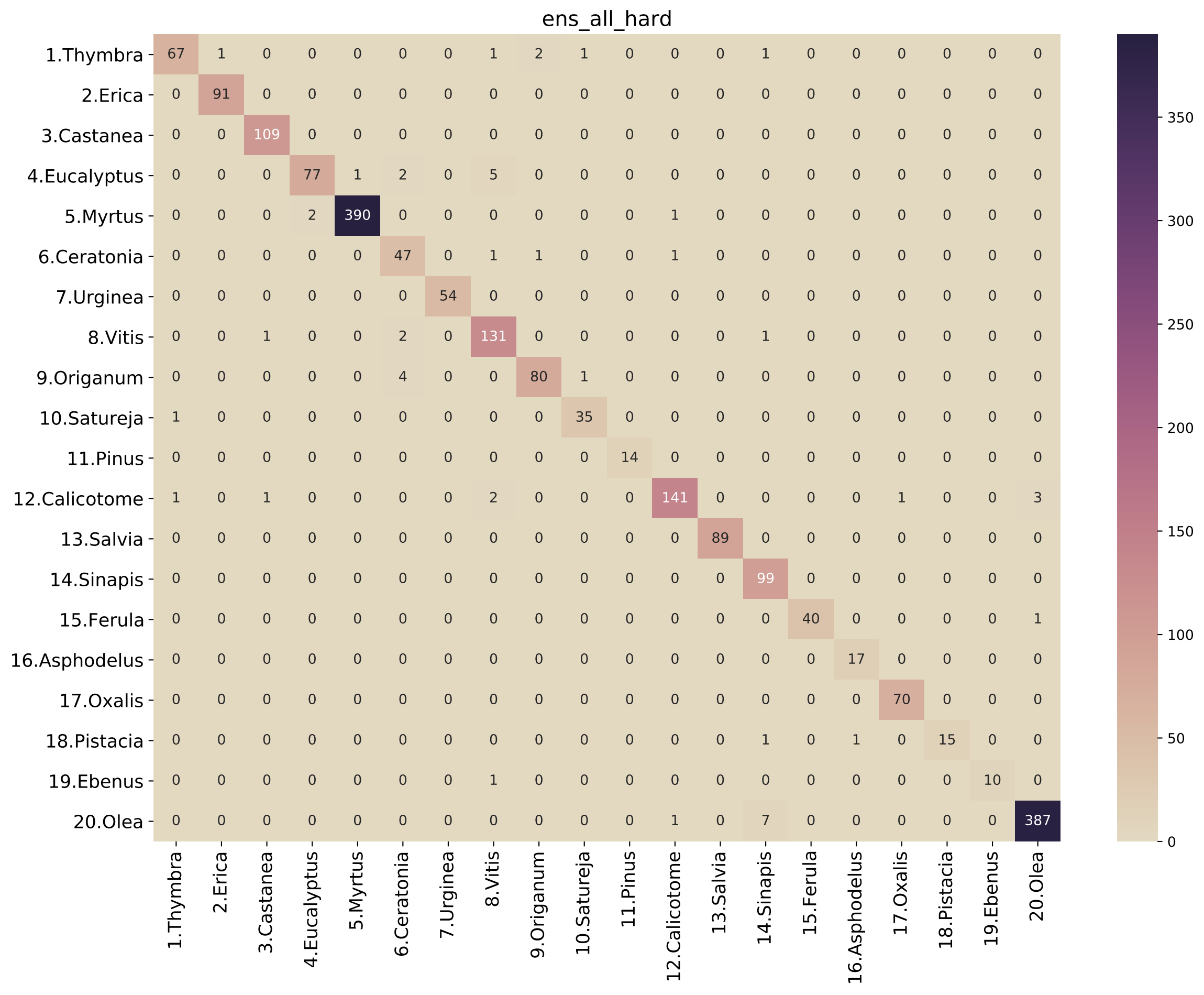

Supplement: Supplementary file 1 [file plants-11-00919-s001.zip › Supplementary-Images/confusion-matrices-of-all-models/ens_all_hard_cm.pdf]

ens\_all\_soft

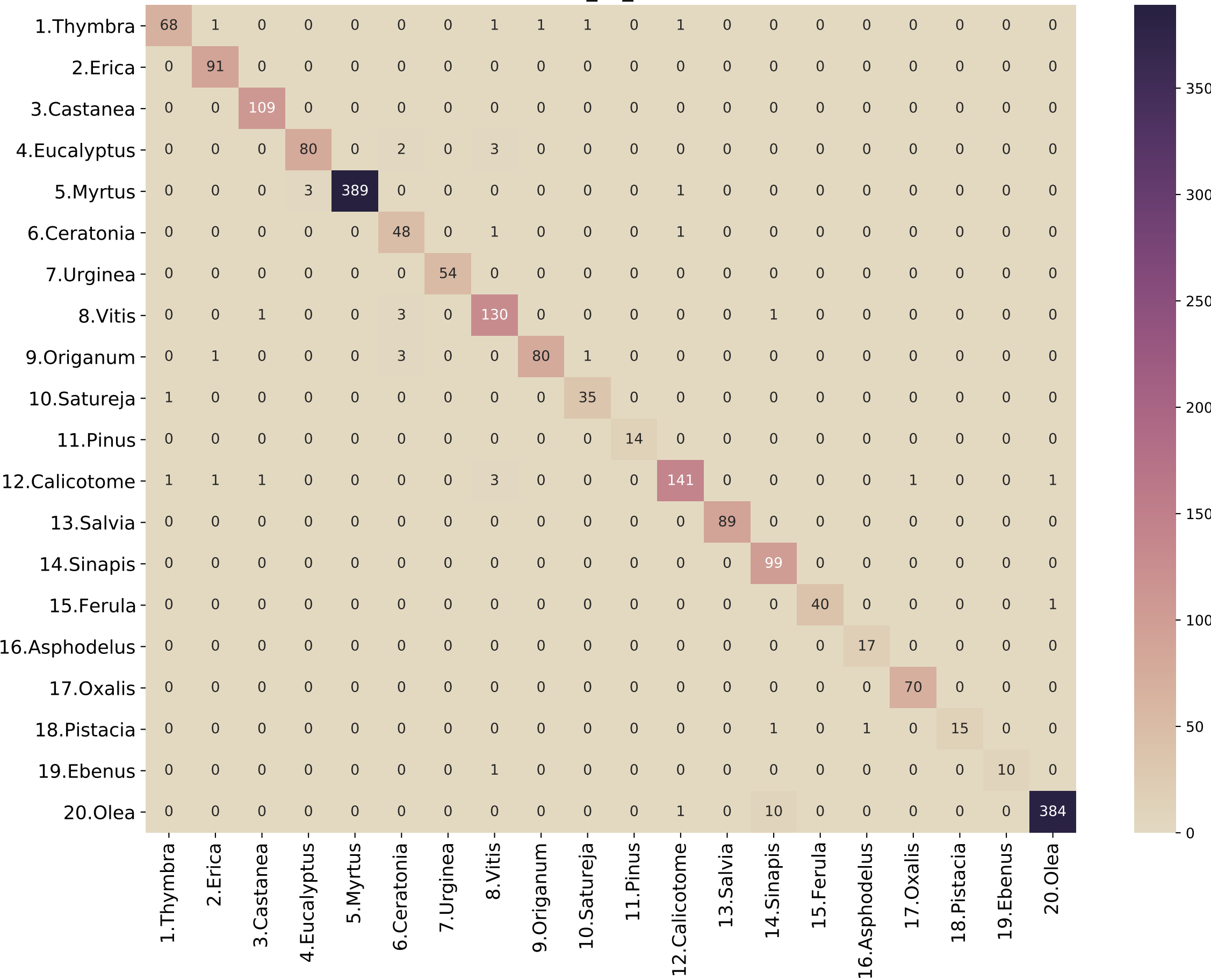

Supplement: Supplementary file 1 [file plants-11-00919-s001.zip › Supplementary-Images/confusion-matrices-of-all-models/ens_all_soft_cm.pdf]

inception\_resnet

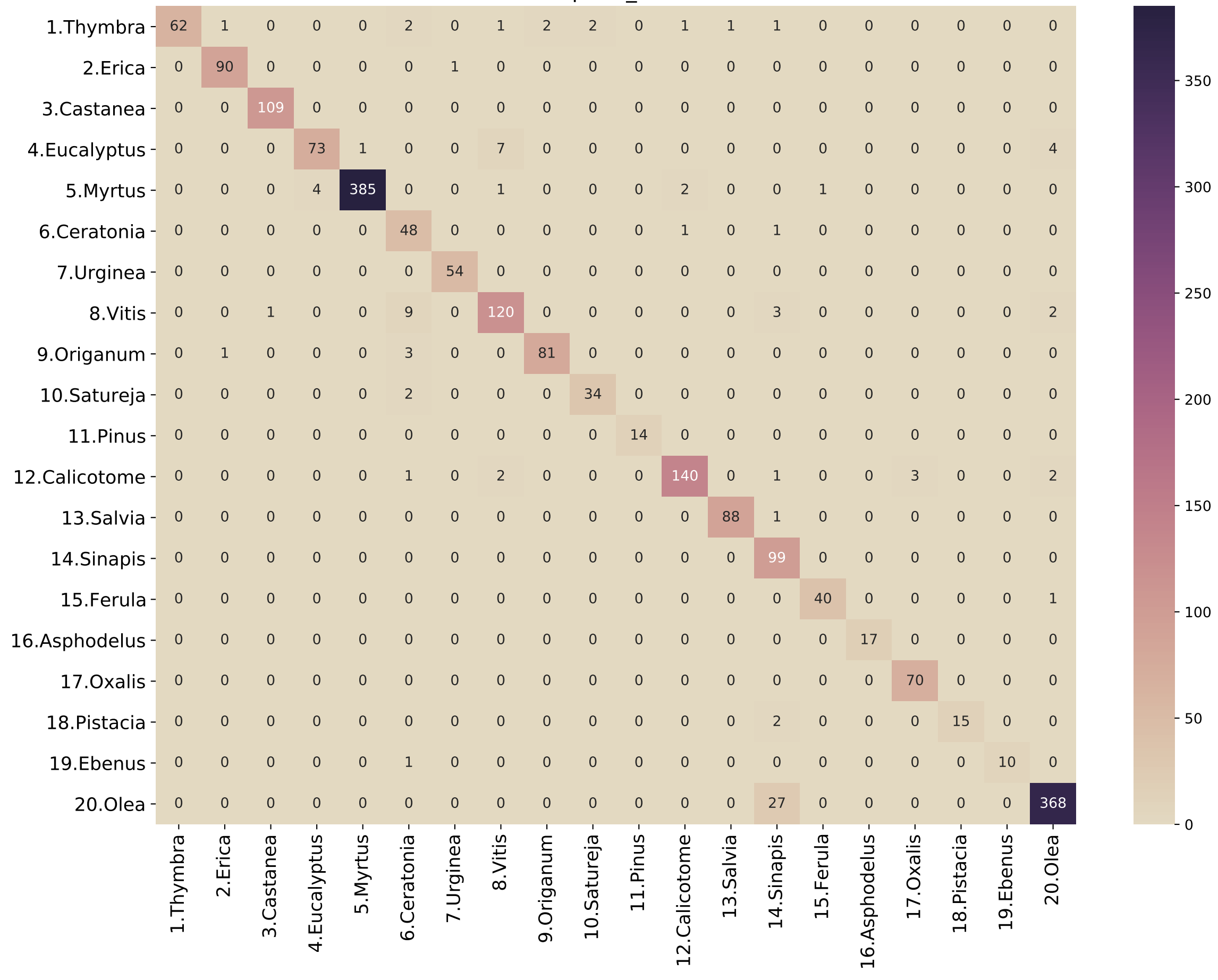

Supplement: Supplementary file 1 [file plants-11-00919-s001.zip › Supplementary-Images/confusion-matrices-of-all-models/inception_resnet_cm.pdf]

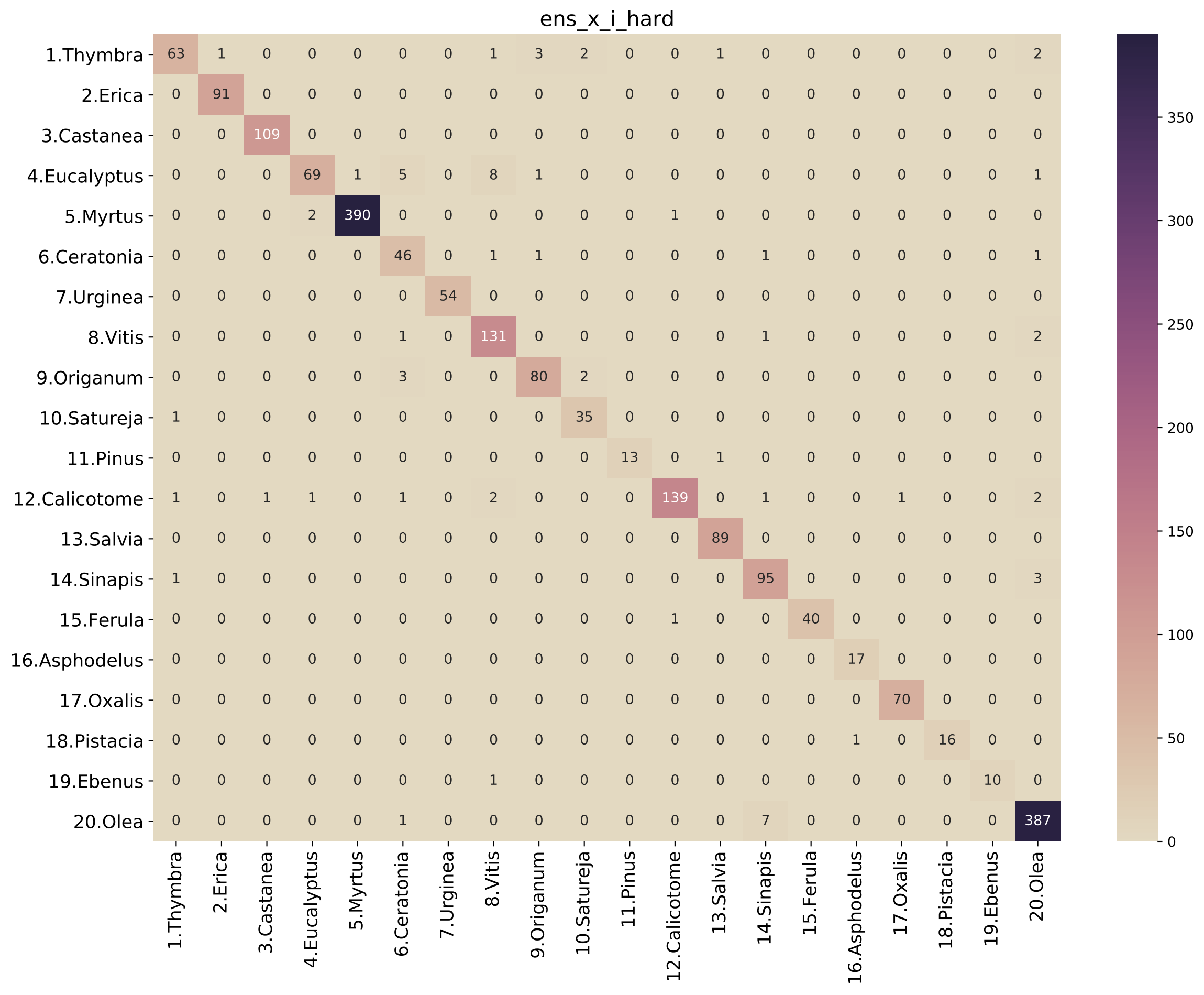

Supplement: Supplementary file 1 [file plants-11-00919-s001.zip › Supplementary-Images/confusion-matrices-of-all-models/ens_x_i_hard_cm.pdf]

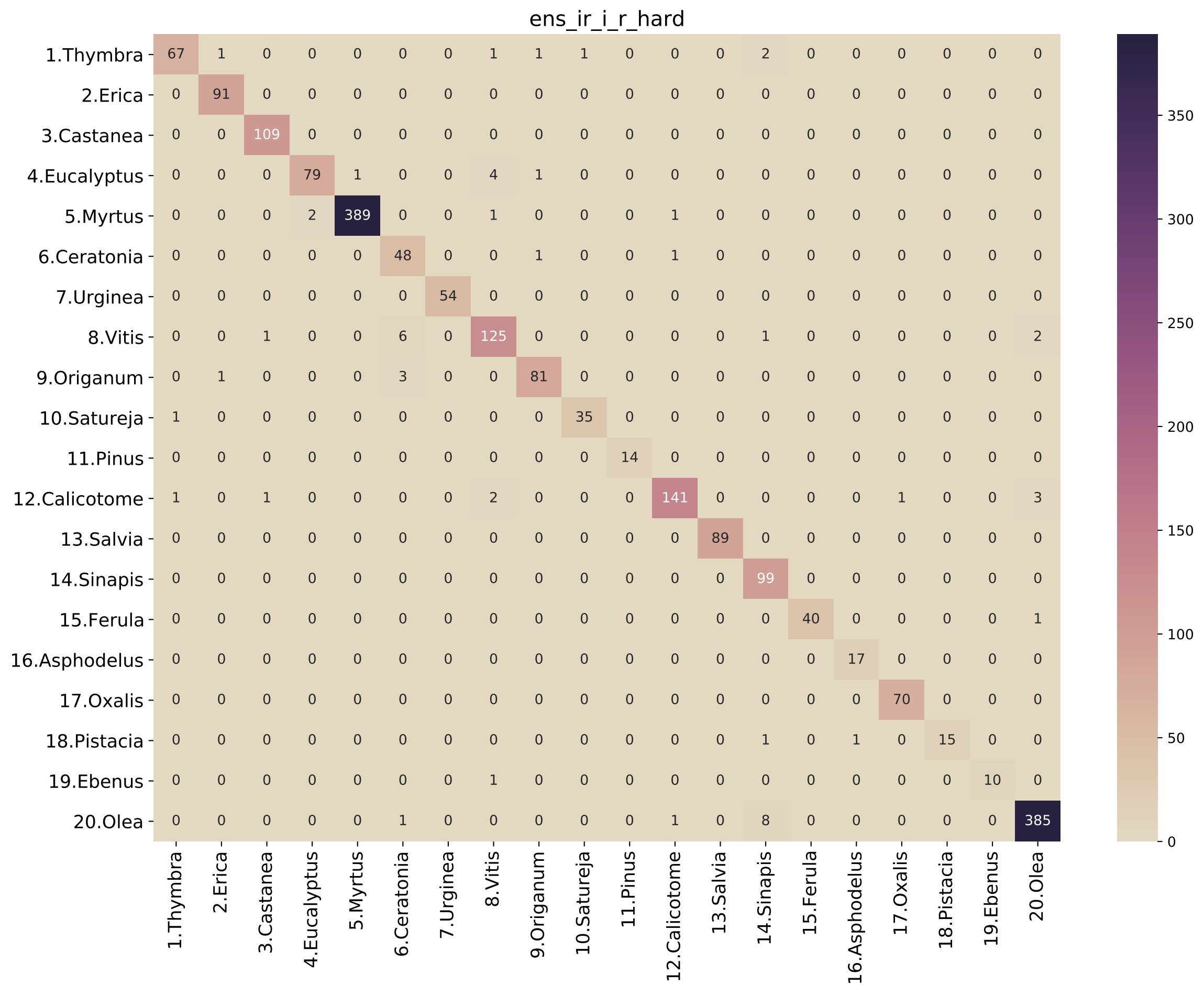

Supplement: Supplementary file 1 [file plants-11-00919-s001.zip › Supplementary-Images/confusion-matrices-of-all-models/ens_ir_i_r_hard_cm.pdf]

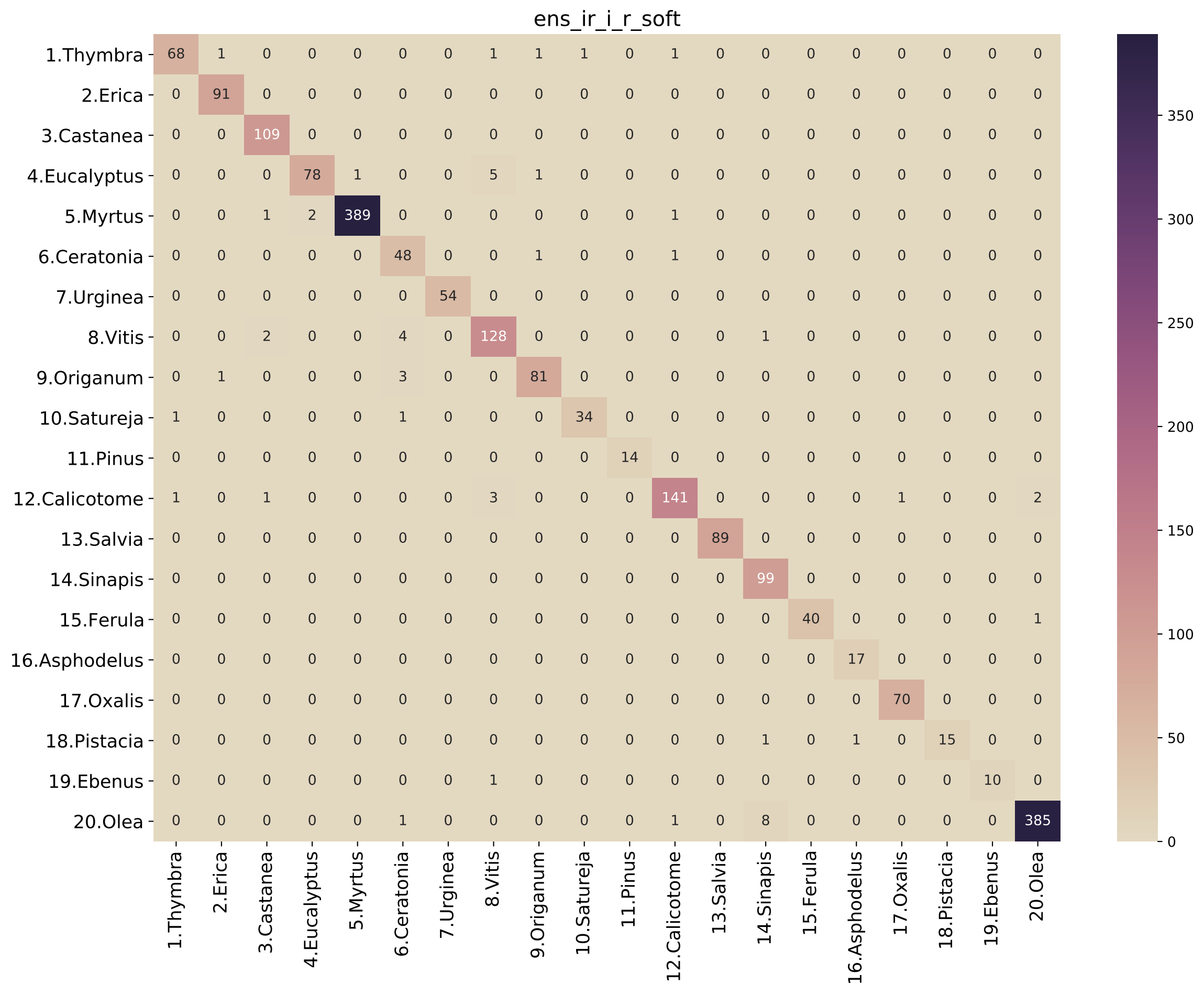

Supplement: Supplementary file 1 [file plants-11-00919-s001.zip › Supplementary-Images/confusion-matrices-of-all-models/ens_ir_i_r_soft_cm.pdf]

ens\_i\_r\_soft

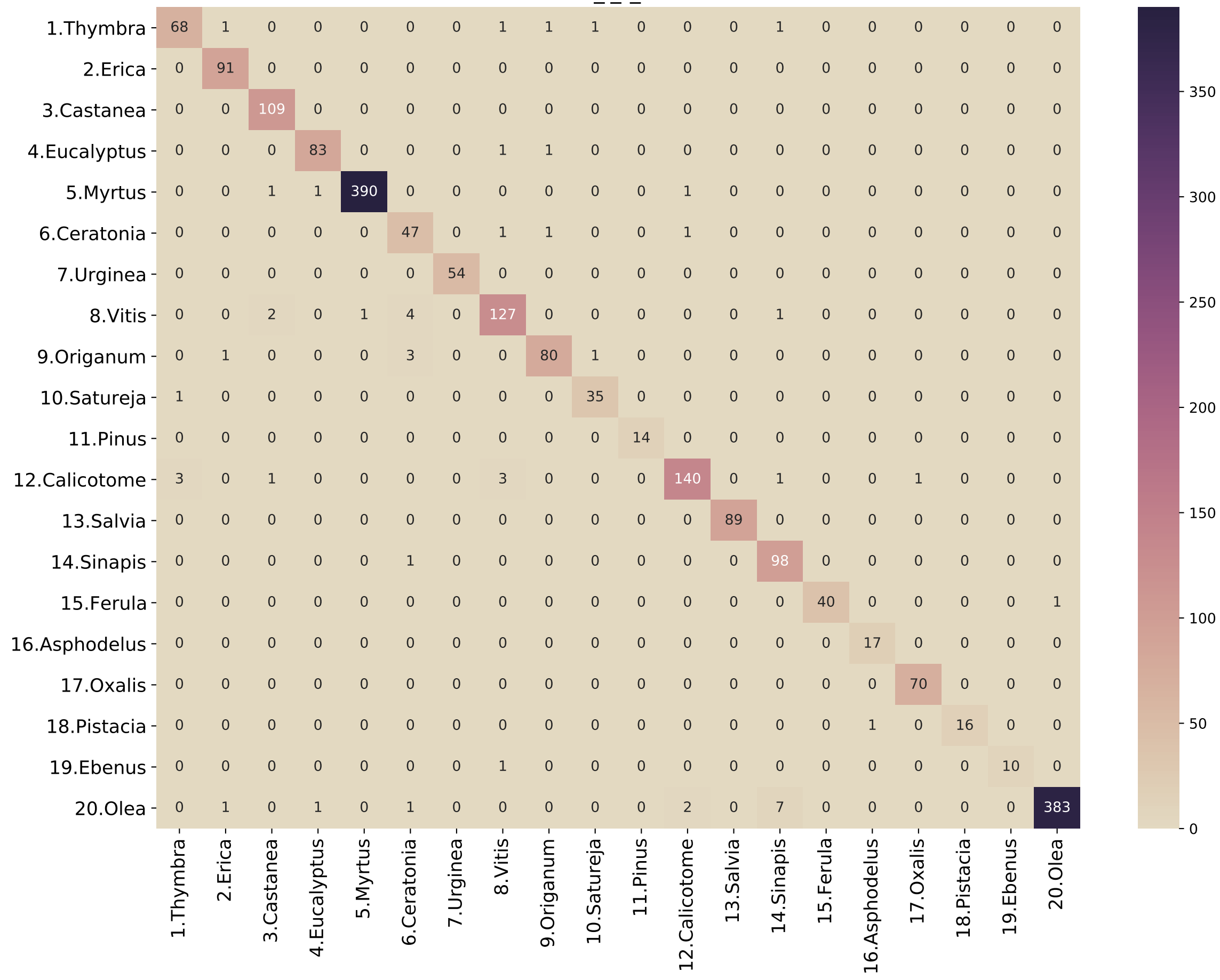

Supplement: Supplementary file 1 [file plants-11-00919-s001.zip › Supplementary-Images/confusion-matrices-of-all-models/ens_i_r_soft_cm.pdf]

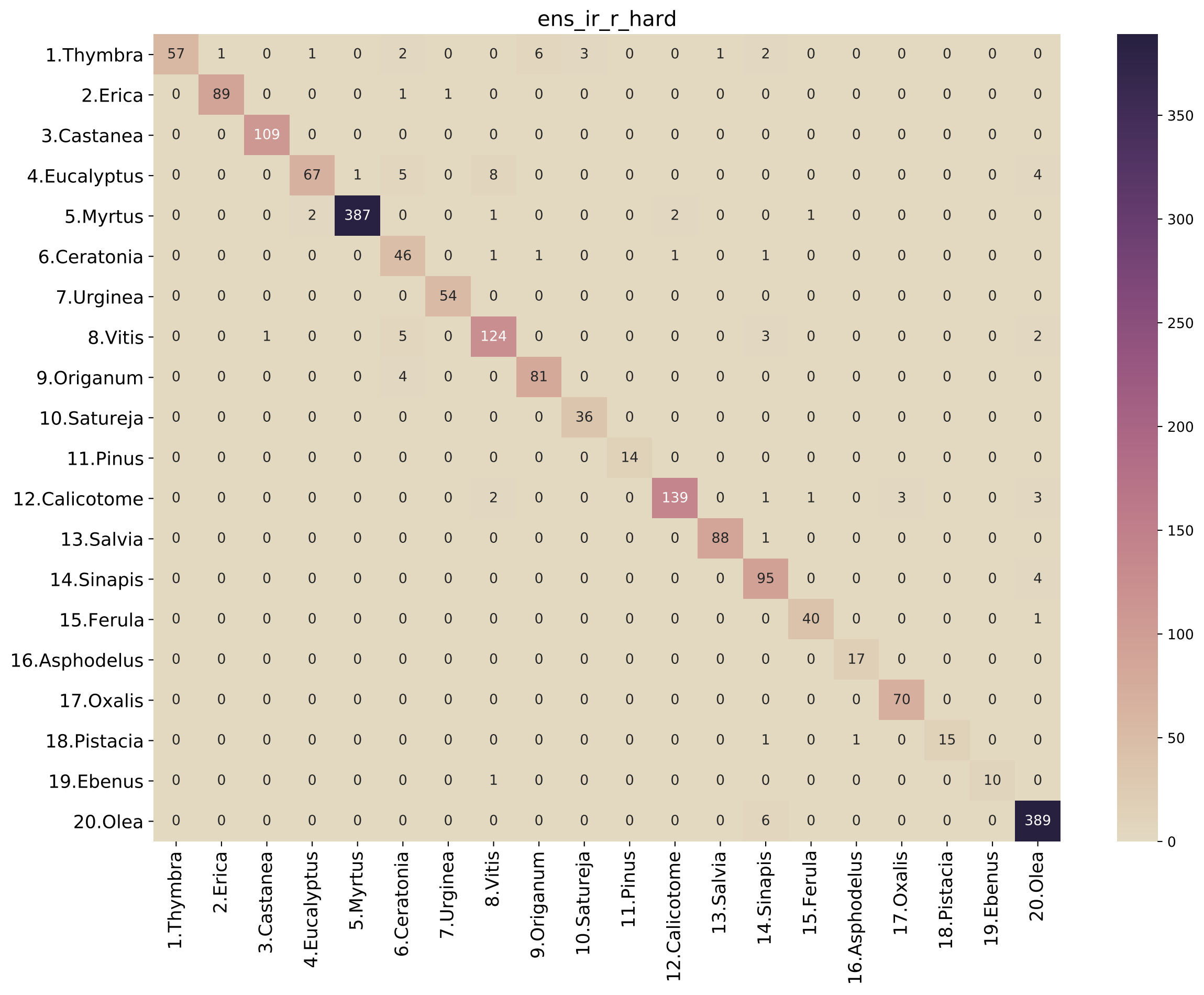

Supplement: Supplementary file 1 [file plants-11-00919-s001.zip › Supplementary-Images/confusion-matrices-of-all-models/ens_ir_r_hard_cm.pdf]

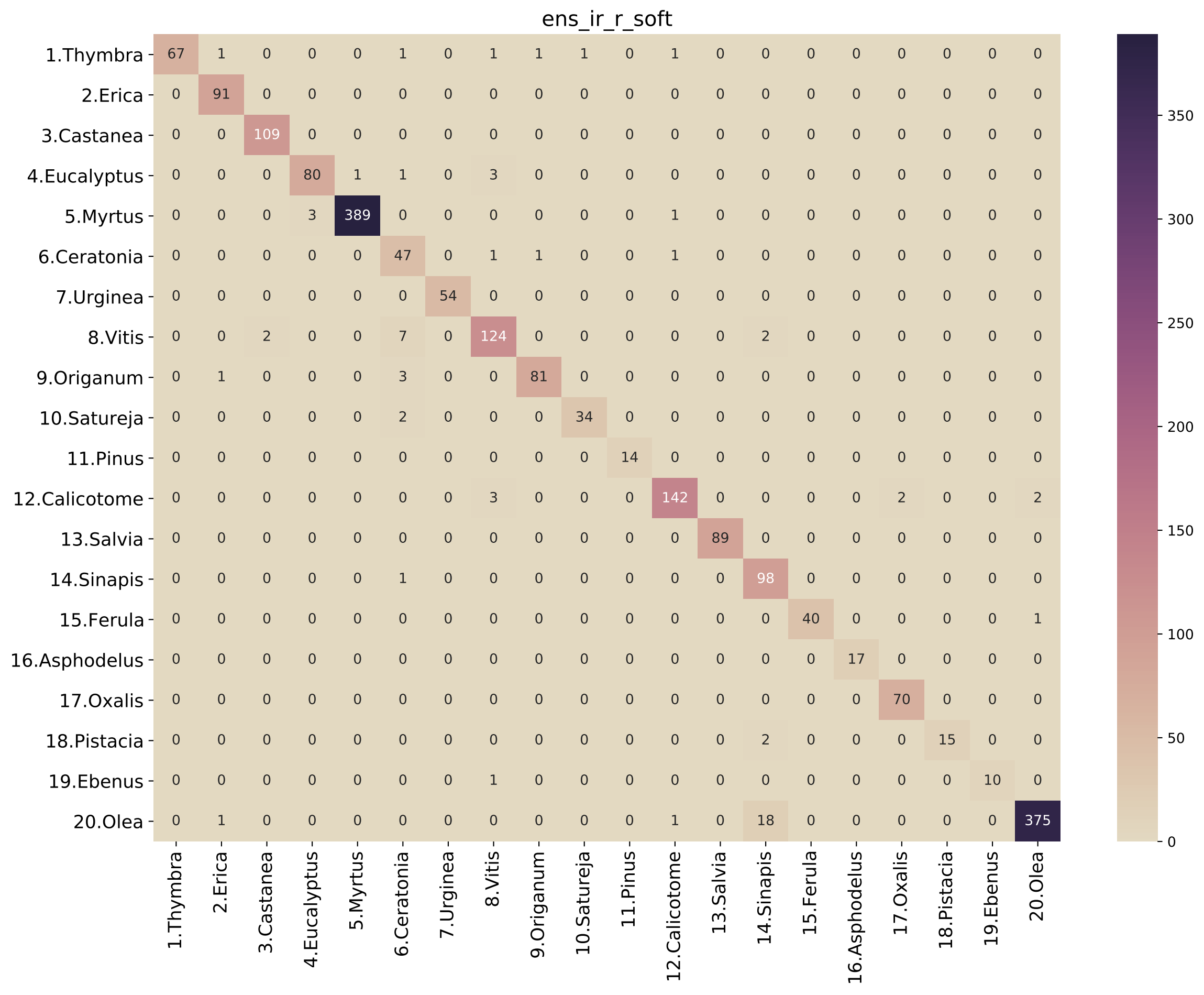

Supplement: Supplementary file 1 [file plants-11-00919-s001.zip › Supplementary-Images/confusion-matrices-of-all-models/ens_ir_r_soft_cm.pdf]

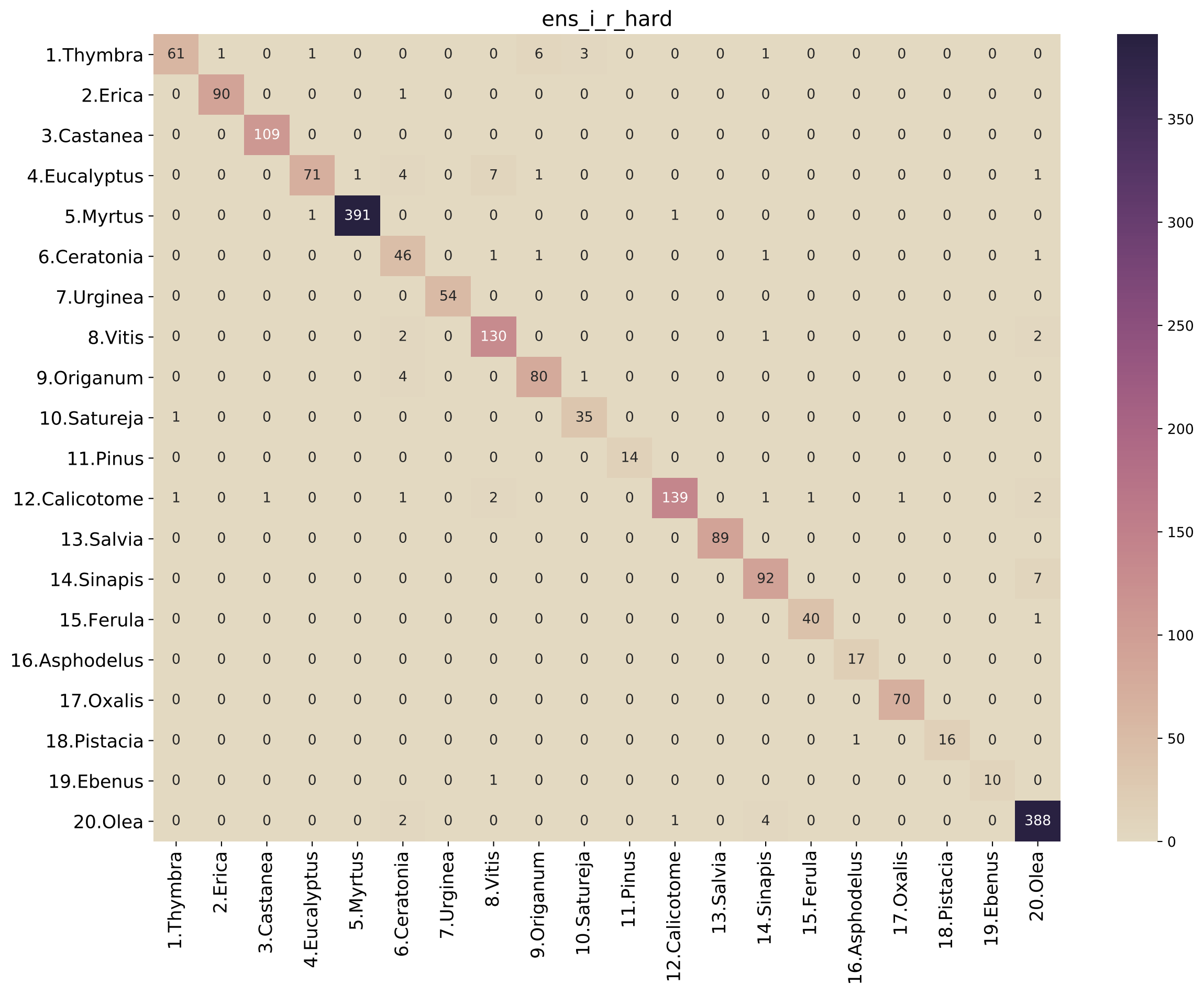

Supplement: Supplementary file 1 [file plants-11-00919-s001.zip › Supplementary-Images/confusion-matrices-of-all-models/ens_i_r_hard_cm.pdf]

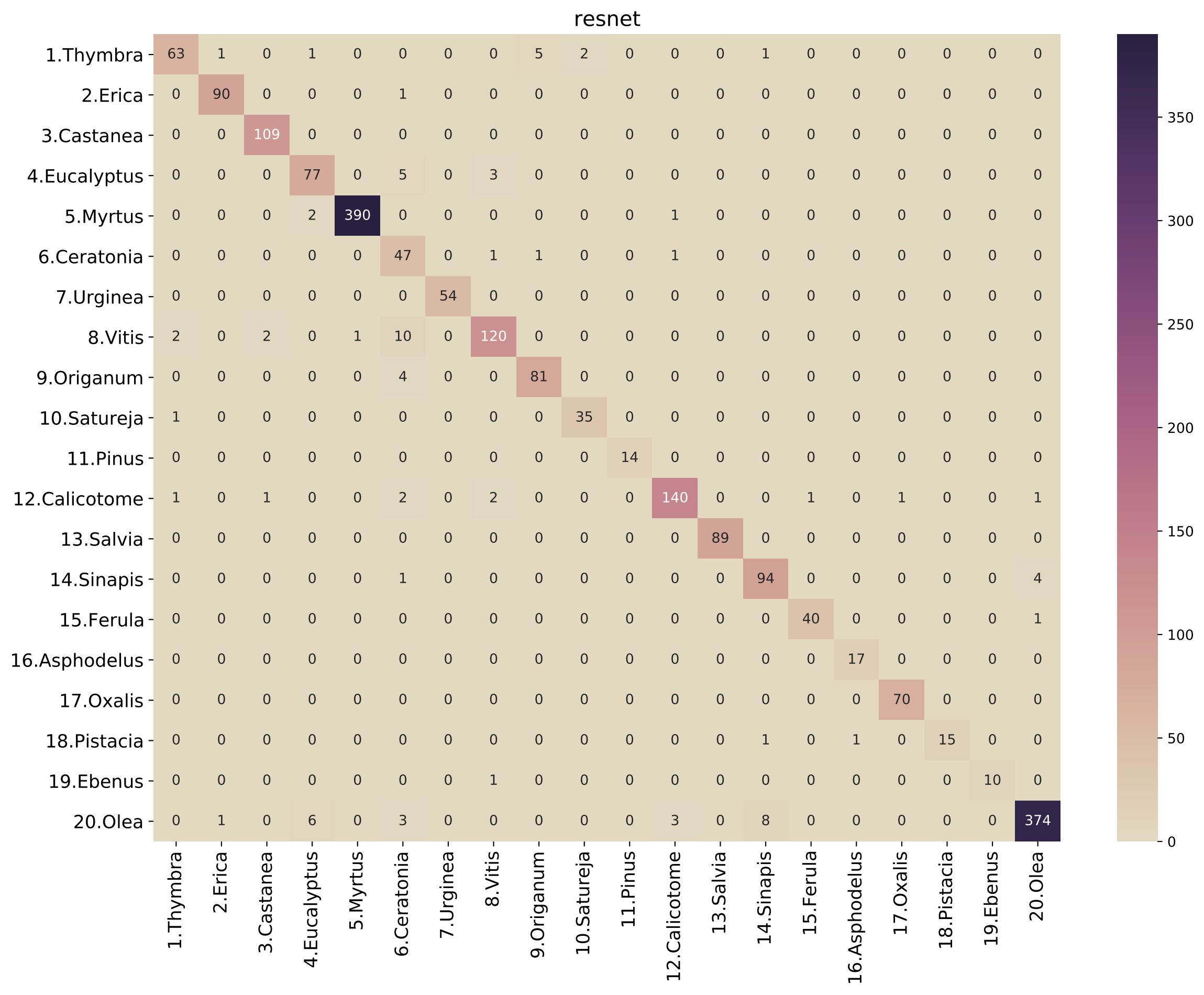

Supplement: Supplementary file 1 [file plants-11-00919-s001.zip › Supplementary-Images/confusion-matrices-of-all-models/resnet_cm.pdf]

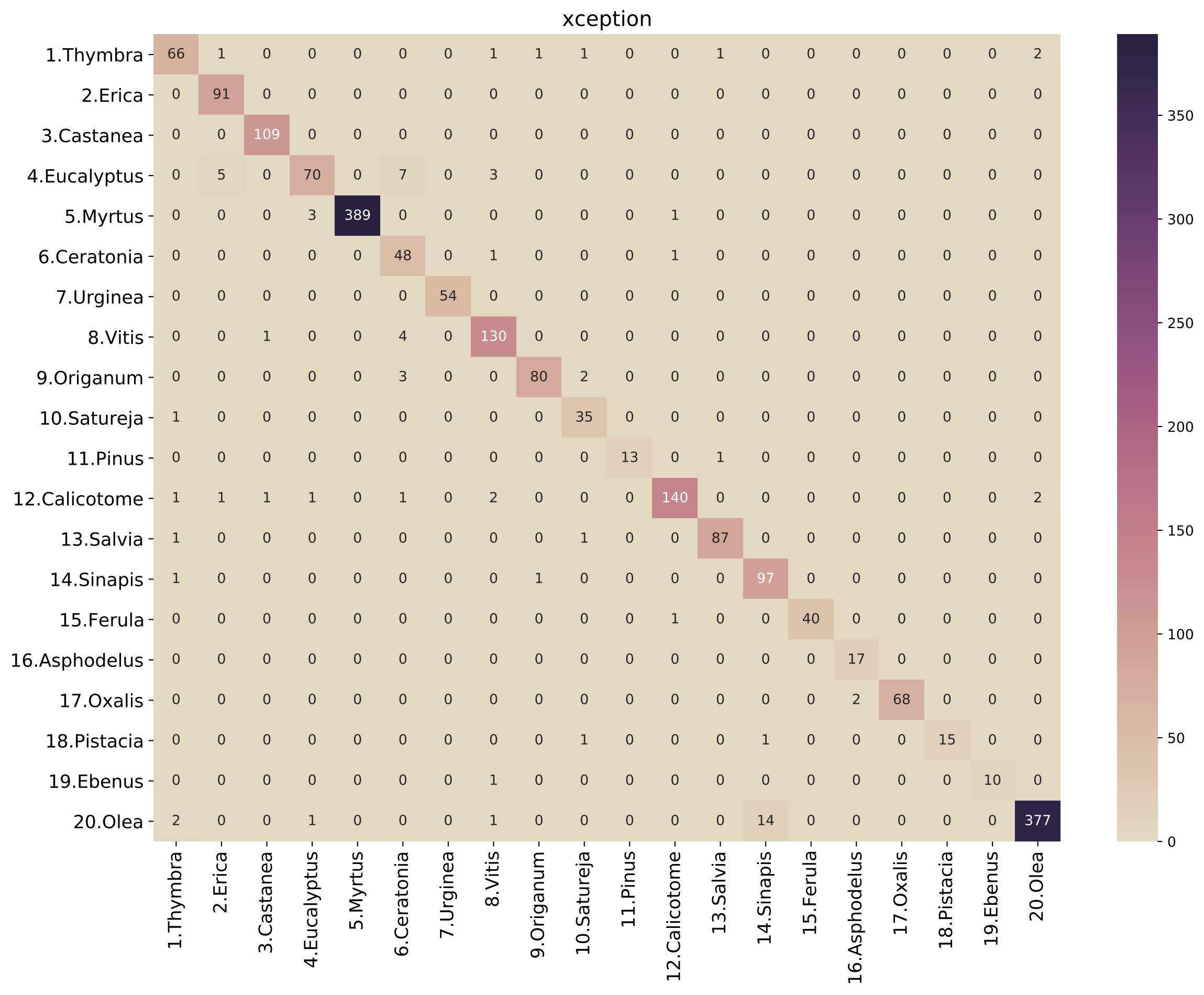

Supplement: Supplementary file 1 [file plants-11-00919-s001.zip › Supplementary-Images/confusion-matrices-of-all-models/xception_cm.pdf]

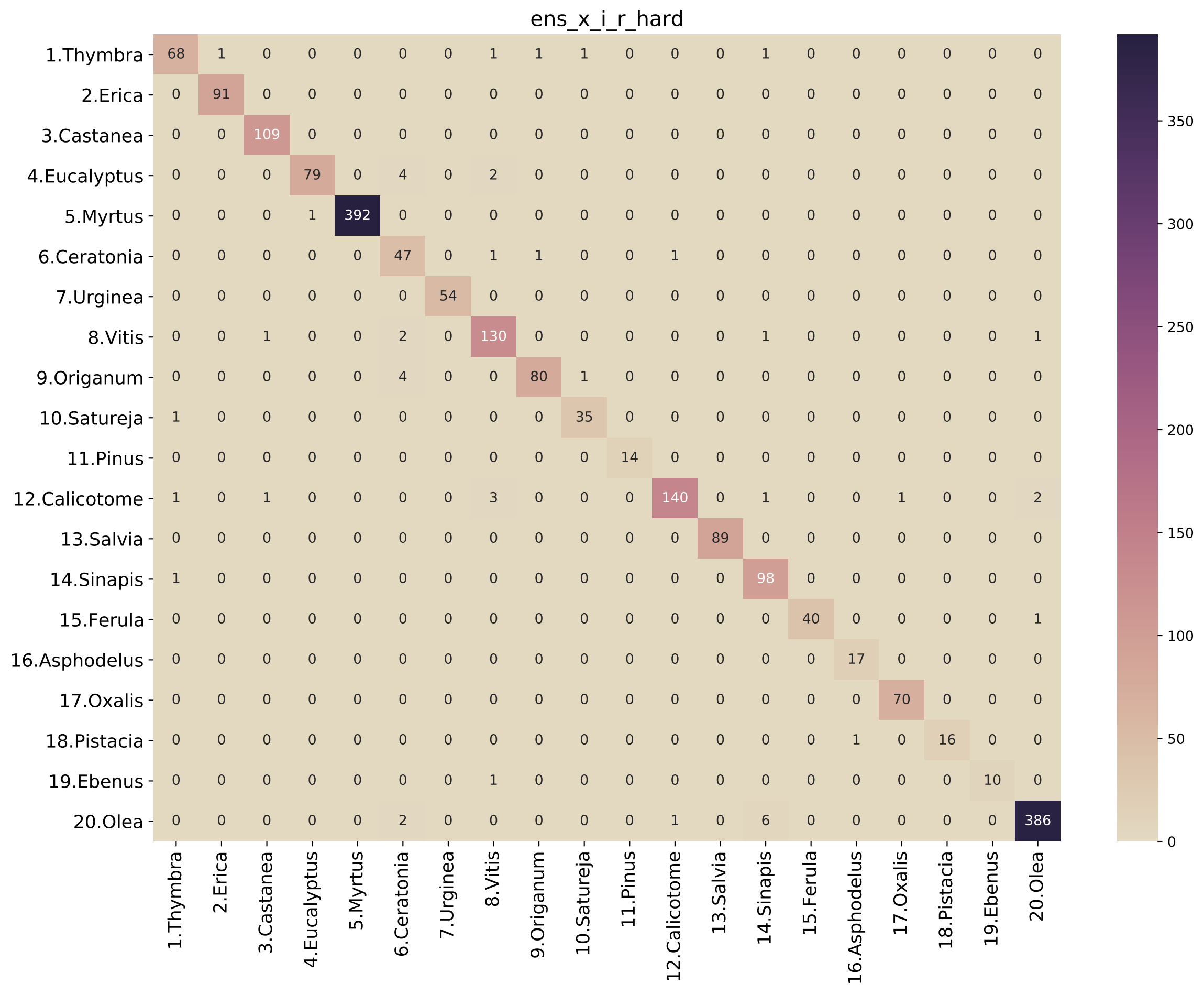

Supplement: Supplementary file 1 [file plants-11-00919-s001.zip › Supplementary-Images/confusion-matrices-of-all-models/ens_x_i_r_hard_cm.pdf]

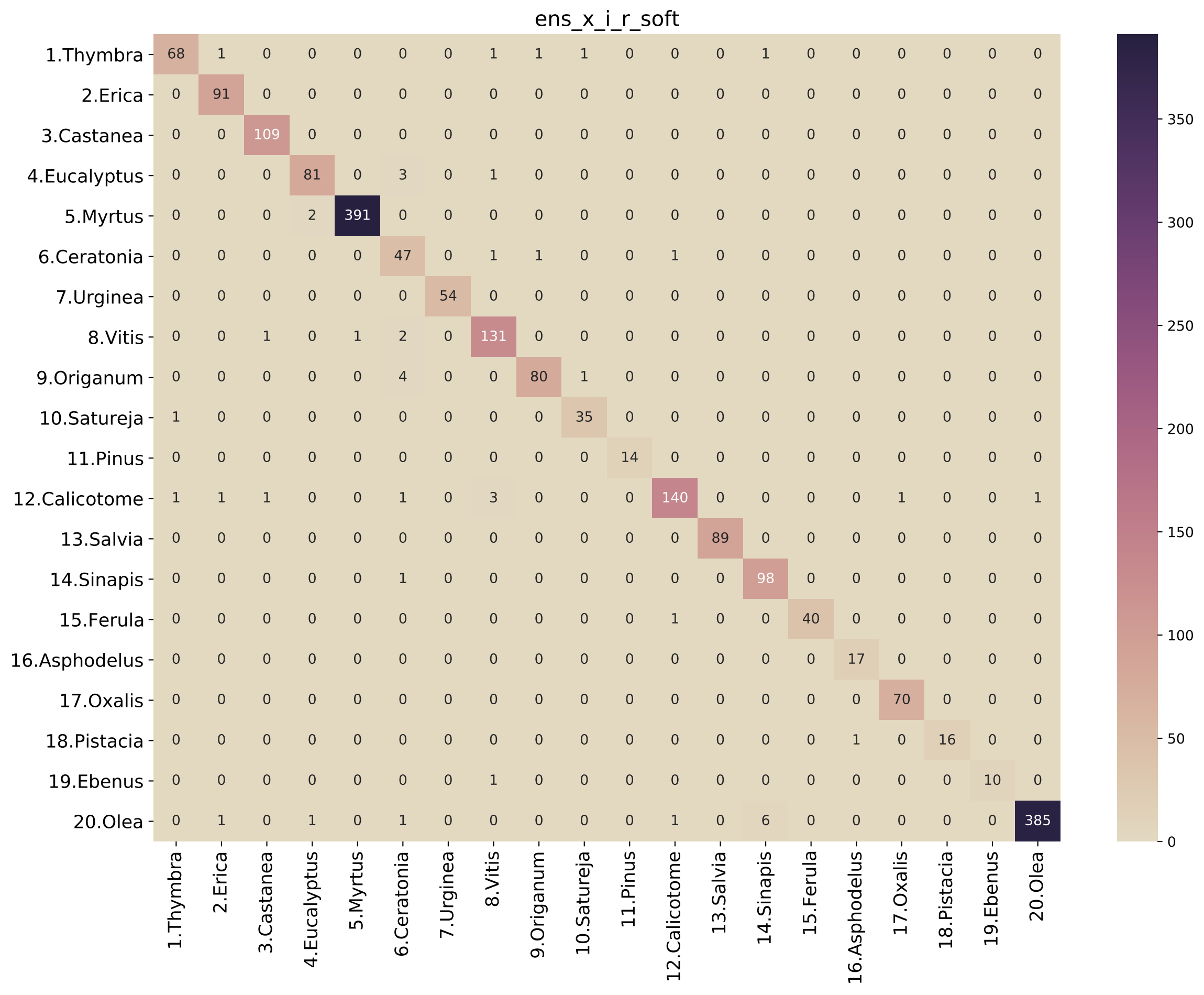

Supplement: Supplementary file 1 [file plants-11-00919-s001.zip › Supplementary-Images/confusion-matrices-of-all-models/ens_x_i_r_soft_cm.pdf]

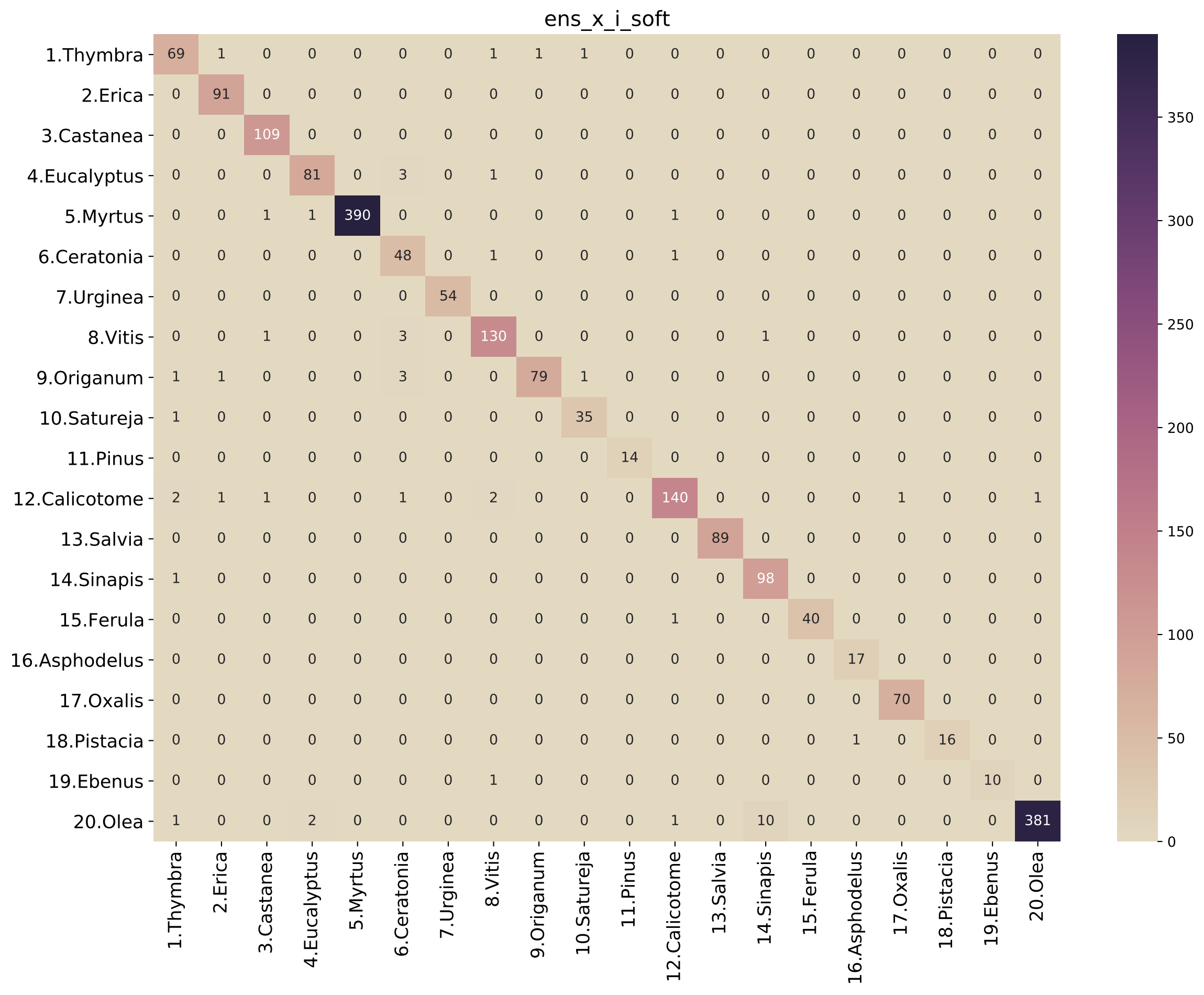

Supplement: Supplementary file 1 [file plants-11-00919-s001.zip › Supplementary-Images/confusion-matrices-of-all-models/ens_x_i_soft_cm.pdf]

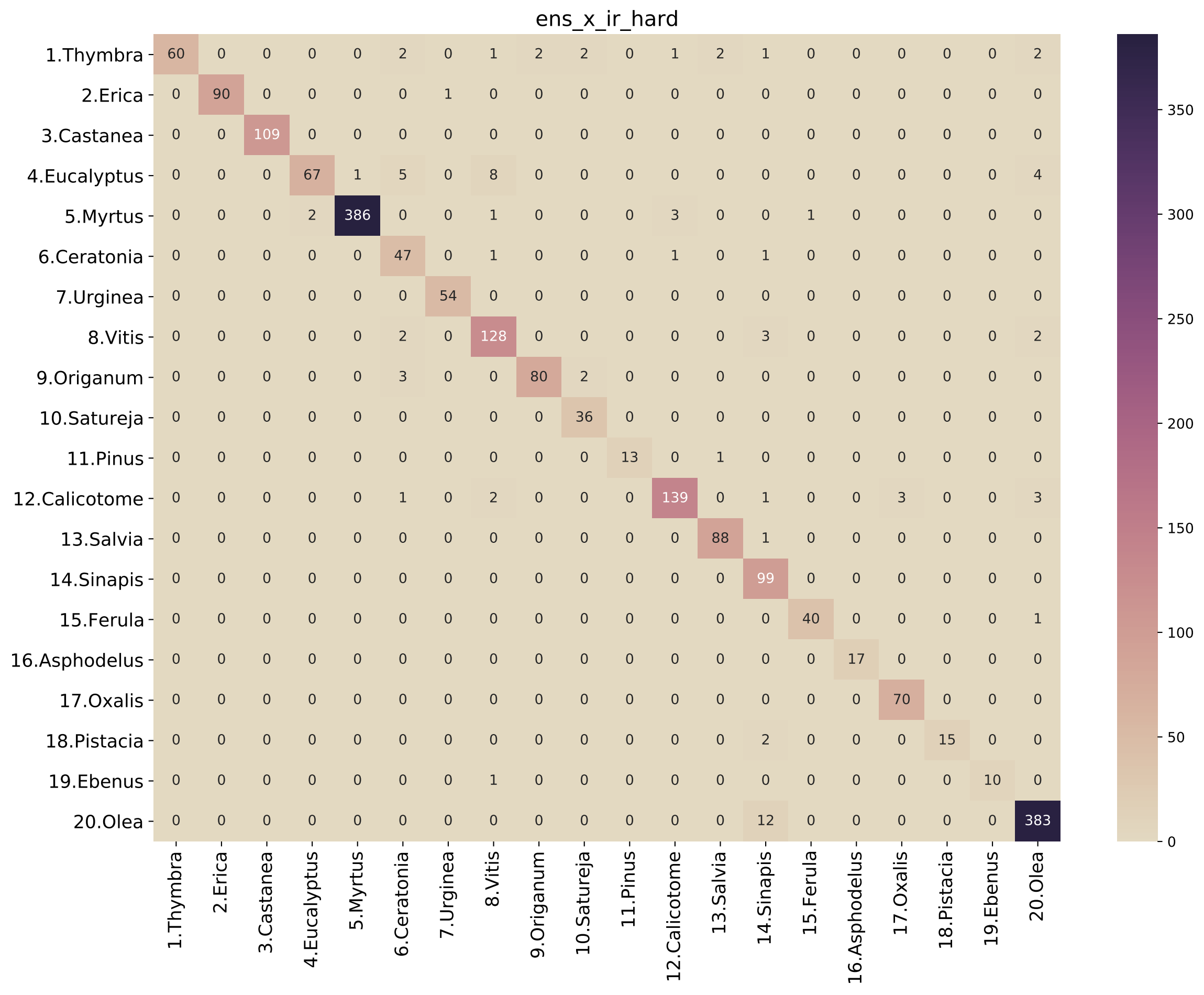

Supplement: Supplementary file 1 [file plants-11-00919-s001.zip › Supplementary-Images/confusion-matrices-of-all-models/ens_x_ir_hard_cm.pdf]

ens\_x\_ir\_i\_hard

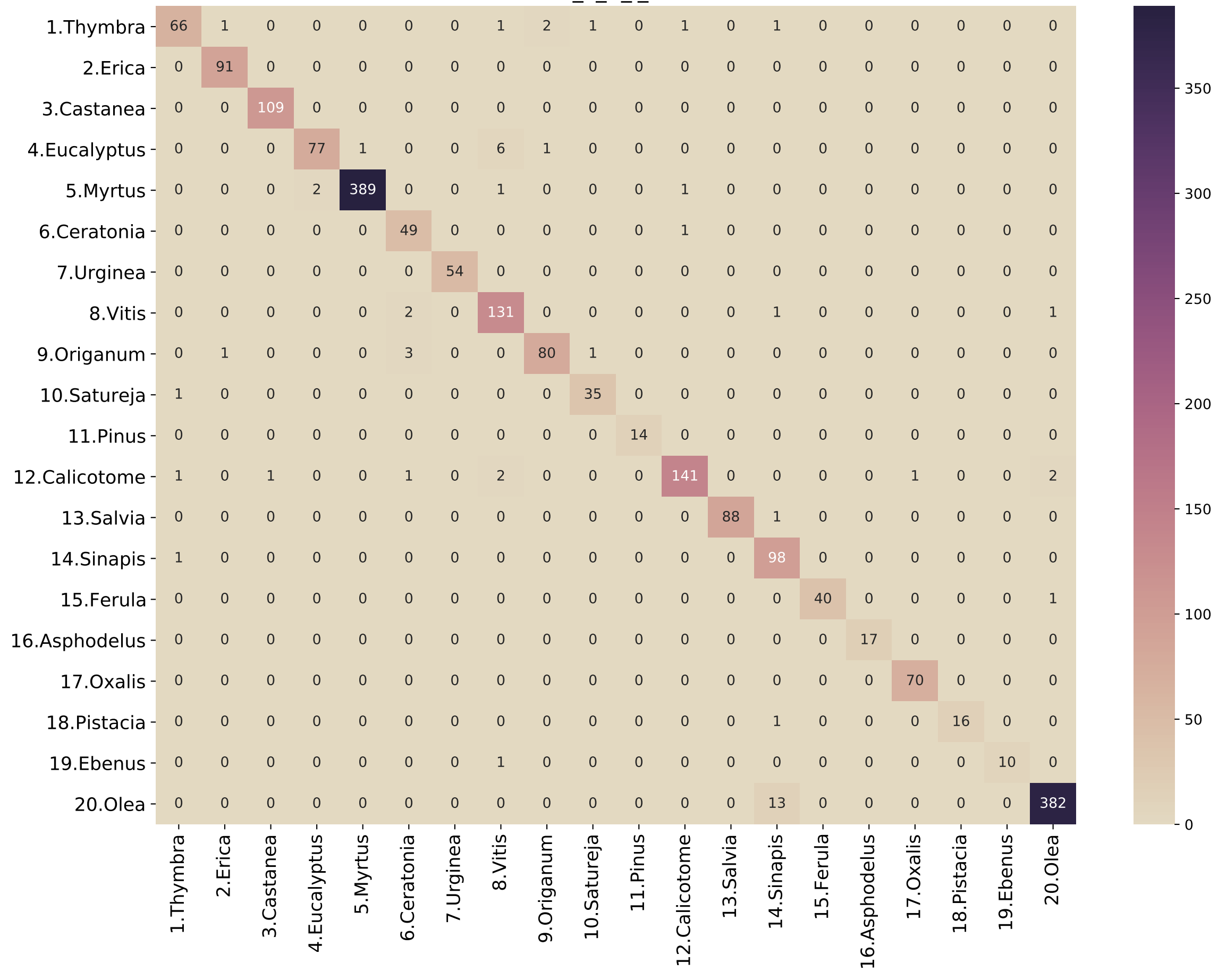

Supplement: Supplementary file 1 [file plants-11-00919-s001.zip › Supplementary-Images/confusion-matrices-of-all-models/ens_x_ir_i_hard_cm.pdf]

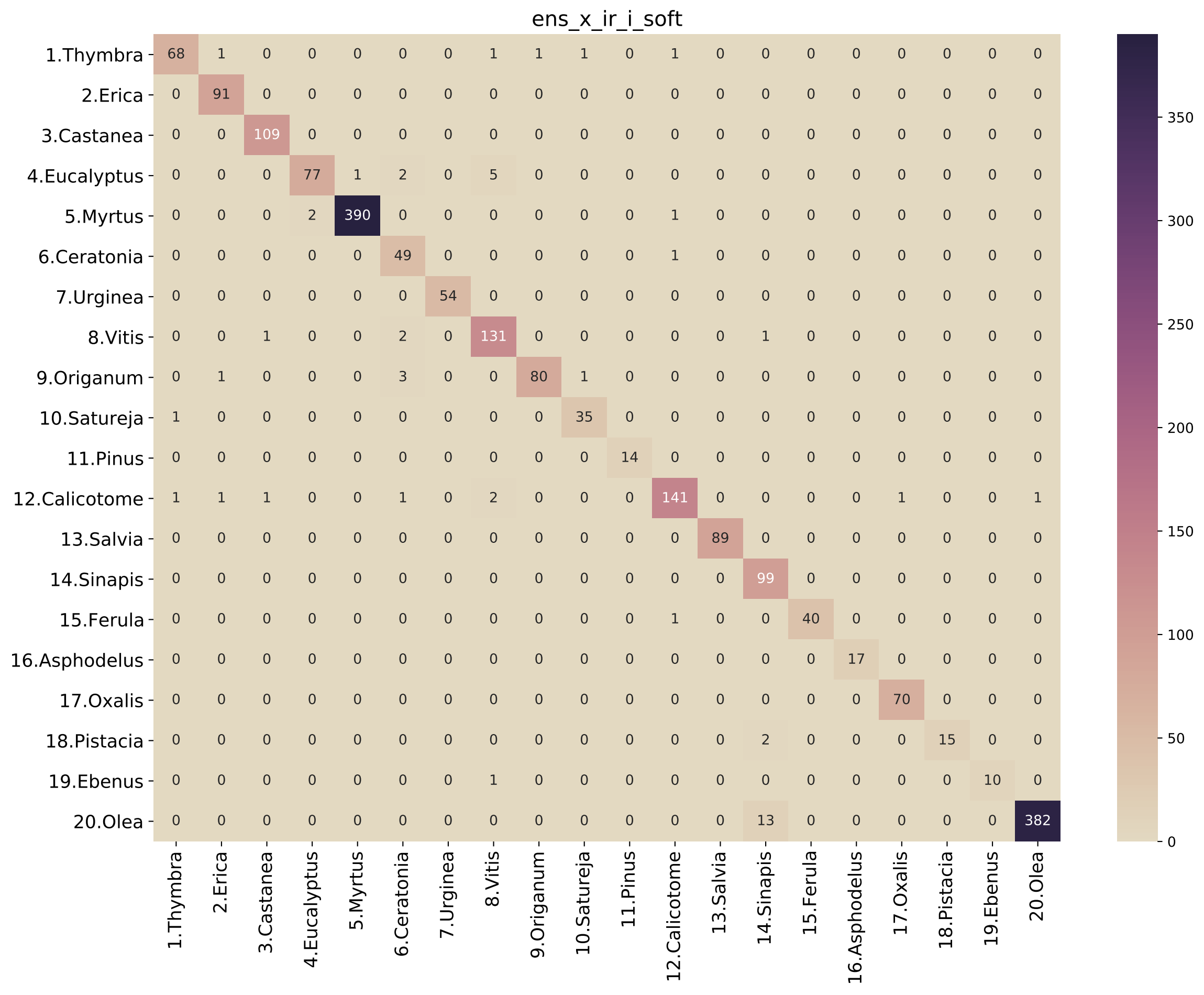

Supplement: Supplementary file 1 [file plants-11-00919-s001.zip › Supplementary-Images/confusion-matrices-of-all-models/ens_x_ir_i_soft_cm.pdf]

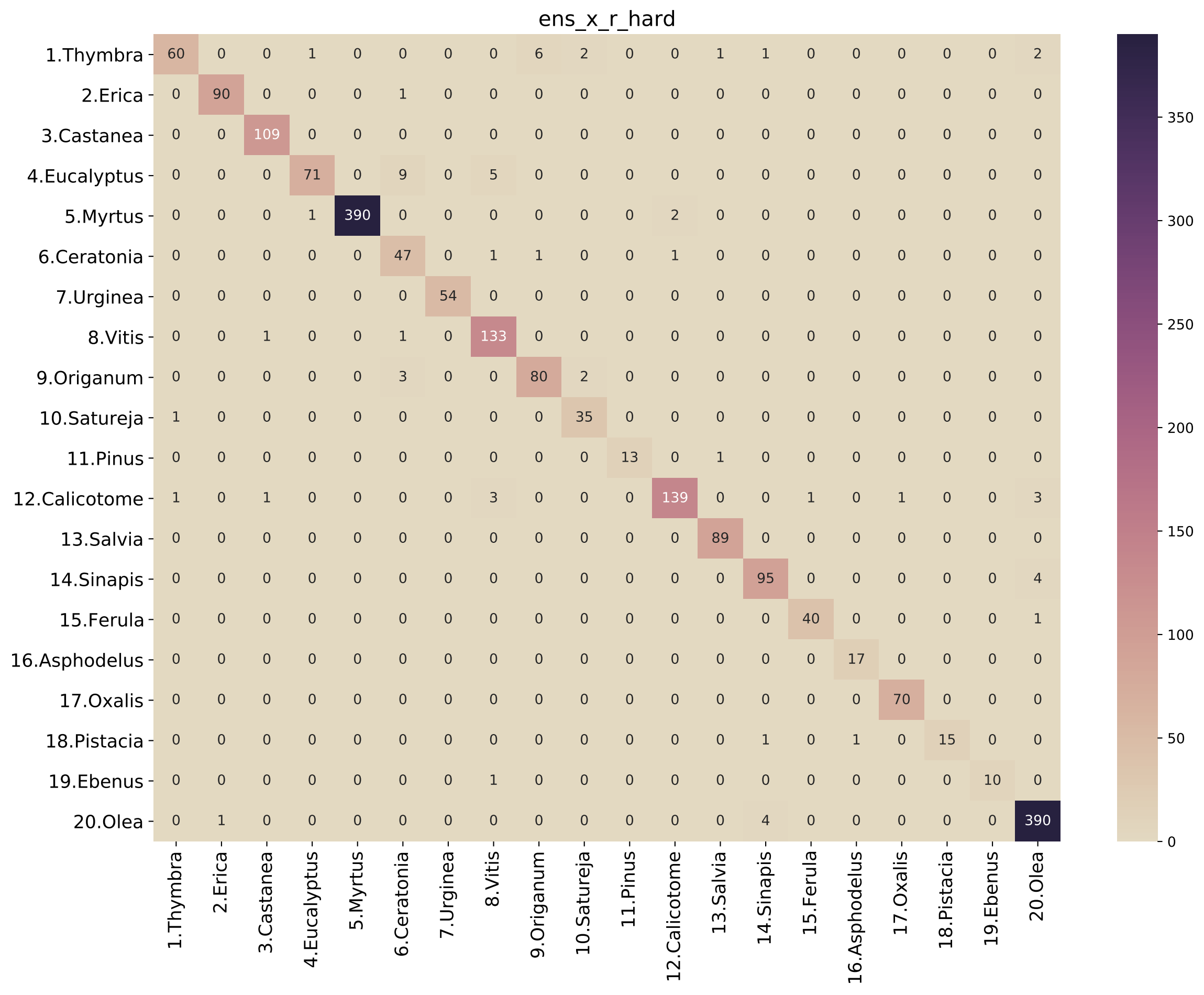

Supplement: Supplementary file 1 [file plants-11-00919-s001.zip › Supplementary-Images/confusion-matrices-of-all-models/ens_x_r_hard_cm.pdf]

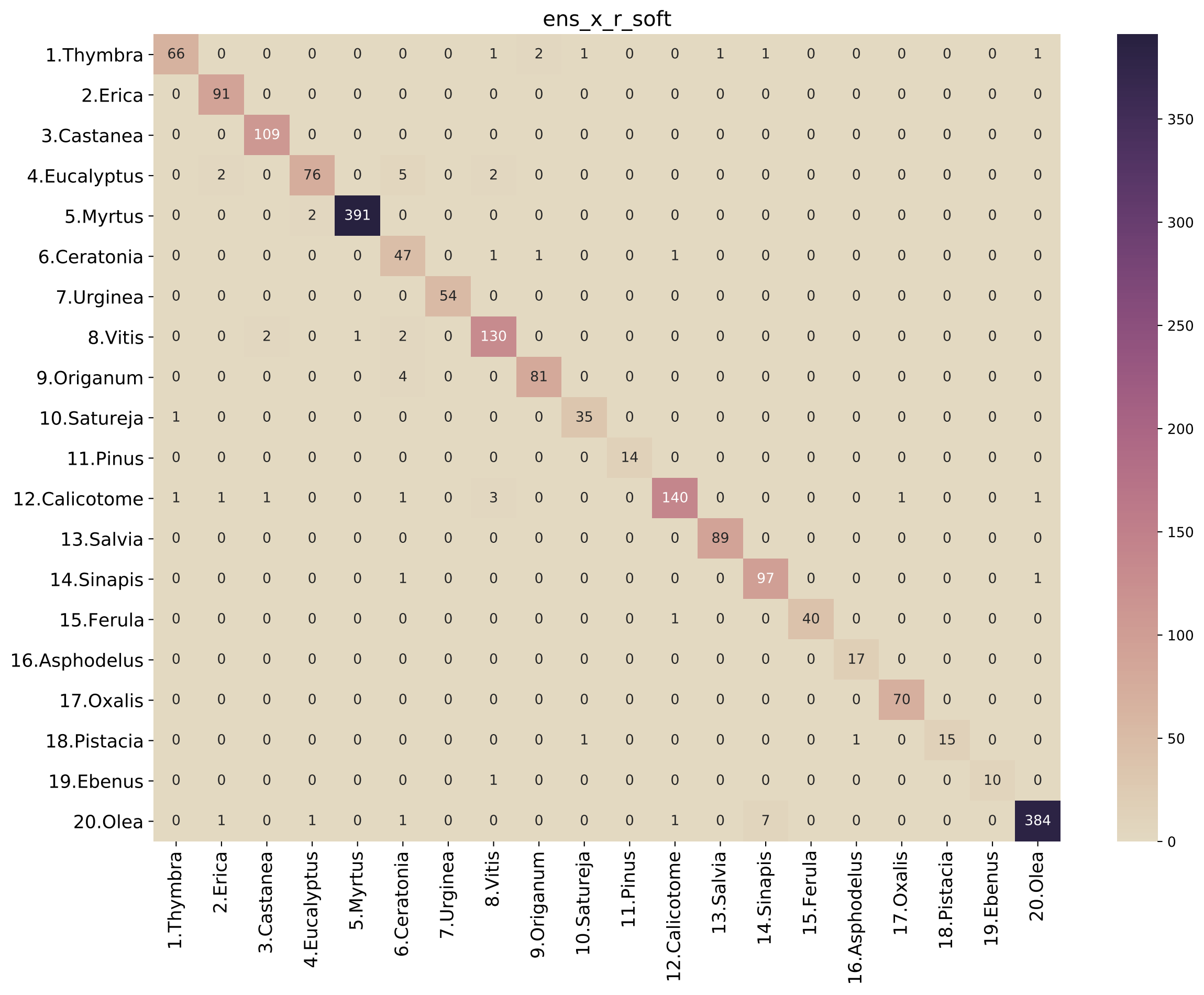

Supplement: Supplementary file 1 [file plants-11-00919-s001.zip › Supplementary-Images/confusion-matrices-of-all-models/ens_x_r_soft_cm.pdf]

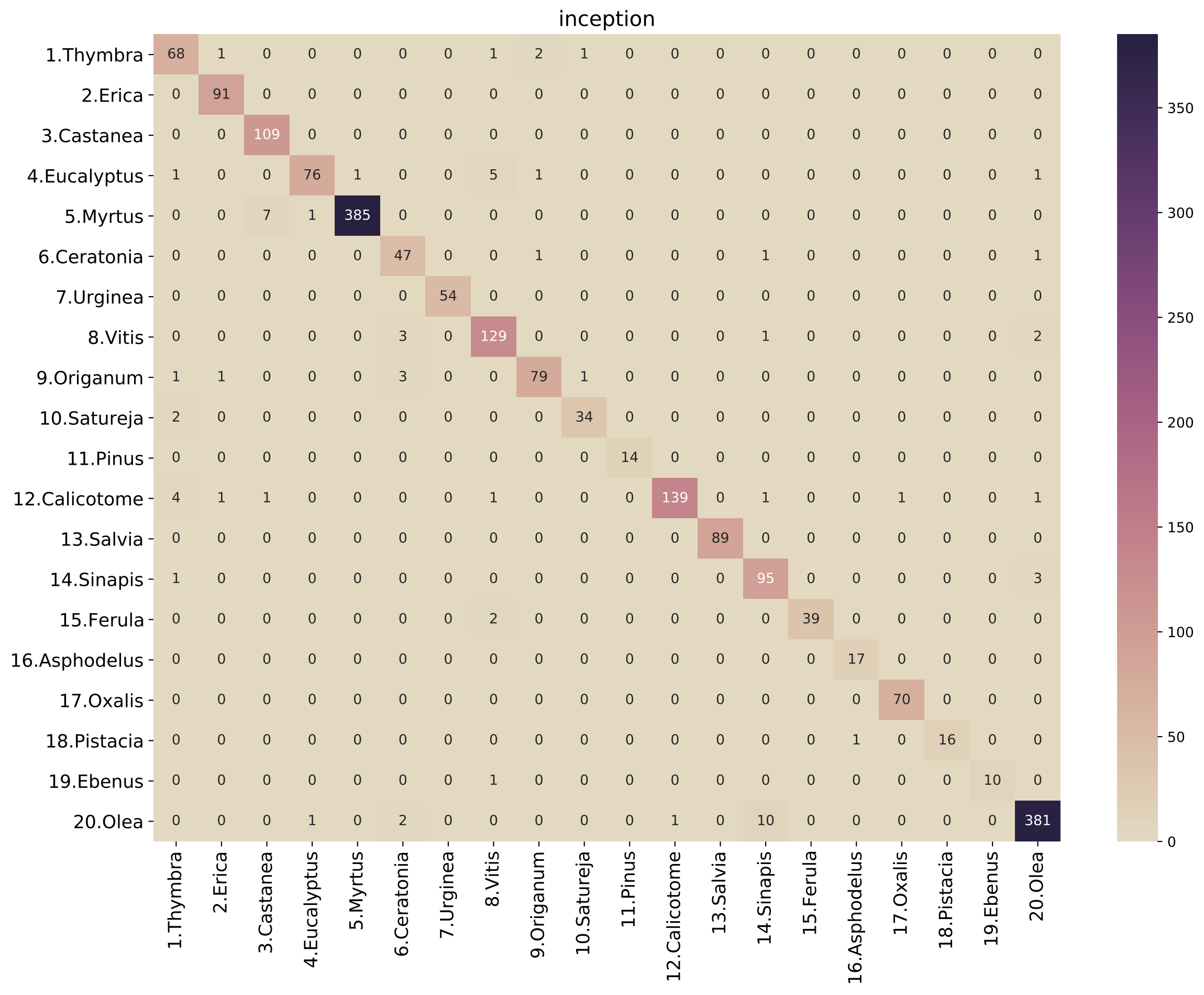

Supplement: Supplementary file 1 [file plants-11-00919-s001.zip › Supplementary-Images/confusion-matrices-of-all-models/inception_cm.pdf]

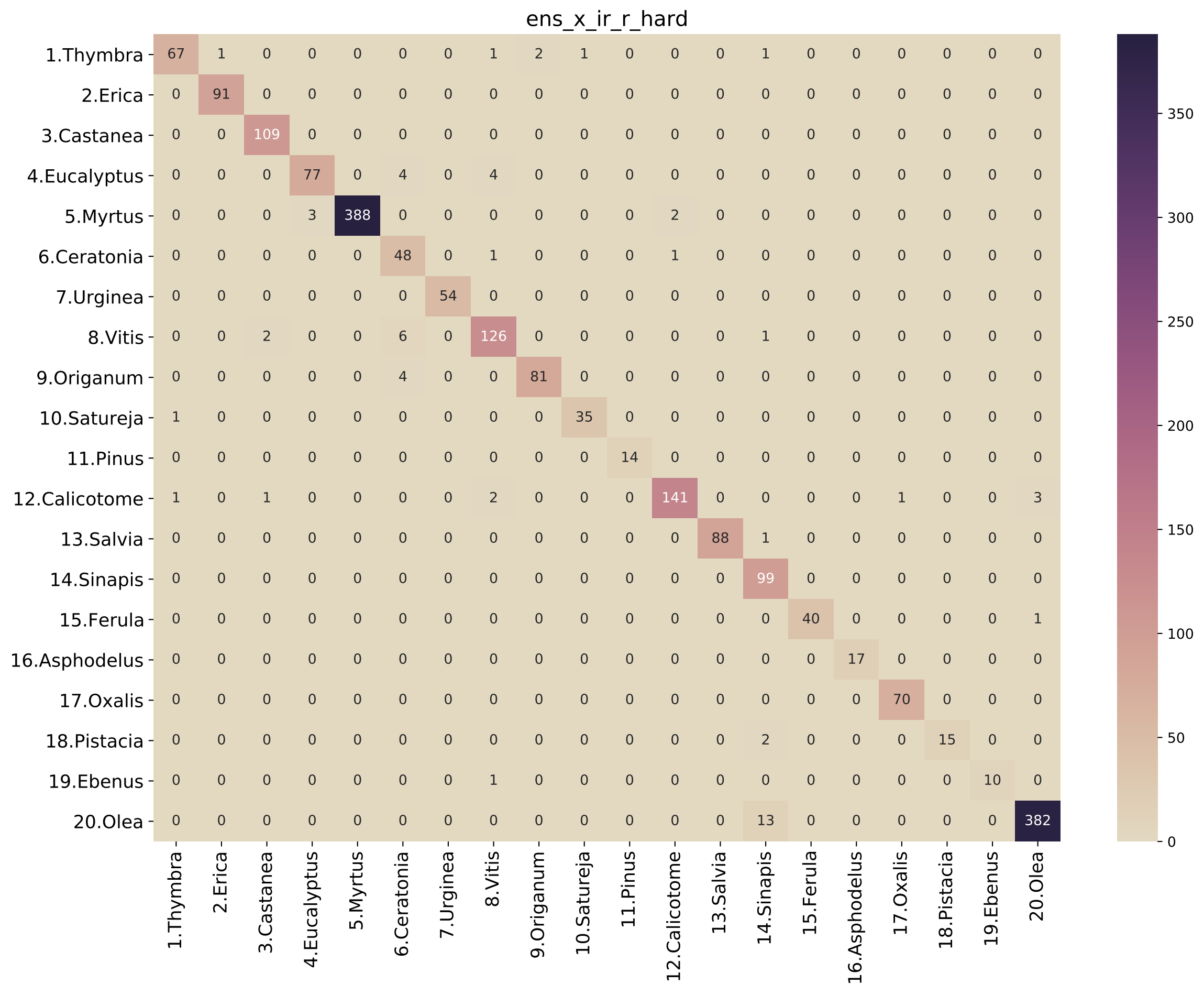

Supplement: Supplementary file 1 [file plants-11-00919-s001.zip › Supplementary-Images/confusion-matrices-of-all-models/ens_x_ir_r_hard_cm.pdf]

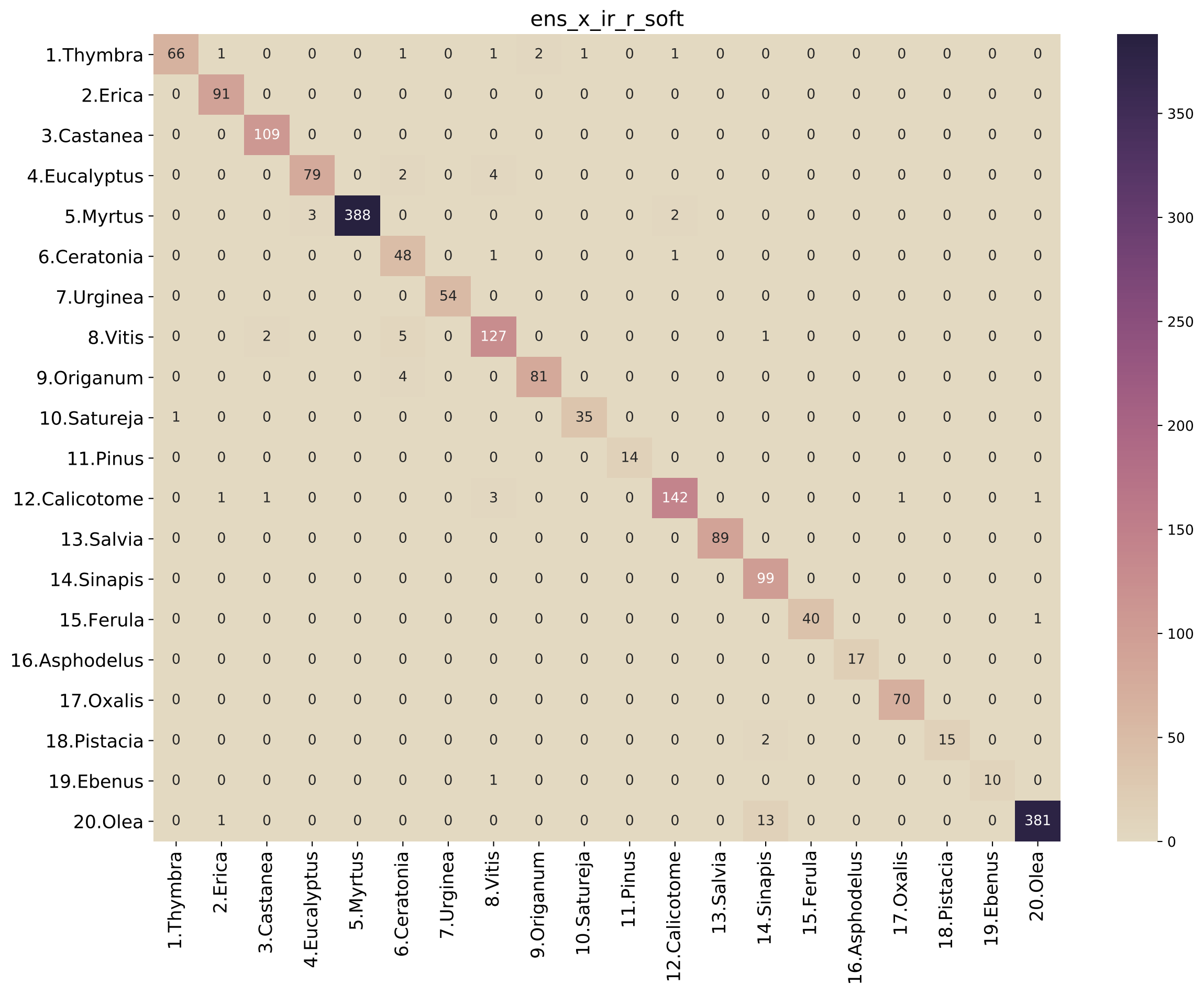

Supplement: Supplementary file 1 [file plants-11-00919-s001.zip › Supplementary-Images/confusion-matrices-of-all-models/ens_x_ir_r_soft_cm.pdf]

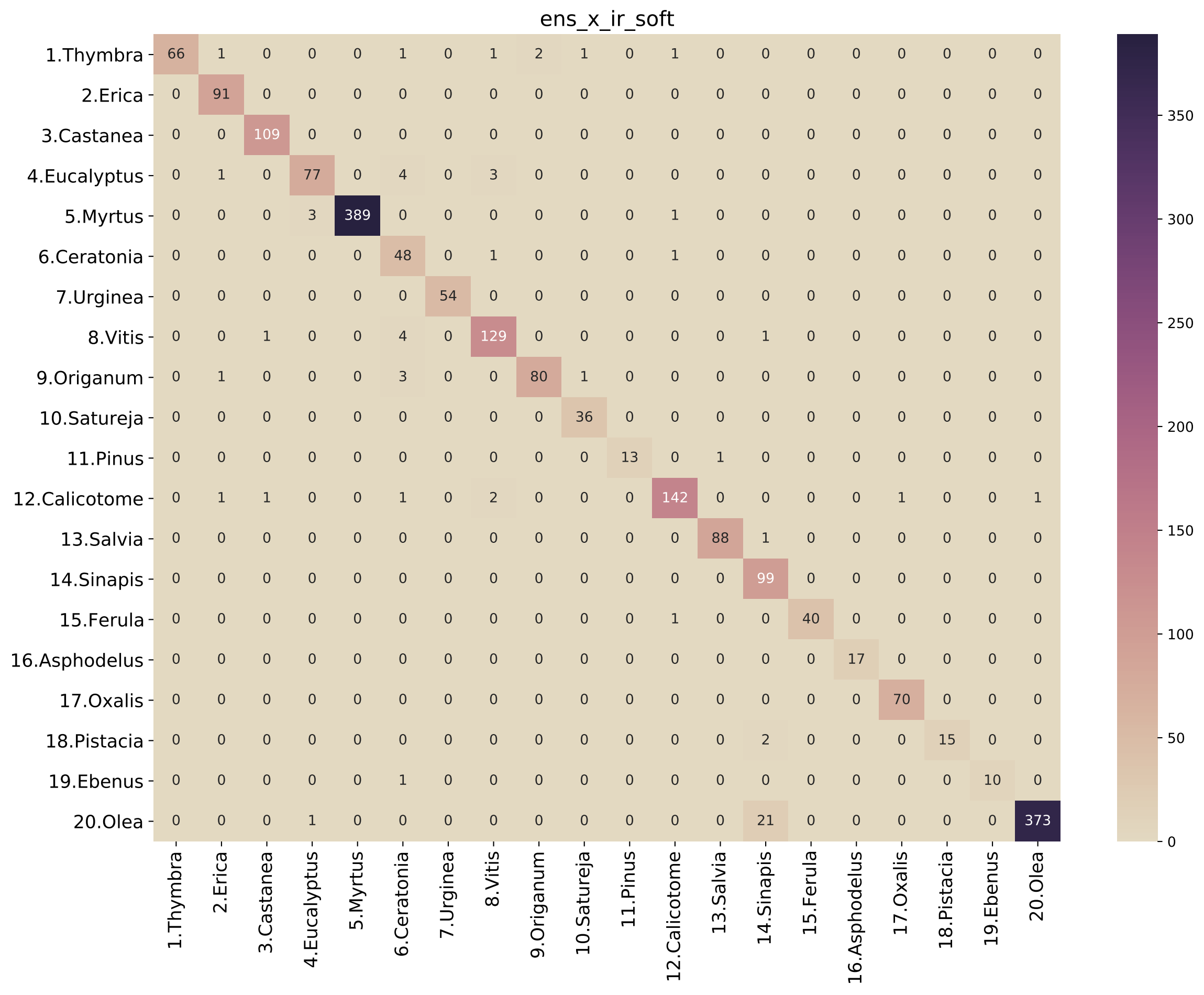

Supplement: Supplementary file 1 [file plants-11-00919-s001.zip › Supplementary-Images/confusion-matrices-of-all-models/ens_x_ir_soft_cm.pdf]

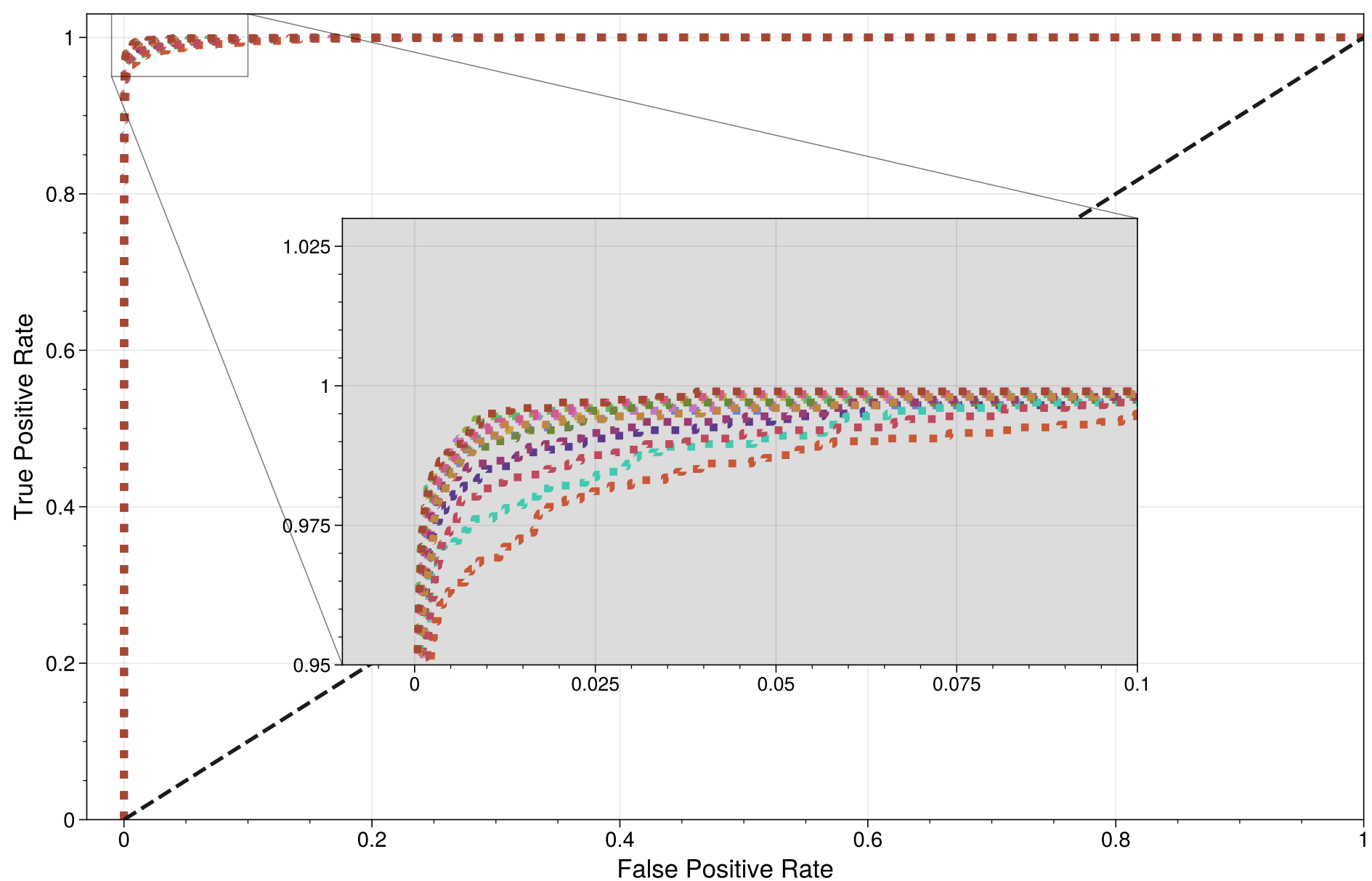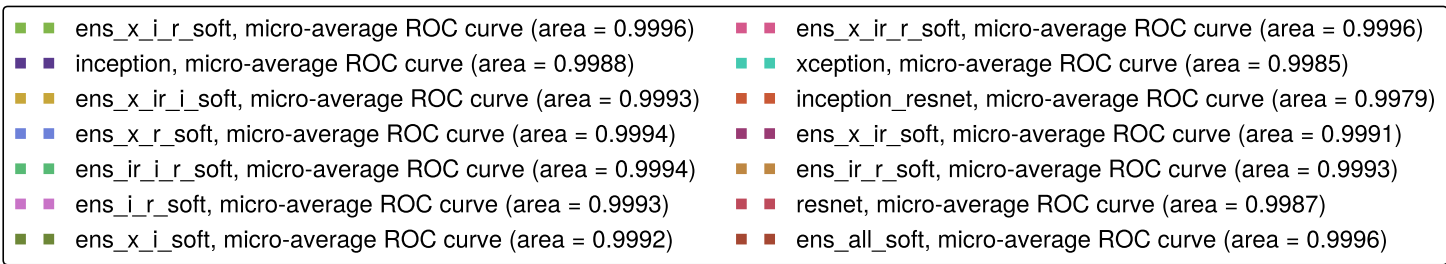

Supplement: Supplementary file 1 [file plants-11-00919-s001.zip › Supplementary-Images/roc-curves-of-all-models/micro_average_roc.pdf]

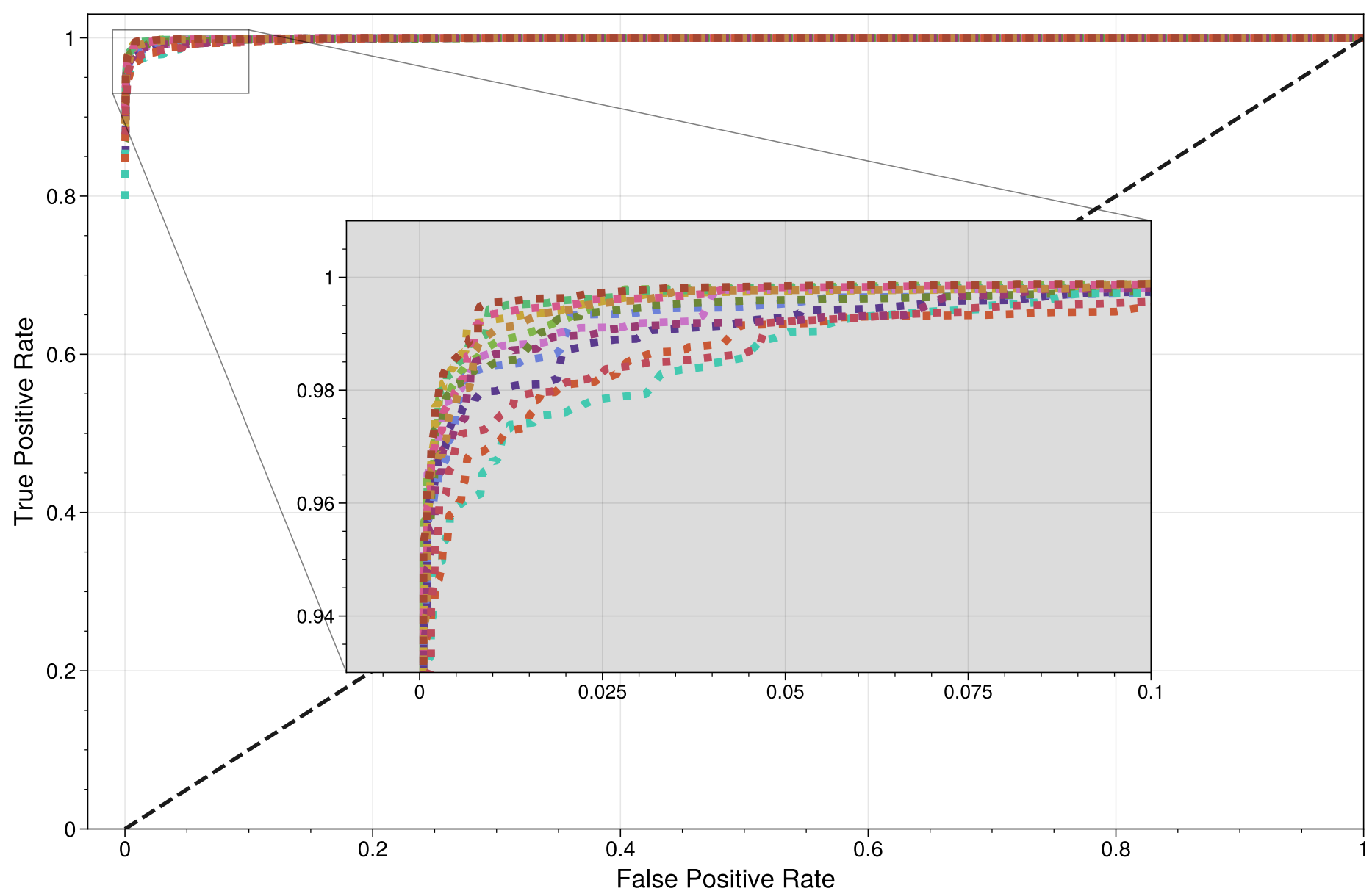

Supplement: Supplementary file 1 [file plants-11-00919-s001.zip › Supplementary-Images/roc-curves-of-all-models/macro_average_roc.pdf]

ens\_all\_soft

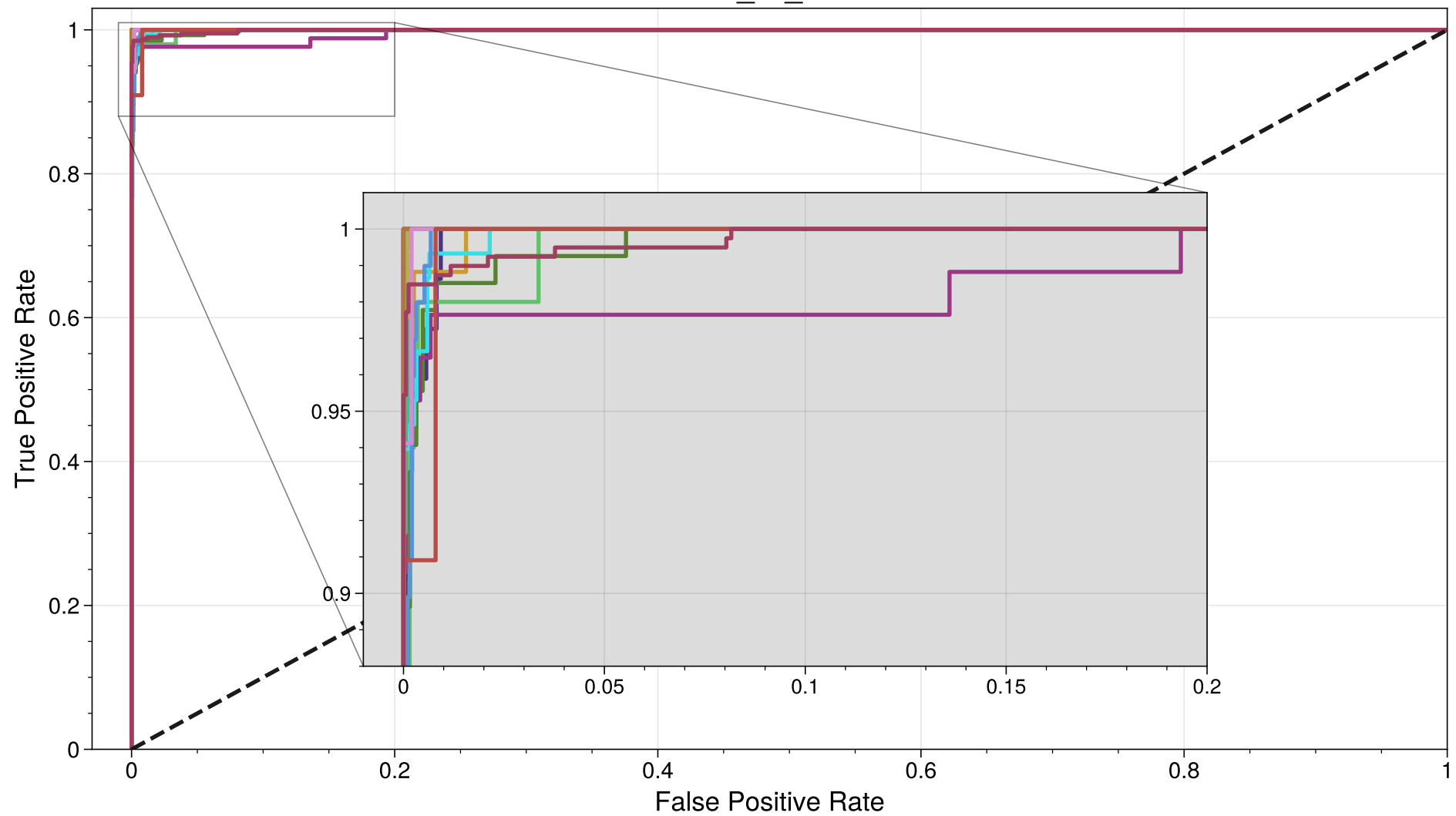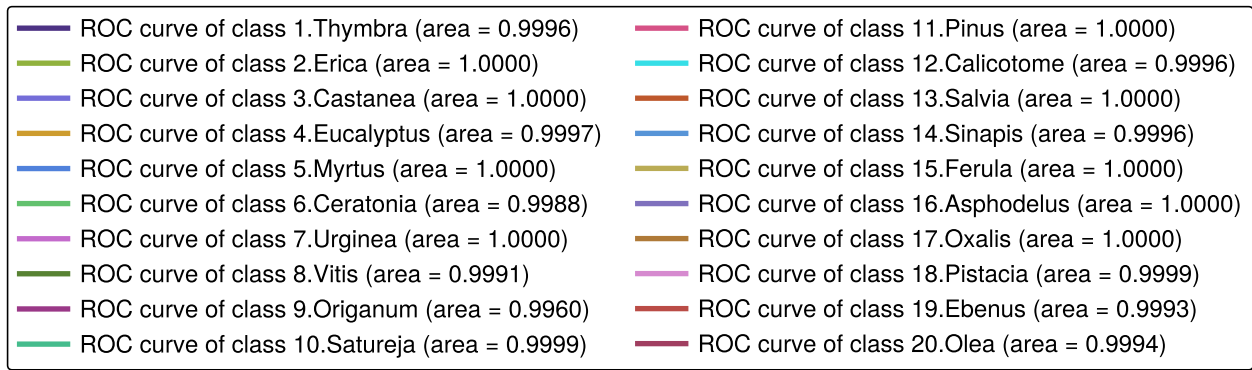

Supplement: Supplementary file 1 [file plants-11-00919-s001.zip › Supplementary-Images/roc-curves-of-all-models/ens_all_soft_roc.pdf]

resnet

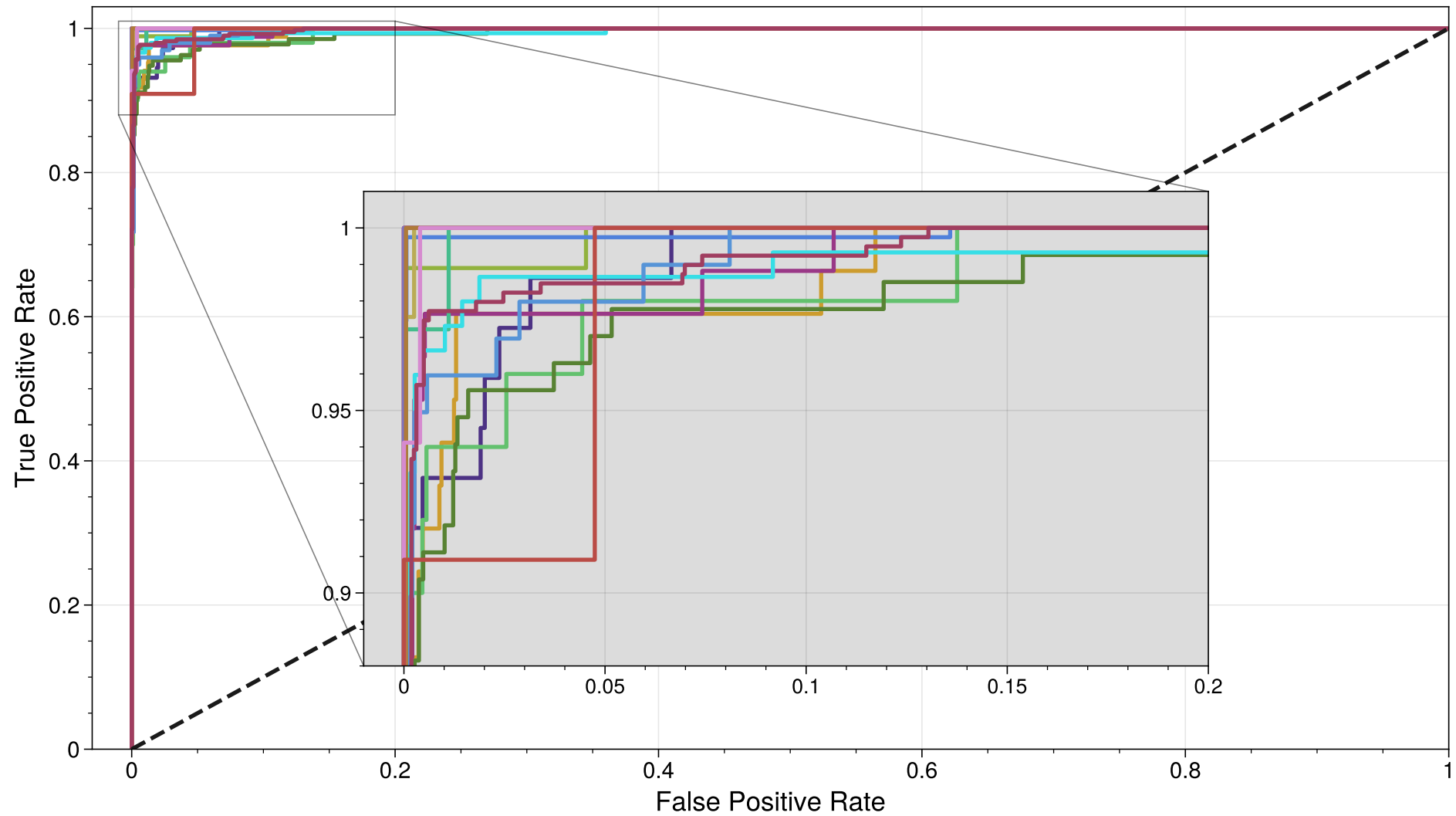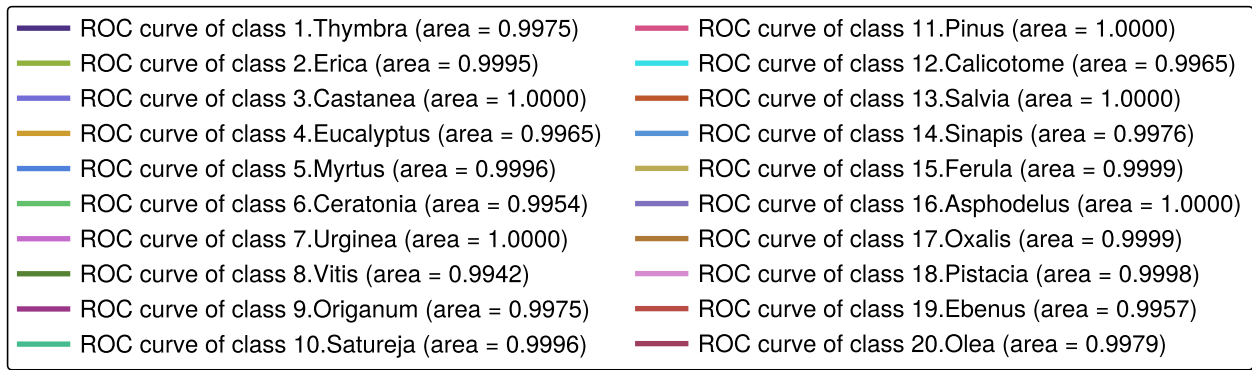

Supplement: Supplementary file 1 [file plants-11-00919-s001.zip › Supplementary-Images/roc-curves-of-all-models/resnet_roc.pdf]

ens\_x\_i\_r\_soft

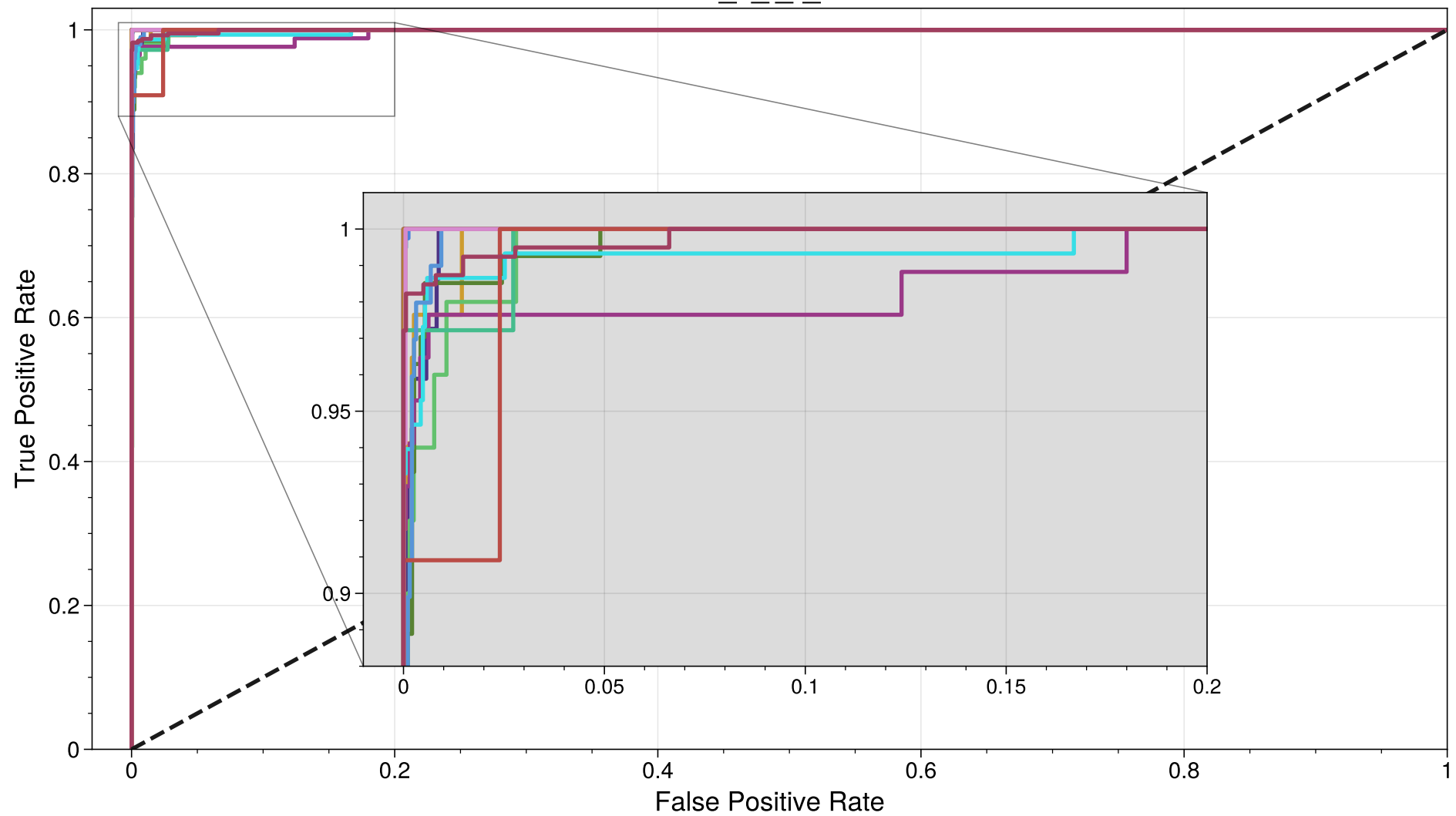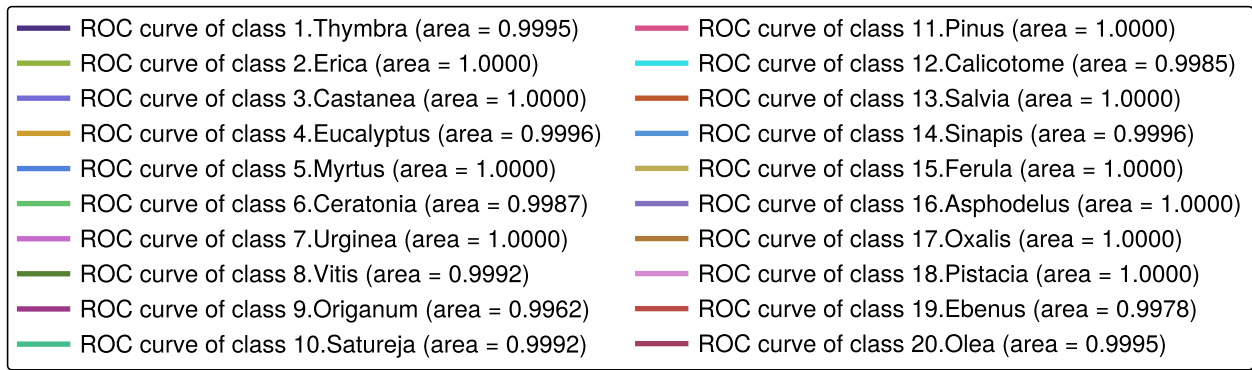

Supplement: Supplementary file 1 [file plants-11-00919-s001.zip › Supplementary-Images/roc-curves-of-all-models/ens_x_i_r_soft_roc.pdf]

ens\_ir\_i\_r\_soft

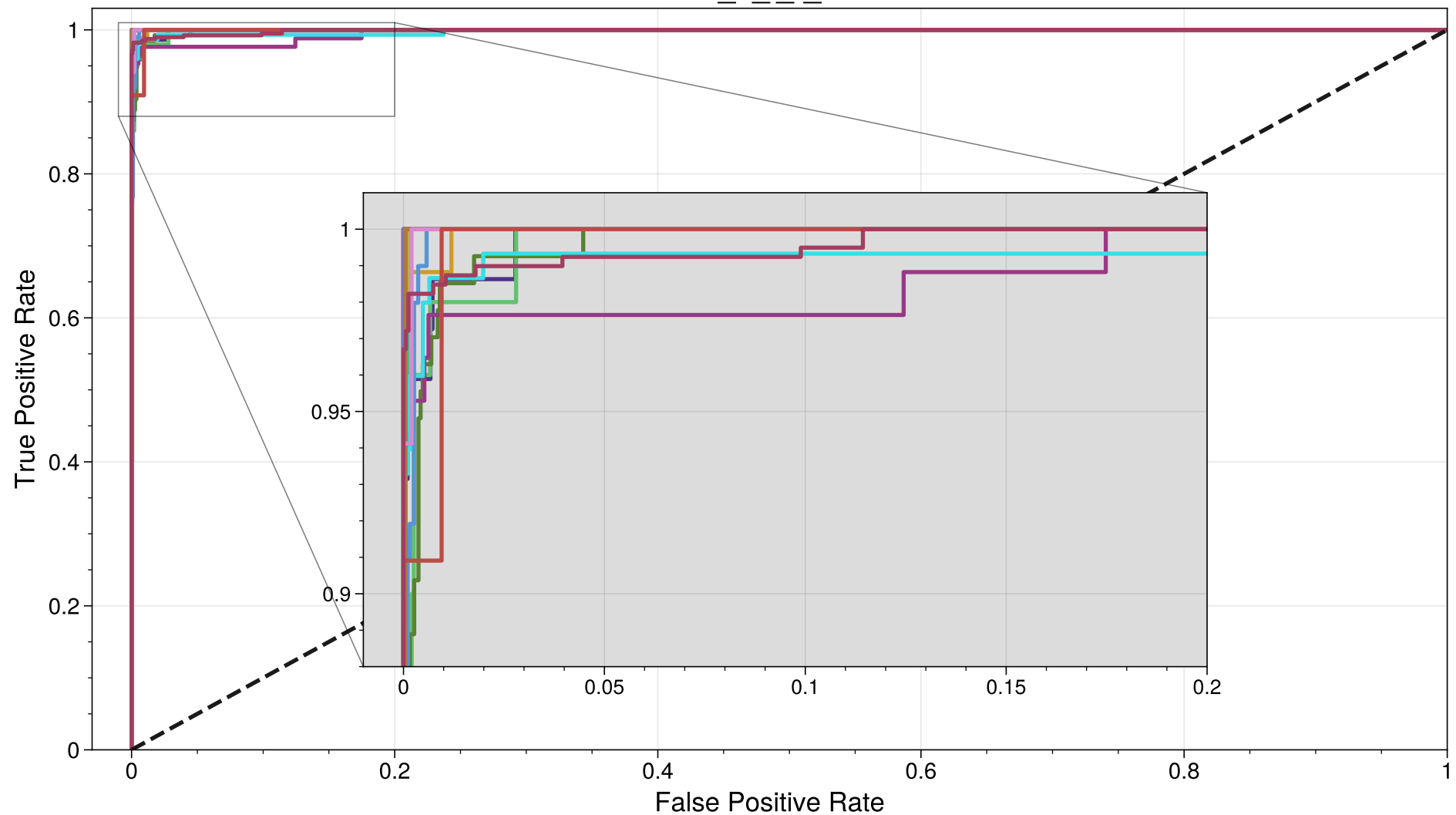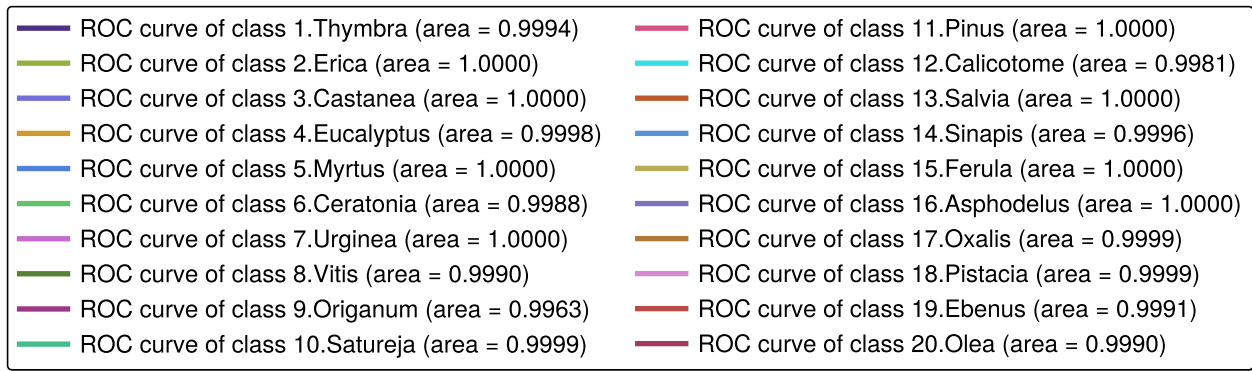

Supplement: Supplementary file 1 [file plants-11-00919-s001.zip › Supplementary-Images/roc-curves-of-all-models/ens_ir_i_r_soft_roc.pdf]

ens\_i\_r\_soft

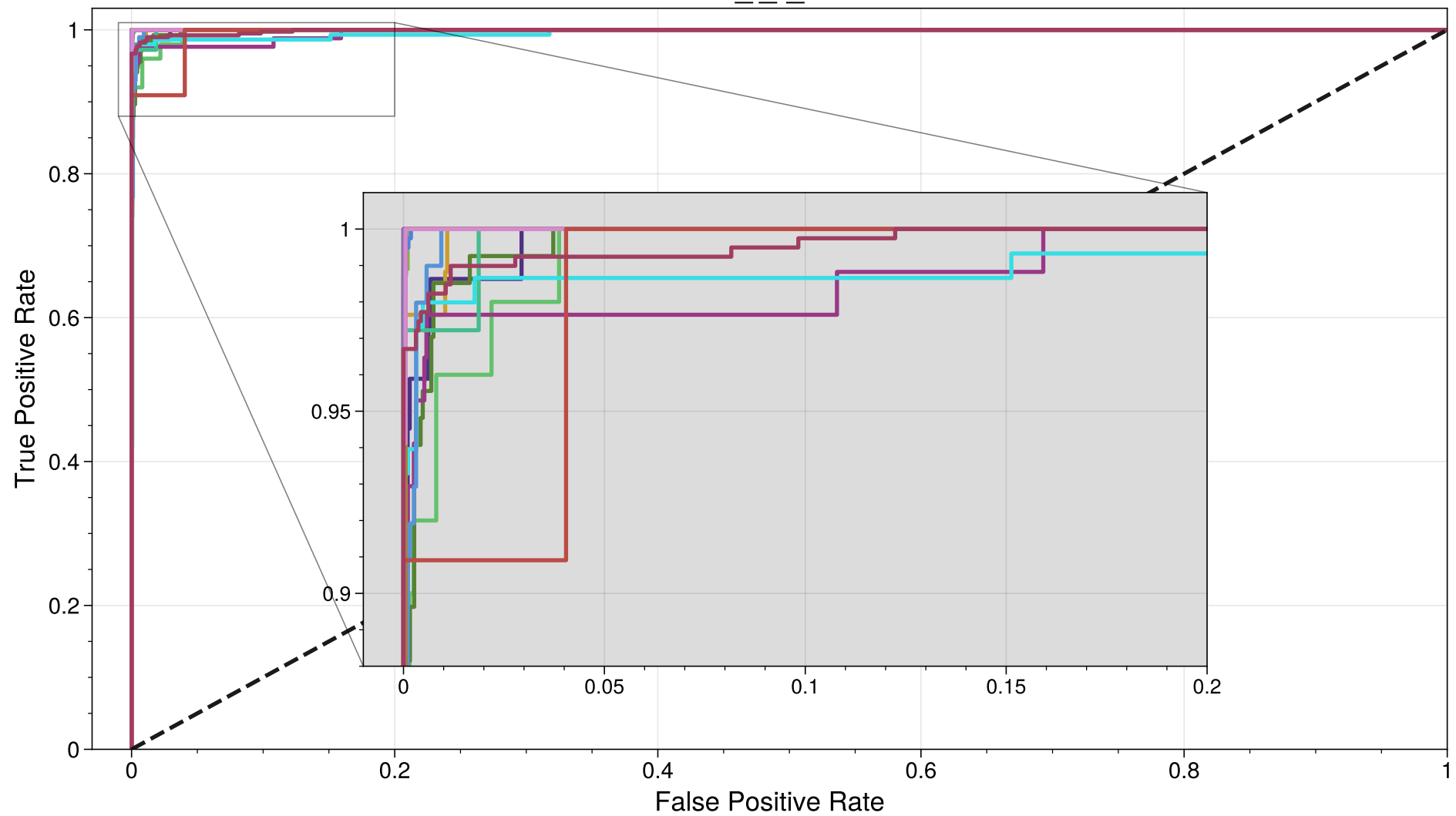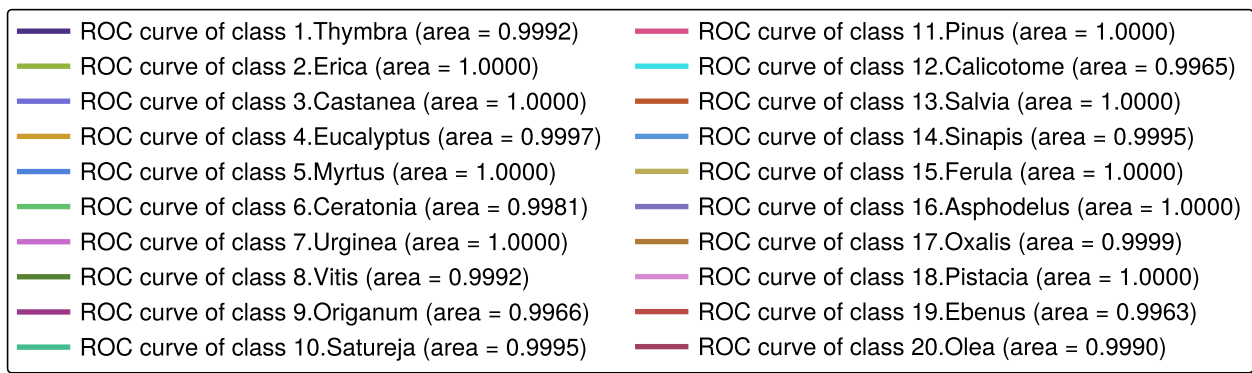

Supplement: Supplementary file 1 [file plants-11-00919-s001.zip › Supplementary-Images/roc-curves-of-all-models/ens_i_r_soft_roc.pdf]

ens\_ir\_r\_soft

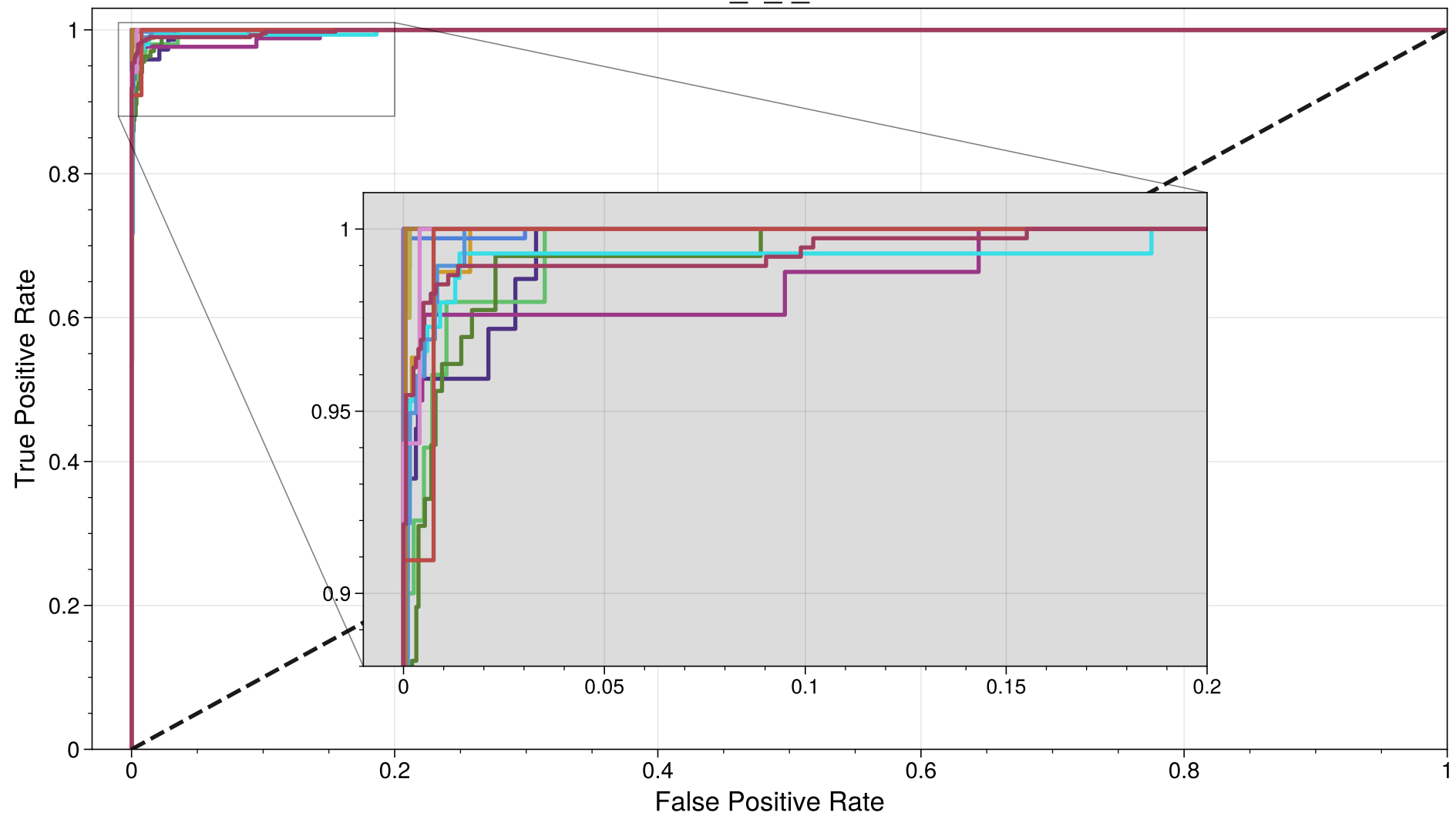

Supplement: Supplementary file 1 [file plants-11-00919-s001.zip › Supplementary-Images/roc-curves-of-all-models/ens_ir_r_soft_roc.pdf]

ens\_x\_ir\_i\_soft

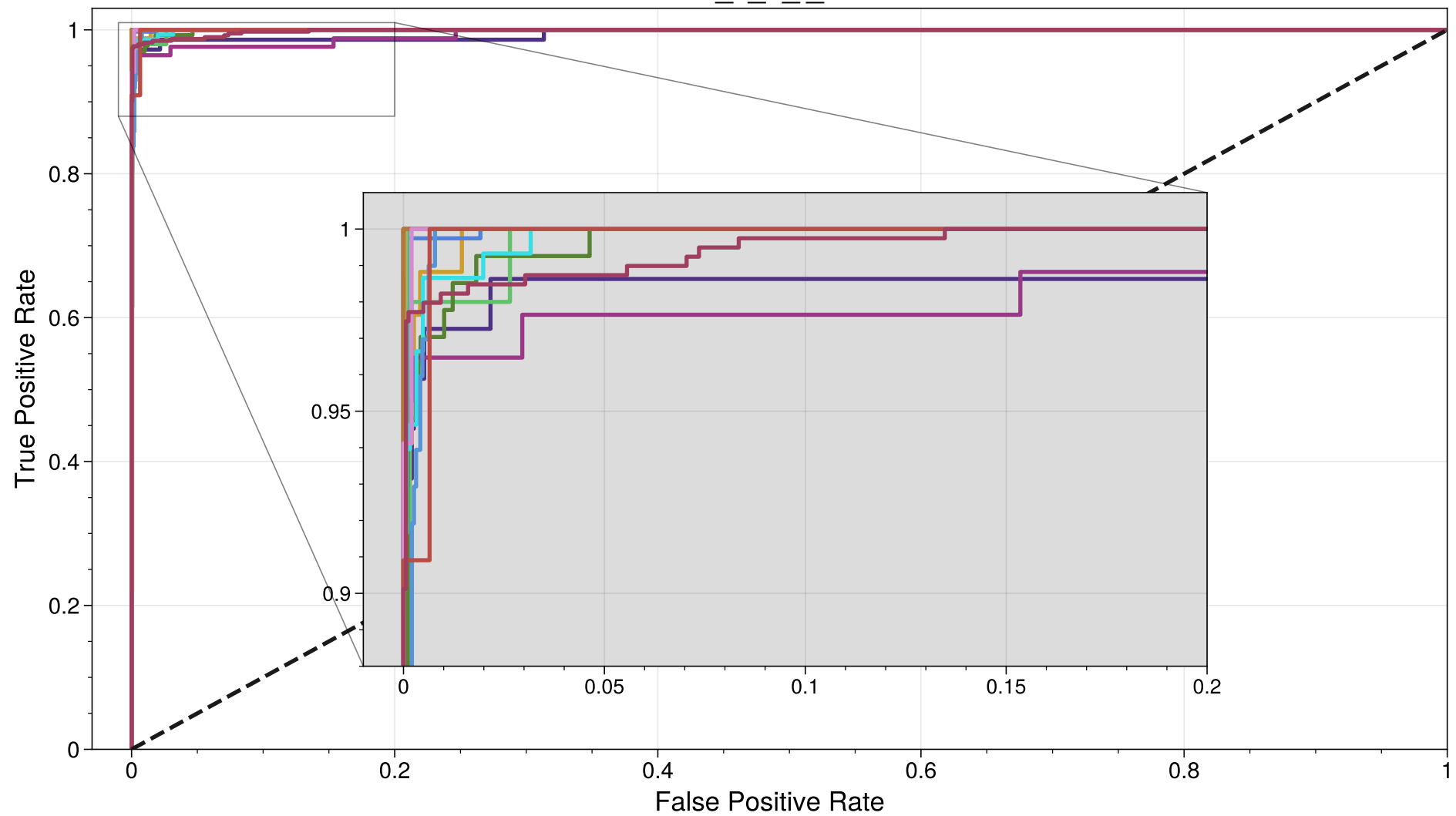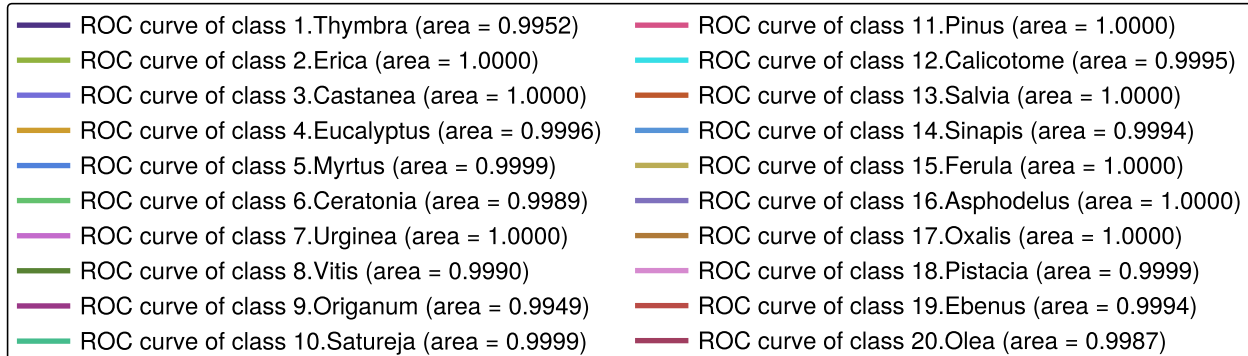

Supplement: Supplementary file 1 [file plants-11-00919-s001.zip › Supplementary-Images/roc-curves-of-all-models/ens_x_ir_i_soft_roc.pdf]

ens\_x\_ir\_r\_soft

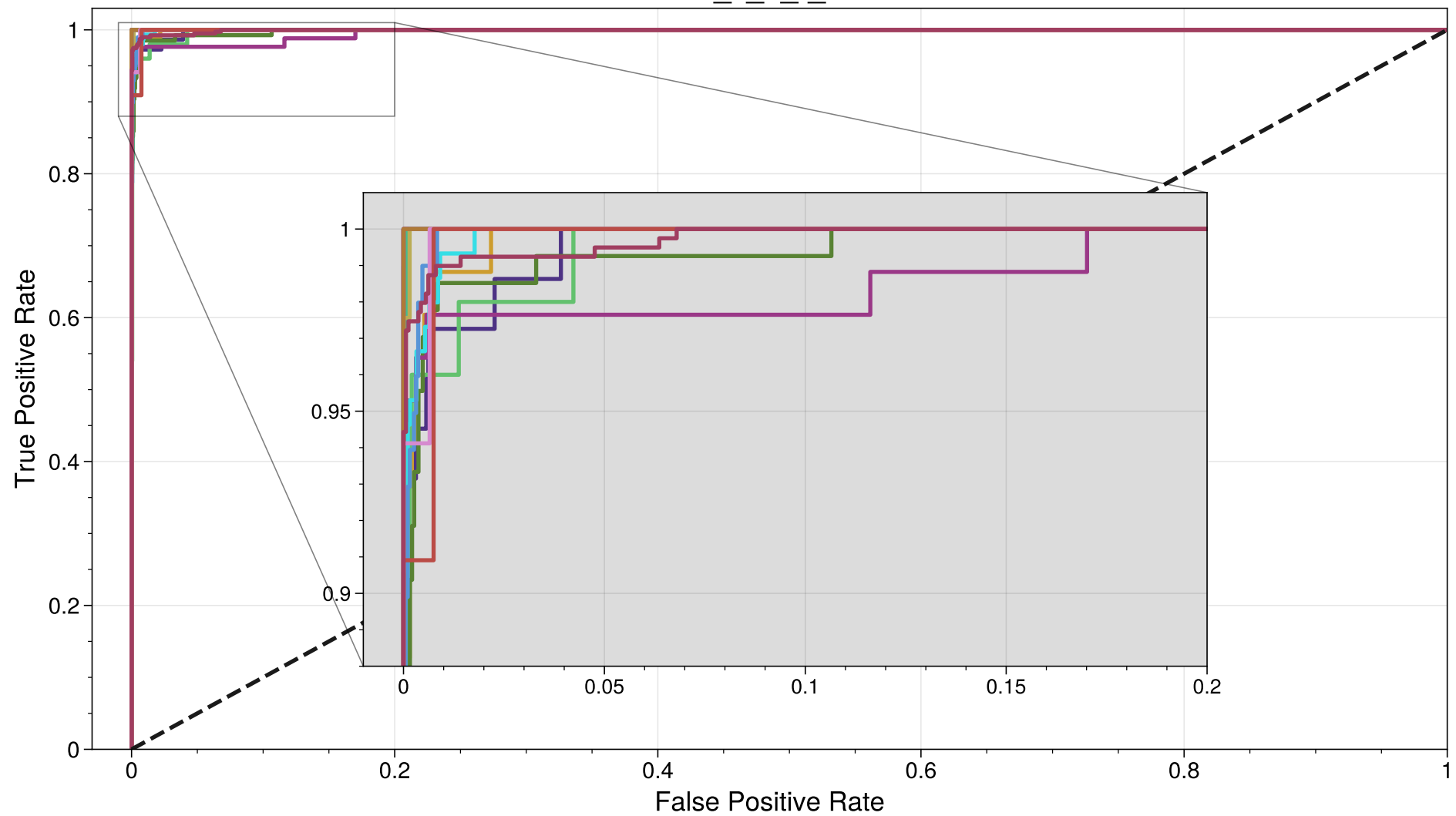

Supplement: Supplementary file 1 [file plants-11-00919-s001.zip › Supplementary-Images/roc-curves-of-all-models/ens_x_ir_r_soft_roc.pdf]

ens\_x\_ir\_soft

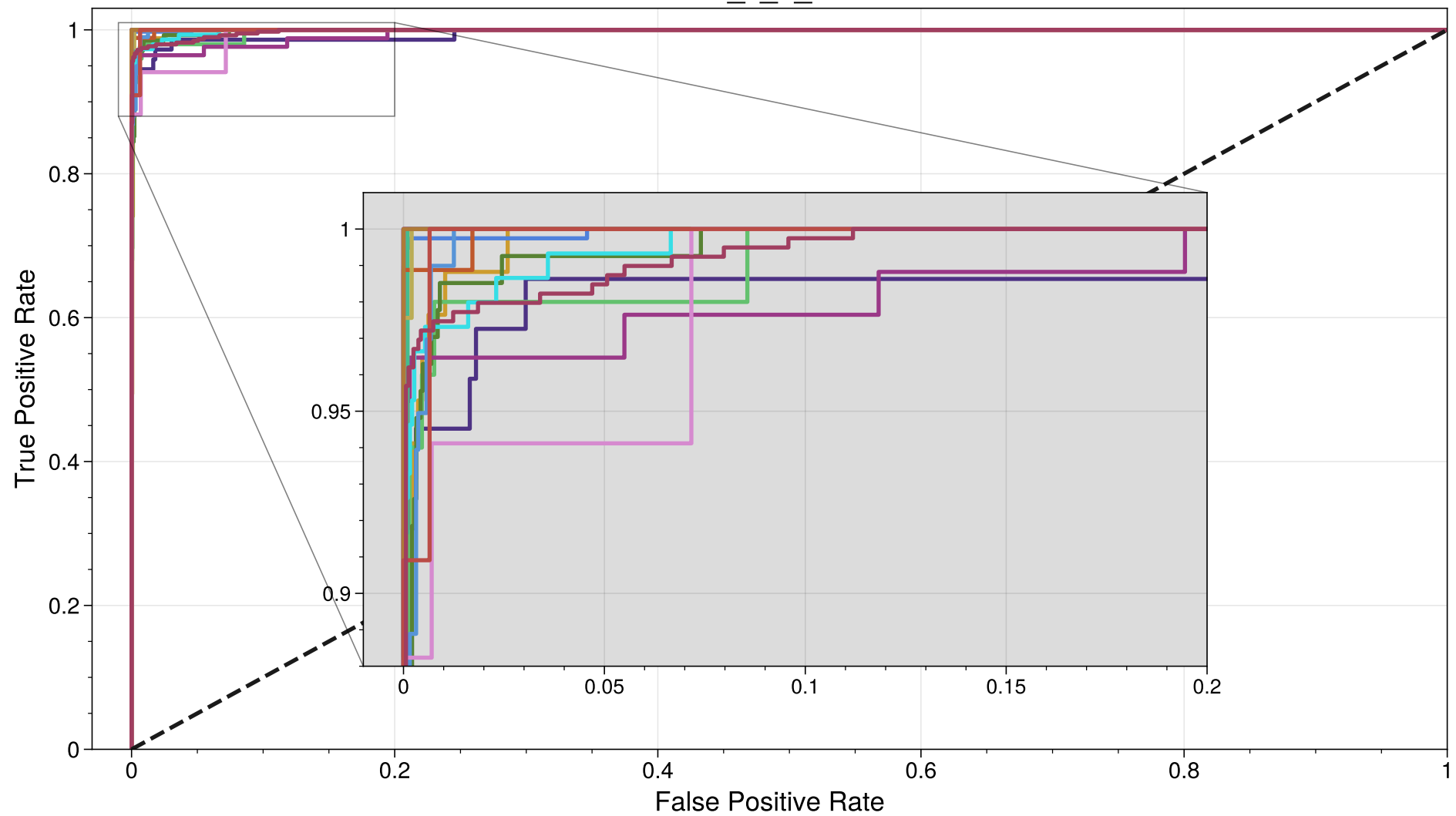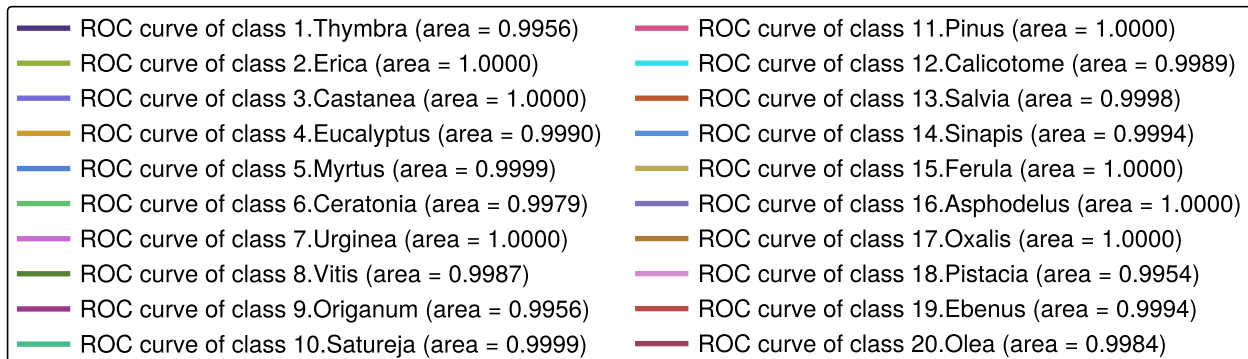

Supplement: Supplementary file 1 [file plants-11-00919-s001.zip › Supplementary-Images/roc-curves-of-all-models/ens_x_ir_soft_roc.pdf]

ens\_x\_i\_soft

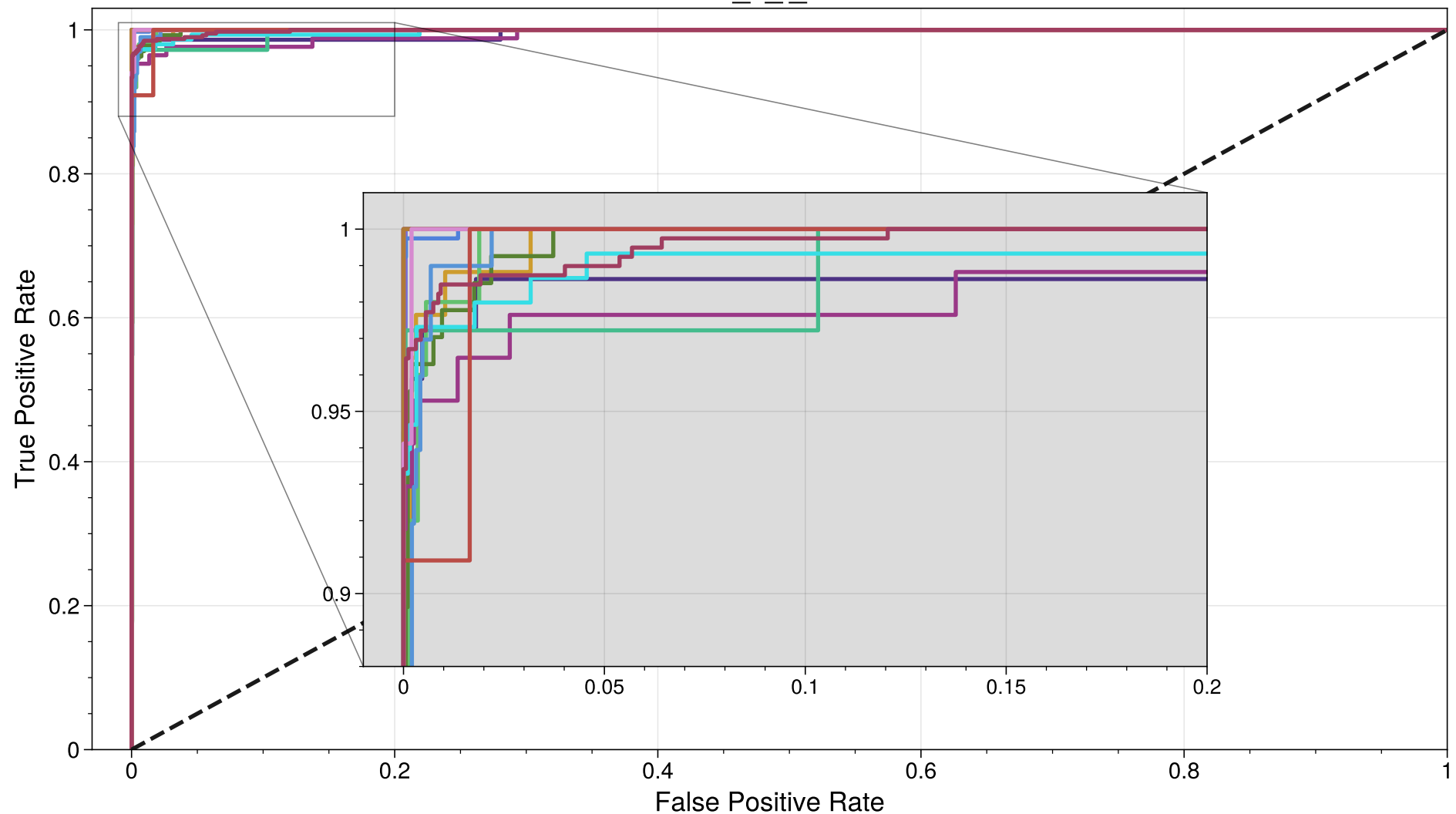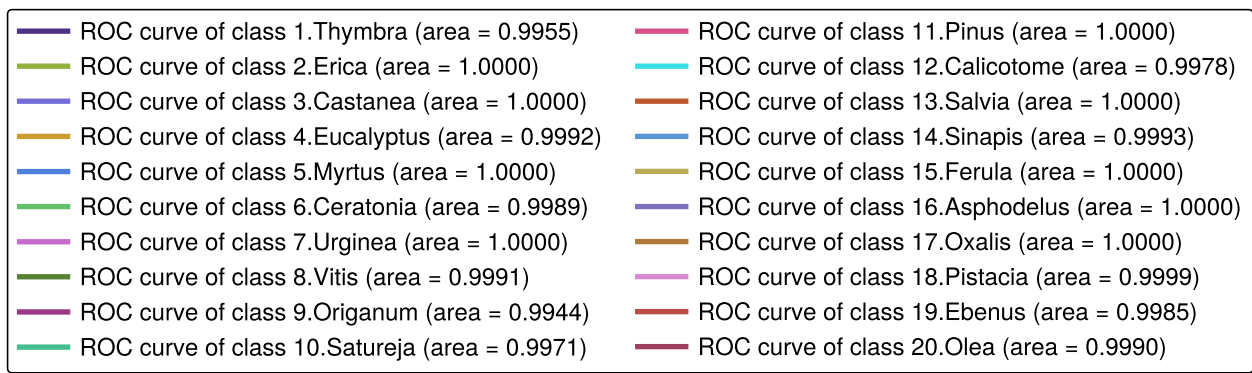

Supplement: Supplementary file 1 [file plants-11-00919-s001.zip › Supplementary-Images/roc-curves-of-all-models/ens_x_i_soft_roc.pdf]

ens\_x\_r\_soft

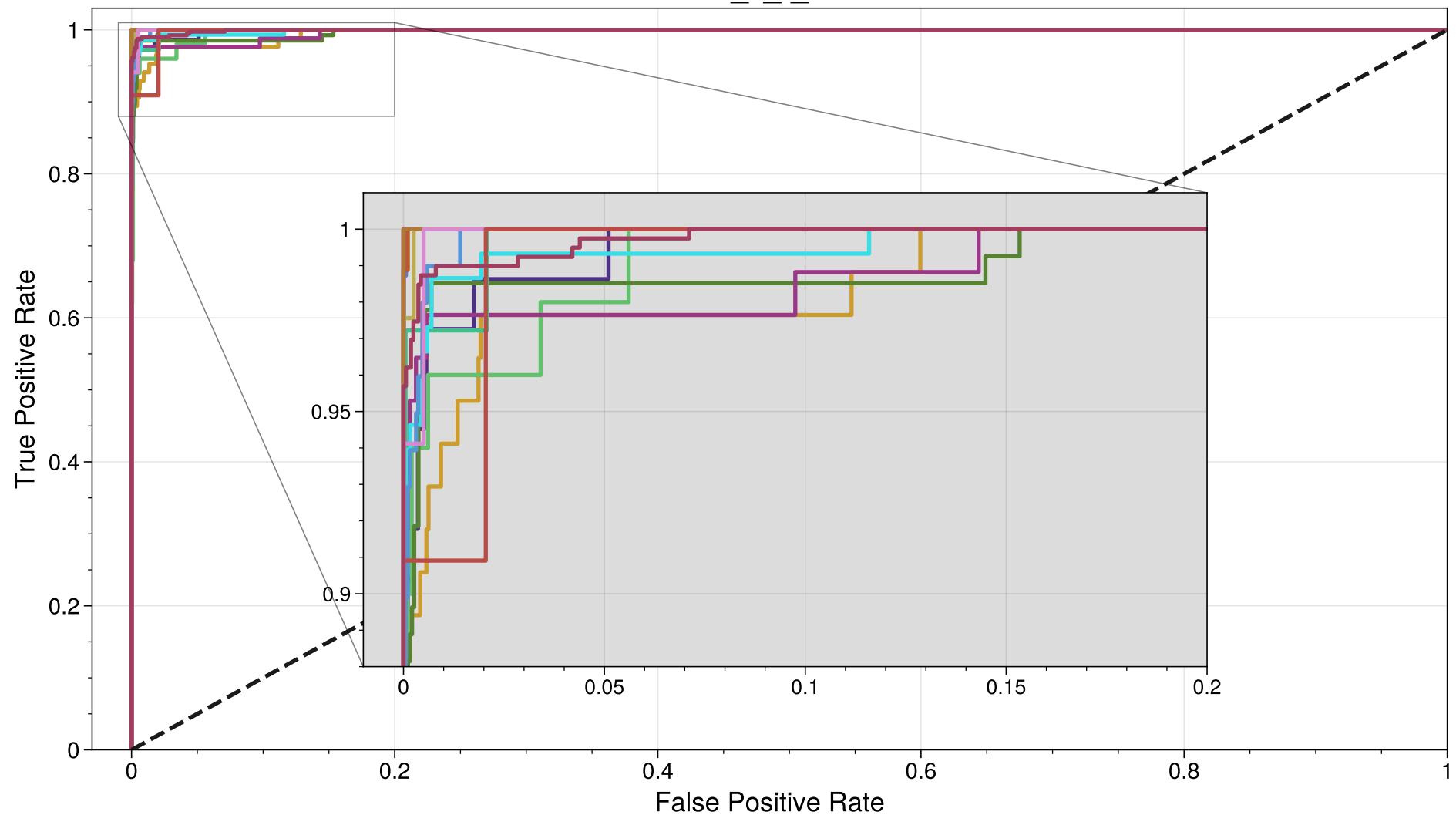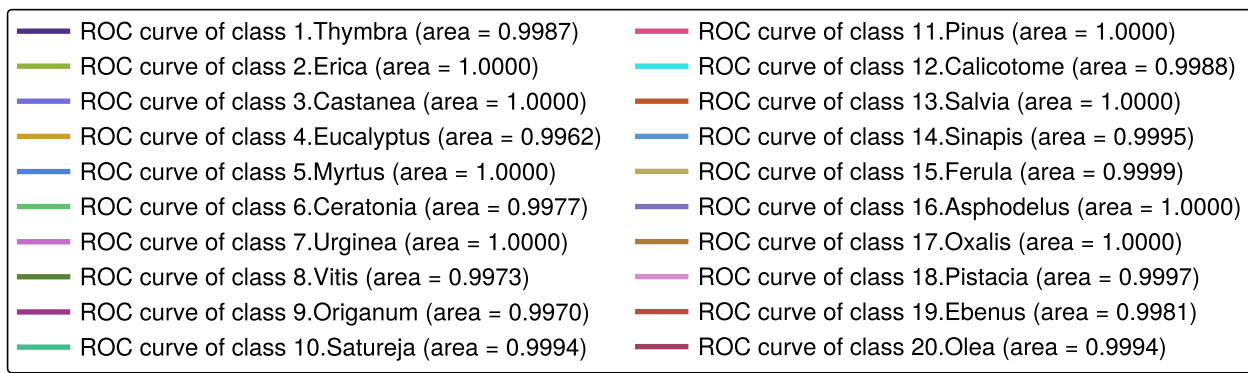

Supplement: Supplementary file 1 [file plants-11-00919-s001.zip › Supplementary-Images/roc-curves-of-all-models/ens_x_r_soft_roc.pdf]

# inception

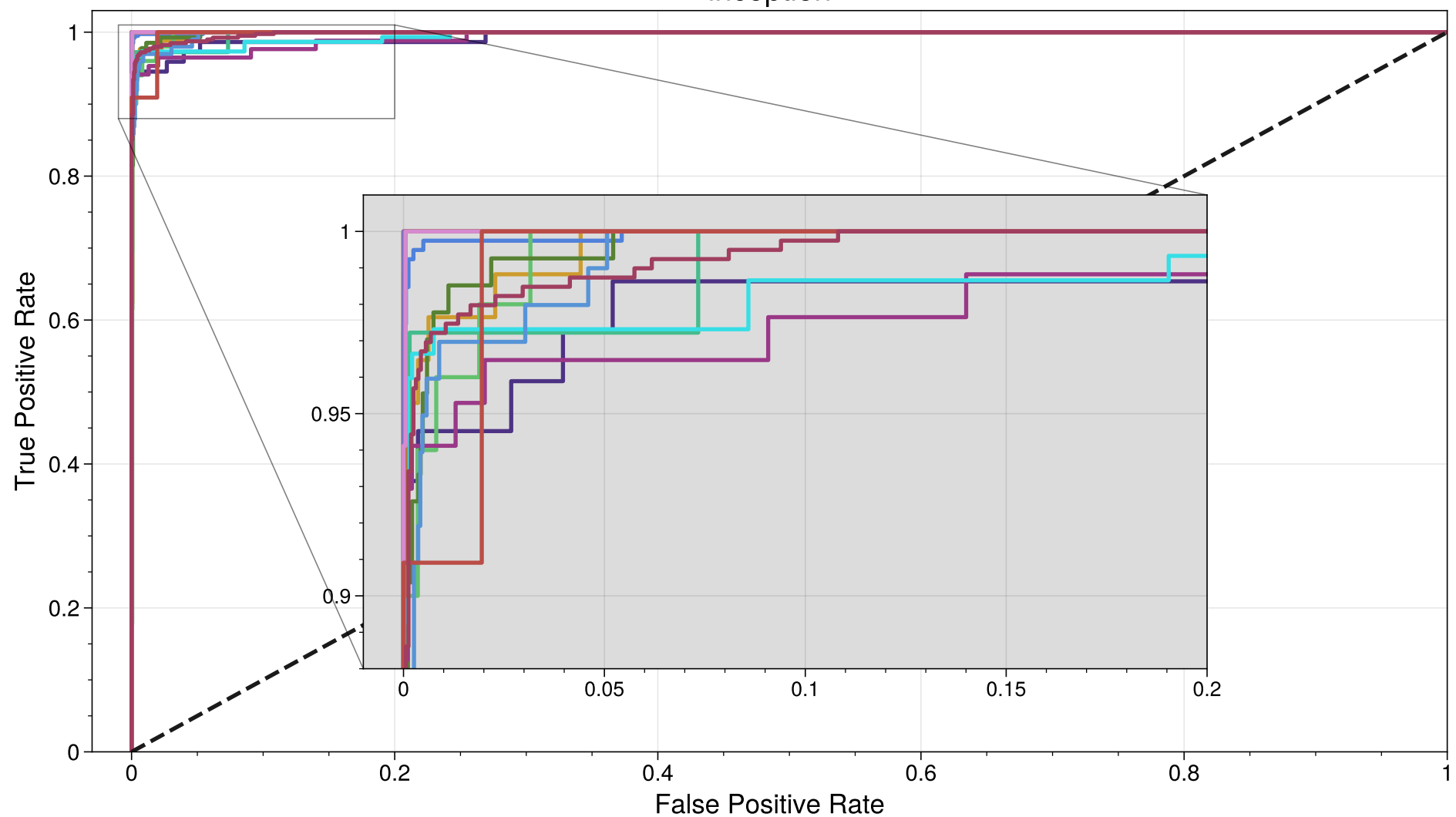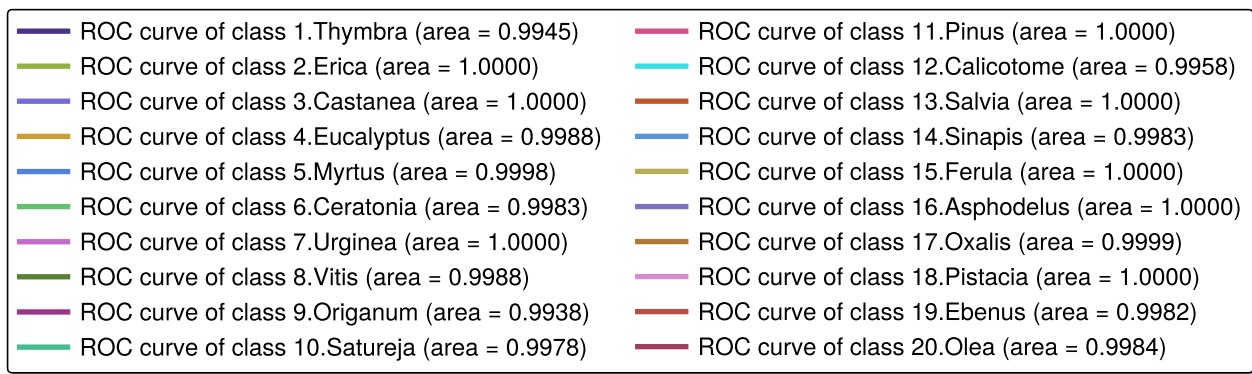

Supplement: Supplementary file 1 [file plants-11-00919-s001.zip › Supplementary-Images/roc-curves-of-all-models/inception_roc.pdf]

inception\_resnet

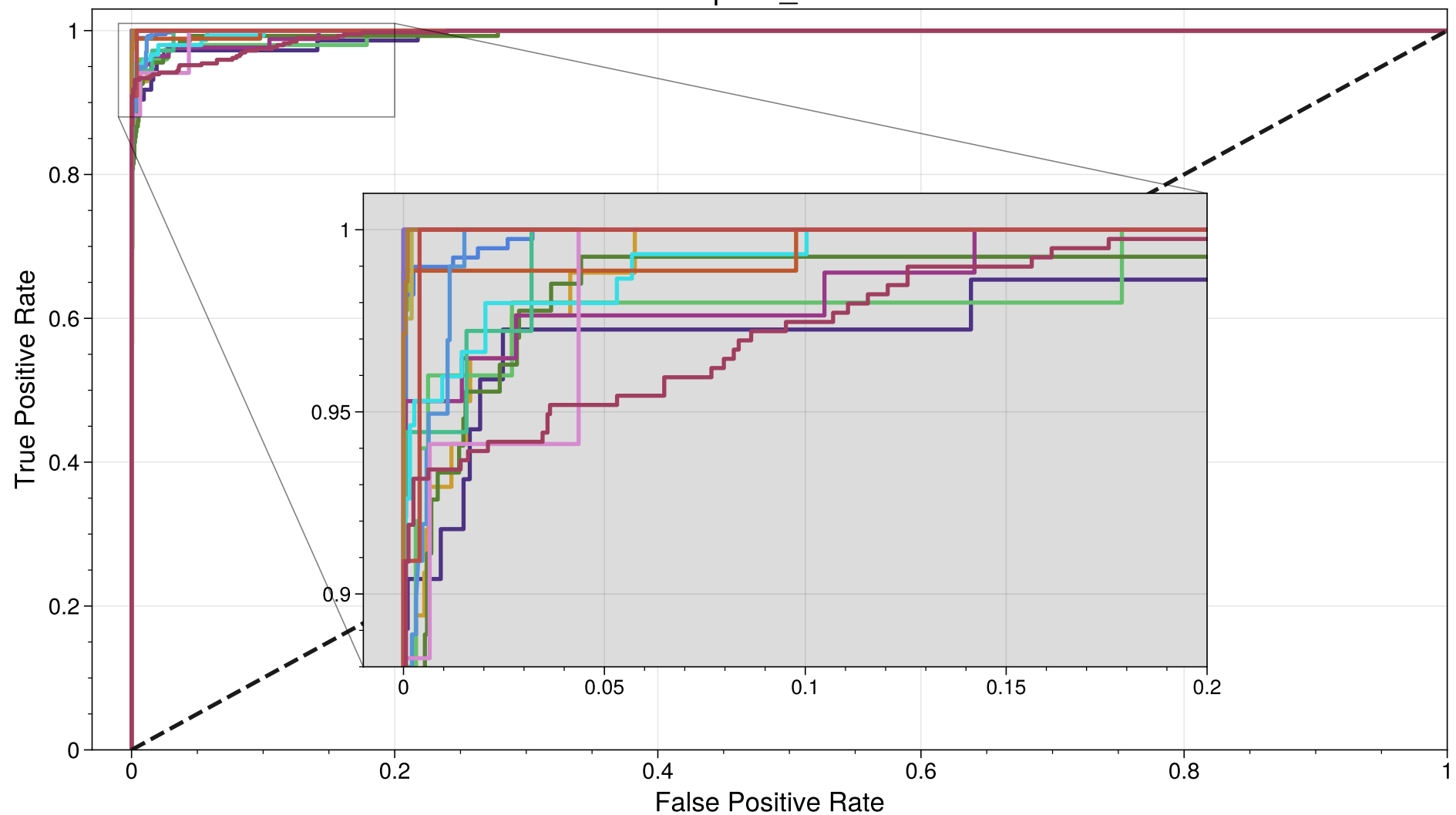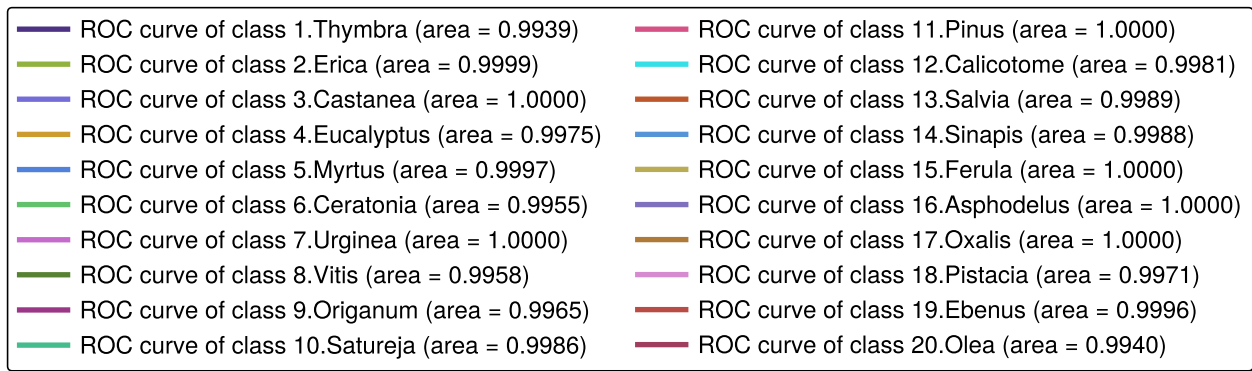

Supplement: Supplementary file 1 [file plants-11-00919-s001.zip › Supplementary-Images/roc-curves-of-all-models/inception_resnet_roc.pdf]

# xception

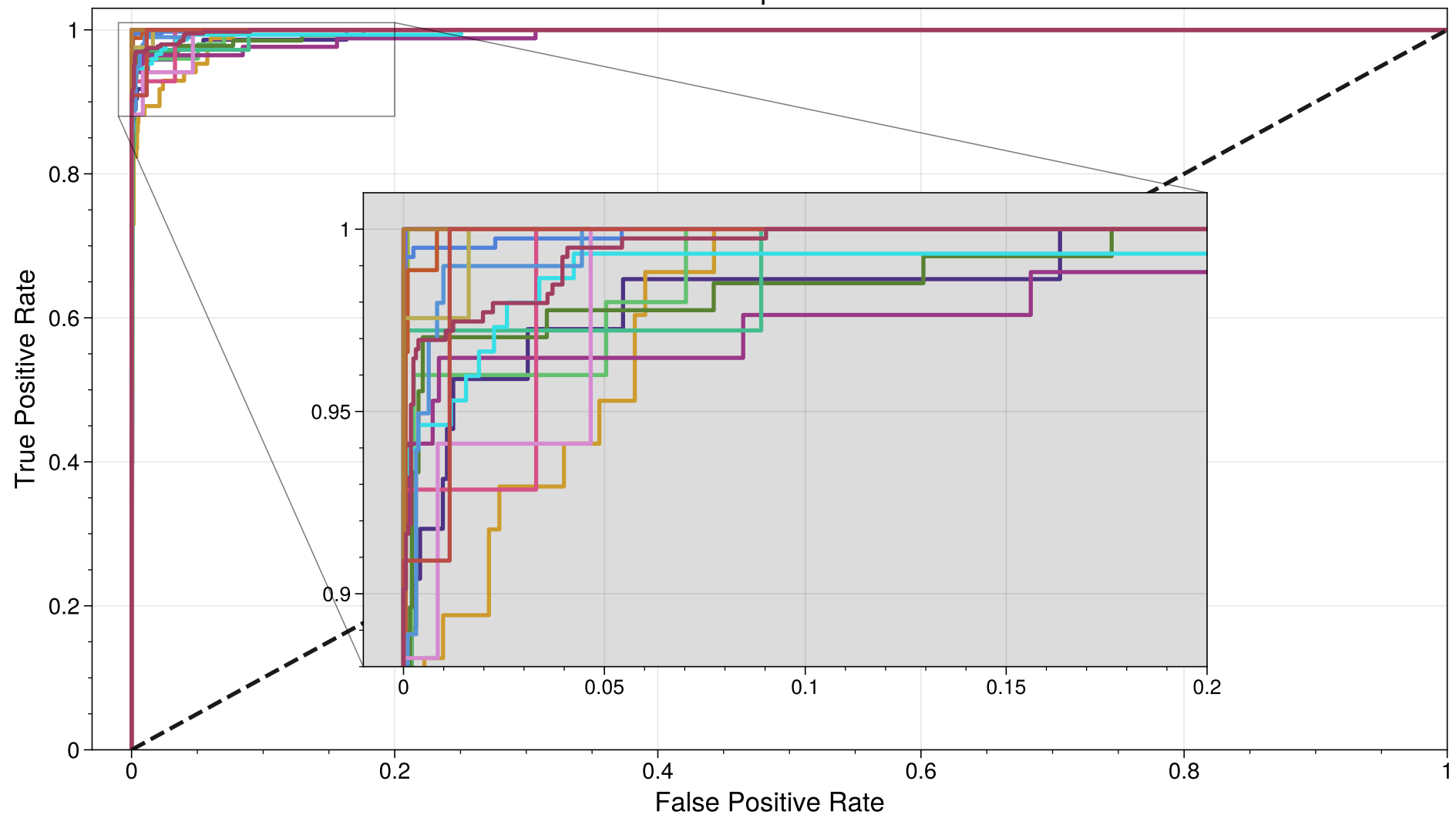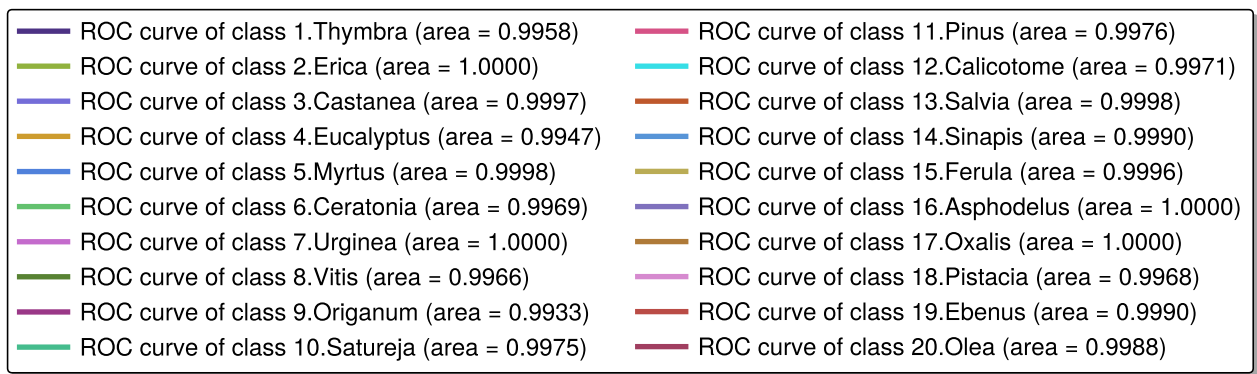

Supplement: Supplementary file 1 [file plants-11-00919-s001.zip › Supplementary-Images/roc-curves-of-all-models/xception_roc.pdf]
